# Supplementary material for: Partially randomised patient preference trials as an alternative design to randomised controlled trials: systematic review and meta-analyses
Source: BMJ Open. 2019 Oct 16;9(10):e031151. doi: 10.1136/bmjopen-2019-031151 (PMC6797441; doi:10.1136/bmjopen-2019-031151)
Supplement: Supplementary data [file bmjopen-2019-031151supp002.pdf]

PROSPERO
International prospective register of systematic reviews

National Institute for Health Research

UNIVERSITY of York
Centre for Reviews and Dissemination

Systematic review

1. * Review title.

Give the working title of the review, for example the one used for obtaining funding. Ideally the title should state succinctly the interventions or exposures being reviewed and the associated health or social problems. Where appropriate, the title should use the PI(E)COS structure to contain information on the Participants, Intervention (or Exposure) and Comparison groups, the Outcomes to be measured and Study designs to be included.

Influence of patients' preference in randomised controlled trials

2. Original language title.

For reviews in languages other than English, this field should be used to enter the title in the language of the review. This will be displayed together with the English language title.

3. * Anticipated or actual start date.

Give the date when the systematic review commenced, or is expected to commence.
01/02/2017

4. * Anticipated completion date.

Give the date by which the review is expected to be completed.
12/03/2019

5. * Stage of review at time of this submission.

Indicate the stage of progress of the review by ticking the relevant Started and Completed boxes. Additional information may be added in the free text box provided.
Please note: Reviews that have progressed beyond the point of completing data extraction at the time of initial registration are not eligible for inclusion in PROSPERO. Should evidence of incorrect status and/or completion date being supplied at the time of submission come to light, the content of the PROSPERO record will be removed leaving only the title and named contact details and a statement that inaccuracies in the stage of the review date had been identified.
This field should be updated when any amendments are made to a published record and on completion and publication of the review. If this field was pre-populated from the initial screening questions then you are not able to edit it until the record is published.

The review has not yet started: Yes

PROSPERO
International prospective register of systematic reviews

National Institute for Health Research

Review stage	Started	Completed
Preliminary searches	No	No
Piloting of the study selection process	No	No
Formal screening of search results against eligibility criteria	No	No
Data extraction	No	No
Risk of bias (quality) assessment	No	No
Data analysis	No	No

Provide any other relevant information about the stage of the review here (e.g. Funded proposal, protocol not yet finalised).

6. * Named contact.

The named contact acts as the guarantor for the accuracy of the information presented in the register record.

Karin Wasmann

Email salutation (e.g. "Dr Smith" or "Joanne") for correspondence:

Miss Wasmann

7. * Named contact email.

Give the electronic mail address of the named contact.

k.a.wasmann@amc.nl

8. Named contact address

Give the full postal address for the named contact.

Amsterdam UMC, department of surgery, Meibergdreef 9, 1105 AZ Amsterdam

9. Named contact phone number.

Give the telephone number for the named contact, including international dialling code.

00316-57066120

10. * Organisational affiliation of the review.

Full title of the organisational affiliations for this review and website address if available. This field may be completed as 'None' if the review is not affiliated to any organisation.

Amsterdam UMC

Organisation web address:

11. * Review team members and their organisational affiliations.

Give the title, first name, last name and the organisational affiliations of each member of the review team. Affiliation refers to groups or organisations to which review team members belong.

Miss Karin Wasmann. Amsterdam UMC

12. * Funding sources/sponsors.

Give details of the individuals, organizations, groups or other legal entities who take responsibility for initiating, managing, sponsoring and/or financing the review. Include any unique identification numbers assigned to the review by the individuals or bodies listed.

None

13. * Conflicts of interest.

List any conditions that could lead to actual or perceived undue influence on judgements concerning the main topic investigated in the review.

None

14. Collaborators.

Give the name and affiliation of any individuals or organisations who are working on the review but who are not listed as review team members.

15. * Review question.

State the question(s) to be addressed by the review, clearly and precisely. Review questions may be specific or broad. It may be appropriate to break very broad questions down into a series of related more specific questions. Questions may be framed or refined using PI(E)COS where relevant.

Influence of patients' preference in randomised controlled trials.

1) Patients’ preference will negatively influence participation to RCTs, decreasing external validity.

Therefore, the external validity of a patient preference trial (PPT) will be higher.

2) Patients’ preferences will influence outcomes in unblinded RCTs, decreasing internal validity. By using a PPT, patients with a preference will be included in the preference cohort and the remaining indifferent patients will be included in the RCT cohort, providing insight in the internal validity.

16. * Searches.

Give details of the sources to be searched, search dates (from and to), and any restrictions (e.g. language or publication period). The full search strategy is not required, but may be supplied as a link or attachment.

A systematic review including meta-analyses of PPTs was conducted. A search in PubMed, Embase, PsycINFO, and the Cochrane Library for PPTs published between Jan 1, 2005 and Oct 5, 2018 was executed without language restriction. The subject in the search strategy was PPT and possible aliases of PPT.

17. URL to search strategy.

Give a link to a published pdf/word document detailing either the search strategy or an example of a search strategy for a specific database if available (including the keywords that will be used in the search strategies), or upload your search strategy.Do NOT provide links to your search results.

https://www.crd.york.ac.uk/PROSPEROFILES/94438_STRATEGY_20190109.pdf

Alternatively, upload your search strategy to CRD in pdf format. Please note that by doing so you are consenting to the file being made publicly accessible.

Do not make this file publicly available until the review is complete

Wasmann KA, *et al. BMJ Open* 2019; 9:e031151. doi: 10.1136/bmjopen-2019-031151

PROSPERO
International prospective register of systematic reviews

National Institute for Health Research

18. * Condition or domain being studied.

Give a short description of the disease, condition or healthcare domain being studied. This could include health and wellbeing outcomes.

Patient preference trials initiated for patients with any condition.

19. * Participants/population.

Give summary criteria for the participants or populations being studied by the review. The preferred format includes details of both inclusion and exclusion criteria.

Clinical trial patients who were ask for treatment preference. If so, they were allocated to the preferred treatment and indifferent patients were randomised.

20. * Intervention(s), exposure(s).

Give full and clear descriptions or definitions of the nature of the interventions or the exposures to be reviewed.

The preference cohort.

21. * Comparator(s)/control.

Where relevant, give details of the alternatives against which the main subject/topic of the review will be compared (e.g. another intervention or a non-exposed control group). The preferred format includes details of both inclusion and exclusion criteria.

The randomised cohort.

22. * Types of study to be included.

Give details of the types of study (study designs) eligible for inclusion in the review. If there are no restrictions on the types of study design eligible for inclusion, or certain study types are excluded, this should be stated. The preferred format includes details of both inclusion and exclusion criteria.

Patient preference trials.

23. Context.

Give summary details of the setting and other relevant characteristics which help define the inclusion or exclusion criteria.

We included PPTs describing results of both the randomised and preference cohort, as long as in both cohorts patients met the same in- and exclusion criteria and were treated according to the same treatment protocol. We excluded trials in which allocation was based on doctors’ preference, without available separate data for the randomised and preference cohort, with economical primary outcomes, or with nonclinical populations. We did not exclude trials based on quality criteria, as no quality assessment for PPTs has yet been developed and current criteria predominantly relate to concealment of randomisation (consequently quality assessment and variability between trials was not applicable). Furthermore, it was decided not to include older PPTs (before 2005), as it is important to consider the value of this design for current daily practice. A previous systematic review addressing on the value of PPTs was published in 2005, which can be used to interpret results from older studies.

24. * Main outcome(s).

Give the pre-specified main (most important) outcomes of the review, including details of how the outcome is defined and measured and when these measurement are made, if these are part of the review inclusion criteria.

The primary outcomes are external and internal validity. Whether patients' preference influences external validity, data will be extracted on participation rates: i) the overall participation rate of eligible patients in the PPT and ii) the proportion of patients accepting randomisation. To assess if a specific patient group accepts randomisation, data will be extracted on baseline characteristics of the randomised and preference cohort of within a PPT separately. These characteristics will be categorised into sociodemographic and clinical factors. Following, these factors will be compared between the randomised and preference cohorts of PPTs. Whether patients' preference influences internal validity, data will be extracted on lost to follow-up, cross-overs, and primary outcomes of the randomised and preference cohort within a PPT separately. Following, these outcomes will be compared between the randomised and preference cohorts of PPTs. The primary outcomes of PPTs will be identified through explicit statements, study hypotheses, reported power analyses, and will be checked="checked" value="1" on similarity with the study protocol. If this is not sufficient, the most likely primary outcome will be chosen by consensus.

Timing and effect measures

To compare the primary outcomes between the randomised and preference cohorts within PPTs, the treatment effect of the experimental vs. control treatment of the randomised cohort will be compared with the treatment effect of the experimental vs. control treatment of the preference cohort.

25. * Additional outcome(s).

List the pre-specified additional outcomes of the review, with a similar level of detail to that required for main outcomes. Where there are no additional outcomes please state 'None' or 'Not applicable' as appropriate to the review

Separate analyses on adjusted and non-adjusted primary outcomes will be performed.

Timing and effect measures

Not applicable

26. * Data extraction (selection and coding).

Give the procedure for selecting studies for the review and extracting data, including the number of researchers involved and how discrepancies will be resolved. List the data to be extracted.

The two first authors will independently screen the citations and abstracts for eligible articles using a pre-piloted standardised data-form (Covidence; Veritas Health Innovation, Melbourne, VIC, Australia). Disagreements will be discussed at steering group meetings. The same two authors will extract data with the use of the same data-form. We will consider multiple publications reporting on the same trial as one single

trial for our analyses.

27. * Risk of bias (quality) assessment.

State whether and how risk of bias will be assessed (including the number of researchers involved and how discrepancies will be resolved), how the quality of individual studies will be assessed, and whether and how this will influence the planned synthesis.

We will not exclude trials based on quality criteria, as no quality assessment for PPTs has yet been developed and current criteria predominantly relate to concealment of randomisation (consequently quality assessment and variability between trials do not apply).

28. * Strategy for data synthesis.

Give the planned general approach to synthesis, e.g. whether aggregate or individual participant data will be used and whether a quantitative or narrative (descriptive) synthesis is planned. It is acceptable to state that a quantitative synthesis will be used if the included studies are sufficiently homogenous.

The level of sought data are summary estimates (aggregate data). A quantative synthesis is planned. To realize the comparison of the primary outcomes of randomised and preference cohorts, probably a reanalysis needs to be conducted. Because the trials probably involved a range of diseases, outcome measures, and sample sizes, different treatment effects scales it is neccesary to convert these into standardised effect sizes in a reanalysis. Treatment effects are calculated directly for continuous outcome variables as standardised mean differences (difference in means divided by the pooled standard deviation). For binary outcomes log odds ratios are calculated and converted into standardised effect size differences. In case none of the patients in the preference cohort choose the control treatment, the treatment effect of the experimental treatment will be compared with the control treatment of the randomised cohort. Only trials for which a ‘net’ effect (primary outcome minus baseline value of the primary outcome) can be calculated, will be included in the meta-analyses. In case the ‘net’ effect is missing, but baseline values and primary outcomes are available, the SD will be estimated. A final meta-regression will be performed using a wald test to compare the standardised treatment effects. A P 0.05 is considered significant difference. R’s programming environment will be used (version 3.5.1 , R Foundation for Statistical Computing, Vienna, Austria). Five researches are involved. Disagreements are discussed at steering group meetings.

29. * Analysis of subgroups or subsets.

Give details of any plans for the separate presentation, exploration or analysis of different types of participants (e.g. by age, disease status, ethnicity, socioeconomic status, presence or absence or co-morbidities); different types of intervention (e.g. drug dose, presence or absence of particular components of intervention); different settings (e.g. country, acute or primary care sector, professional or family care); or different types of study (e.g. randomised or non-randomised).

Adjusted and non-adjusted primary outcomes.

30. * Type and method of review.

Select the type of review and the review method from the lists below. Select the health area(s) of interest for your review.

PROSPERO
International prospective register of systematic reviews

National Institute for Health Research

Type of review

Cost effectiveness
No

Diagnostic
No

Epidemiologic
Yes

Individual patient data (IPD) meta-analysis
No

Intervention
No

Meta-analysis
No

Methodology
No

Narrative synthesis
No

Network meta-analysis
No

Pre-clinical
No

Prevention
No

Prognostic
No

Prospective meta-analysis (PMA)
No

Review of reviews
No

Service delivery
No

Synthesis of qualitative studies
No

Systematic review
Yes

Other
No

Health area of the review

Alcohol/substance misuse/abuse
No

Blood and immune system
No

Cancer
No

Cardiovascular
No

Care of the elderly
No

Child health
No

Wasmann KA, *et al. BMJ Open* 2019; 9:e031151. doi: 10.1136/bmjopen-2019-031151

Complementary therapies
No
Crime and justice
No
Dental
No
Digestive system
No
Ear, nose and throat
No
Education
No
Endocrine and metabolic disorders
No
Eye disorders
No
General interest
Yes
Genetics
No
Health inequalities/health equity
No
Infections and infestations
No
International development
No
Mental health and behavioural conditions
No
Musculoskeletal
No
Neurological
No
Nursing
No
Obstetrics and gynaecology
No
Oral health
No
Palliative care
No
Perioperative care
No
Physiotherapy
No
Pregnancy and childbirth
No
Public health (including social determinants of health)
No
Rehabilitation
No
Respiratory disorders
No
Service delivery
No
Skin disorders
No
Social care
No

PROSPERO
International prospective register of systematic reviews

National Institute for Health Research

Surgery
No
Tropical Medicine
No
Urological
No
Wounds, injuries and accidents
No
Violence and abuse
No

31. Language.

Select each language individually to add it to the list below, use the bin icon to remove any added in error.

There is an English language summary.

32. Country.

Select the country in which the review is being carried out from the drop down list. For multi-national collaborations select all the countries involved.
Netherlands

33. Other registration details.

Give the name of any organisation where the systematic review title or protocol is registered (such as with The Campbell Collaboration, or The Joanna Briggs Institute) together with any unique identification number assigned. (N.B. Registration details for Cochrane protocols will be automatically entered). If extracted data will be stored and made available through a repository such as the Systematic Review Data Repository (SRDR), details and a link should be included here. If none, leave blank.

34. Reference and/or URL for published protocol.

Give the citation and link for the published protocol, if there is one

Give the link to the published protocol.

Alternatively, upload your published protocol to CRD in pdf format. Please note that by doing so you are consenting to the file being made publicly accessible.

No I do not make this file publicly available until the review is complete

Please note that the information required in the PROSPERO registration form must be completed in full even if access to a protocol is given.

35. Dissemination plans.

Give brief details of plans for communicating essential messages from the review to the appropriate audiences.

Do you intend to publish the review on completion?

Yes

36. Keywords.

Give words or phrases that best describe the review. Separate keywords with a semicolon or new line. Keywords will help users find the review in the Register (the words do not appear in the public record but are included in searches). Be as specific and precise as possible. Avoid acronyms and abbreviations unless these are in wide use.

Comprehensive cohort design, patietns preference trial, pateitns'prference, randomised control trials.

Wasmann KA, *et al. BMJ Open* 2019; 9:e031151. doi: 10.1136/bmjopen-2019-031151

PROSPERO
International prospective register of systematic reviews

National Institute for Health Research

37. Details of any existing review of the same topic by the same authors.

Give details of earlier versions of the systematic review if an update of an existing review is being registered, including full bibliographic reference if possible.

38. * Current review status.

Review status should be updated when the review is completed and when it is published. For newregistrations the review must be Ongoing.
Please provide anticipated publication date

Review_Ongoing

39. Any additional information.

Provide any other information the review team feel is relevant to the registration of the review.

I'm very sorry that I wrote the fields #24-#29 in past time during my revisions, I have corrected this. Currently the data extraction is almost done. Since some deley has occured, we think we will finish the data extraction and analyses in March 2019 instead of past November (I've amended this part). We think prospero is a very usefull and valuable registration, therefore we hope you will register the study.

40. Details of final report/publication(s).

This field should be left empty until details of the completed review are available.

Give the link to the published review.

Wasmann KA, *et al. BMJ Open* 2019; 9:e031151. doi: 10.1136/bmjopen-2019-031151
