# Supplementary material for: Partially randomised patient preference trials as an alternative design to randomised controlled trials: systematic review and meta-analyses
Source: BMJ Open. 2019 Oct 16;9(10):e031151. doi: 10.1136/bmjopen-2019-031151 (PMC6797441; doi:10.1136/bmjopen-2019-031151)
Supplement: Supplementary data [file bmjopen-2019-031151supp003.pdf]

Supplement 3, Table. Significant sociodemographic findings preference vs randomised cohorts

Preference cohorts in comparison to randomised cohorts		
Sociodemographic differences		
**Age**	Older[17,27,41,44,52,60]<br>Younger[46,50]	6/34 trials tested<br>2/34
**Gender**	Female[35,50]<br>Male[67]	2/24 trials tested<br>1/24
**Education**	Higher[17,46,51,61]<br>Lower	4/19 trials tested<br>0/19
**Employment**	Yes[14,18,26]<br>No[52]	3/13 trials tested<br>1/13 trials tested
**Race**	Caucasian[14,17,54,56]<br>Non-Caucasian[23]	4/14 trials tested<br>1/14
**Obese**	Yes<br>No[13,41,43,46]	0/7 trials tested<br>4/7
**Smoking**	Yes<br>No[13,46]	0/5 trials tested<br>2/5
**Married**	Yes<br>No[51]	0/9 trials tested<br>1/9
**Experienced**	Yes[27,52,65]<br>No[26]	3/9 trials tested<br>1/9
Clinical differences		
**Clinical problems**	More severe[13,21,23,26,37,54,60]<br>Less severe[14,16,25,32,41,50,51,56,57,61]<br>Not consistent[40,43,67]	7/20 trials tested<br>10/20<br>3/20
