# Supplementary material for: Partially randomised patient preference trials as an alternative design to randomised controlled trials: systematic review and meta-analyses
Source: BMJ Open. 2019 Oct 16;9(10):e031151. doi: 10.1136/bmjopen-2019-031151 (PMC6797441; doi:10.1136/bmjopen-2019-031151)
Supplement: Author's manuscript [file bmjopen-2019-031151.draft_revisions.pdf]

BMJ Open is committed to open peer review. As part of this commitment we make the peer review history of every article we publish publicly available.

When an article is published we post the peer reviewers' comments and the authors' responses online. We also post the versions of the paper that were used during peer review. These are the versions that the peer review comments apply to.

The versions of the paper that follow are the versions that were submitted during the peer review process. They are not the versions of record or the final published versions. They should not be cited or distributed as the published version of this manuscript.

BMJ Open is an open access journal and the full, final, typeset and author-corrected version of record of the manuscript is available on our site with no access controls, subscription charges or pay-per-view fees (http://bmjopen.bmj.com).

If you have any questions on BMJ Open's open peer review process please email info.bmjopen@bmj.com

BMJ Open

# Influence of patients' preference in randomised controlled trials

| | |
|---|---|
| Journal: | *BMJ Open* |
| Manuscript ID | bmjopen-2019-031151 |
| Article Type: | Research |
| Date Submitted by the Author: | 23-Apr-2019 |
| Complete List of Authors: | Wasmann, Karin; Amsterdam UMC - Locatie AMC, ;<br>Wijsman, Pieta; Spaarne Gasthuis<br>van Dieren, Susan; Amsterdam UMC - Locatie AMC<br>Bemelman, Willem; Amsterdam UMC - Locatie AMC<br>Buskens, Christianne; Amsterdam UMC - Locatie AMC |
| Keywords: | Randomised controlled trial, comprehensive cohort design, internal validity, external validity, patients' preference |
| | |

SCHOLARONE™
Manuscripts

# Influence of patients' preference in randomised controlled trials

*A systematic review and meta-analyses*

Karin A. Wasmann, MD[1], Pieta C. Wijsman[2], MD, Susan van Dieren[3], PhD, Willem A. Bemelman[1*], PhD and Christianne J. Buskens[1], PhD

1. Department of surgery, Amsterdam UMC, University of Amsterdam, Amsterdam, the Netherlands, k.a.wasmann@amsterdamumc.nl, w.a.bemelman@amc.nl, c.j.buskens@amc.nl

2. Department of internal medicine, Spaarne Gasthuis, Hoofddorp, the Netherlands, pwijsman@spaarnegasthuis.nl

3. Department of Epidemiology, University of Amsterdam, Amsterdam, The Netherlands, s.vandieren@amc.nl

*This author is a full professor

**Corresponding author during review process**

Drs. Karin A T G M Wasmann

Department of Surgery, Amsterdam UMC, University of Amsterdam

Meibergdreef 9, 1105 AZ Amsterdam, the Netherlands

k.a.wasmann@amsterdamumc.nl

**Corresponding author**

Dr. Christianne J Buskens

Department of Surgery, Amsterdam UMC, University of Amsterdam

Meibergdreef 9, 1105 AZ Amsterdam, the Netherlands

c.j.buskens@amc.nl

**Word count:** 3605

For peer review only

**Abstract**

**Objective:** Randomised controlled trials (RCT) are the gold standard to provide unbiased data. However, randomly allocating patients to their non-preferred treatment may influence participation and outcomes (e.g. external and internal validity). The aim of this study was to assess the influence of patients' preference in RCTs by analysing comprehensive cohort trials (CCT) in which patients are allocated to a study treatment by randomisation or by patients' preference; a RCT and cohort study combined.

**Design:** Systematic review and meta-analyses.

**Setting:** The search was performed in MEDLINE, Embase, PsychINFO, and the Cochrane library to include CCTs published between Jan, 2005 and Oct, 2018.

**Participants:** CCTs reporting on allocation of patients to random- and preference cohorts, while using the same study protocol for both cohorts were included. Trials were excluded if preference was not recorded.

**Primary and secondary outcome measures:** The main outcomes were the difference in external validity (participation and baseline characteristics) and internal validity (lost to follow-up, cross-over and the primary outcome) between the random cohort versus the preference cohort within each CCT.

**Results:** In total 117 of 3734 identified articles met screening criteria and 44 were eligible (24873 patients). The participation rate in CCTs was >95% in 14 trials(range:48-100%) and the randomisation refusal rate was >50% in 26 trials(range:19-99%). Higher education, female, older age, race, and prior experience with one treatment-arm were characteristics of patients declining randomisation. The lost to follow-up and cross-over rate were significantly higher in the randomised cohort compared to the preference cohort. Following the meta-analysis, the reported primary outcomes were comparable between both cohorts of the CCTs, mean difference 0.093(95%CI:-0.178;0.364, *P*=0.502).

For peer review only

**Conclusions:** Patients' preference led to a substantial proportion of a specific patient group refusing randomisation, while it did not influence the primary outcome within a CCT. Therefore, CCTs could increase external validity without compromising the internal validity compared with RCTs.

**Trial registration:** PROSPERO, #CRD42019094438.

**Key words:** Randomised controlled trials, comprehensive cohort design, internal validity, external validity, patients' preference

For peer review only

## Article Summary

### Strengths and limitations of this study

- This systematic review and meta-analyses of CCTs provide unique data on external and internal validity between randomised and patients' preference cohorts, as in the CCTs patients in the preference cohort were followed according the same conditions as the patients in the randomised cohorts.
- A limitation of our review is that interventions and settings between CCTs were very diverse. However, because of this diversity, the study results apply to a broad area of medicine.
- Concerning the assessment of internal validity, none of the primary outcomes of trials included in the meta-analyses were objective and thought to be 'non-influenceable' by a patient's experience of the treatment. Nevertheless, as it is supposed preference would more likely affect subjective outcomes, evaluating objective outcomes as e.g. mortality is of less interest.
- Concerning the assessment of external validity, it should be noted that in only a minority of trials the differences in sociodemographic and clinical parameters between the cohorts of a CCT were evident.
- It was not possible to objectively establish the quality of included trials, as there is currently no valid critical appraisal tool to apply for a CCT.

For peer review only

**Introduction:**

Randomised controlled trials (RCTs) are suggested to provide the most reliable evidence for treatment efficacy.[1] However, an RCT may be the inappropriate design for any unblinded trial comparing treatments of significant different nature (e.g. medical *vs* surgical). In such cases, it can be expected that many eligible patients decline randomisation due to treatment preference. This could limit the generalizability of results to the clinical population (i.e. reduced external validity). Furthermore, trials comparing experimental vs standard treatment, are likely to include patients preferring experimental treatment, as trial participation is not needed for patients preferring standard treatment. Randomisation to the (non-) preferred strategy could influence adherence to treatment protocol or influence subjective outcomes (reduce internal validity). To preclude the influence of patients' preference on validity, a comprehensive cohort trial (CCT) has been designed. Patients with a preference for a treatment strategies will be treated accordingly, whereas only those patients without a distinct preference will be randomised in the usual way.[2] In the era of patients becoming more active participants in research, the use of CCTs increases. The only previous systematic review addressing influence of preference on validity, concluded that this influence was limited.[3] So far, the value of the CCT remains unclear, nor has it been addressed in the Oxford Levels of Evidence (CEBM).[3]

The aim of the study was to assess the influence of patients' preference following randomisation, by comparing randomised cohorts with preference cohorts within all CCTs published since 2005. Two hypotheses were tested: 1) Patients' preference will negatively influence participation to RCTs, decreasing external validity. Therefore, the external validity of a CCT will be higher. 2) Patients' preferences will influence outcomes in unblinded RCTs, decreasing internal validity. However, as only the remaining indifferent patients will be included in the RCT cohort of a CCT, this RCT cohort can be considered as the true gold standard for internal validity.

For peer review only

**METHODS:**

**Search strategy and selection criteria**

A systematic review including meta-analyses of CCTs was conducted. A search in PubMed, Embase, Psycinfo, and the Cochrane Library for CCTs published between Jan 1, 2005 and Oct 5, 2018 was executed without language restriction with the assistance of a librarian. The subject in the search strategy was CCT and possible aliases of CCT (see Pubmed Search Strategy). Database searches were supplemented by hand searching reference lists of relevant articles. Additionally, authors were contacted to seek for data from unpublished studies identified. Non-English-language articles were translated for possible inclusion.

CCTs describing results of both the randomised and preference cohort, as long as in both cohorts patients met the same in- and exclusion criteria and were treated according to the same treatment protocol were included. Trials in which allocation was based on doctors' preference, without available separate data for the randomised and preference cohort, with economical primary outcomes, or with nonclinical populations were excluded. Furthermore, it was decided not to include older CCTs (before 2005), as it is important to consider the value of this design for current daily practice. A previous systematic review addressing on the value of CCTs was published in 2005, which can be used to interpret results from older studies.[3]

The two first authors independently screened the citations and abstracts for eligible articles using a pre-piloted standardised data-form (Covidence; Veritas Health Innovation, Melbourne, VIC, Australia). Disagreements were discussed at steering group meetings.

This study is reported in accordance with the Cochrane Handbook for Systematic Reviews of Interventions[4] and the Preferred Reportion Items for Systematic Reviews and Meta-Analyses statement (supplement 3).[5] The study protocol is available online (supplement 2).

**Data analysis**

The same two authors extracted data with the use of the same data-form. Multiple publications reporting on the same trial were considered as one single trial for these analyses.

For peer review only

The level of sought data were summary estimates. Authors were contacted for further information when necessary. In case they were not forthcoming, the study was included in the review, but excluded from our reanalysis and or meta-analyses.

The primary outcomes were external and internal validity between randomised and preference cohorts within CCTs. Whether patients' preference influenced external validity, data was extracted on participation rates: i) the overall participation rate of eligible patients in the CCT and ii) the proportion of patients accepting randomisation. To assess if a specific patient group accepted randomisation, data was extracted on baseline characteristics of the randomised and preference cohort of a CCT separately. These characteristics were categorised into sociodemographic and clinical factors. Following, these factors were compared between the randomised and preference cohorts of CCTs.

Whether patients' preference influenced internal validity, data was extracted on lost to follow-up, cross-overs, and primary outcomes of the randomised and preference cohort of a CCT separately. Following, these outcomes were compared between the randomised and preference cohorts of CCTs. The primary outcomes of CCTs were identified through explicit statements, study hypotheses, reported power analyses, and were checked on similarity with the study protocol. If this was not sufficient, the most likely primary outcome was chosen by consensus (KW and SvD), or the study was excluded. The primary outcomes were categorised into subjective and objective outcomes. Subjective outcomes were defined as measures of perception or satisfaction, including reported symptoms or behaviour (directly through self-report, or indirectly through clinical or study attendance). Objective outcomes were defined as a measurement unlikely to be influenced by patients' treatment preference, e.g. mortality. To compare the primary outcomes between the randomised and preference cohorts within CCTs, the treatment effect of the experimental vs. control treatment of the randomised cohort was compared with the treatment effect of the experimental vs. control treatment of the preference cohort. It is emphasized that comparisons of outcome between randomised and preference cohorts are subject to bias, and if not done by the study itself, it was not possible to adjust for confounding factors. If in studies the adjusted and non-adjusted primary outcomes were available, the adjusted outcomes were used. Following, separate analyses on adjusted and non-adjusted primary outcomes were performed.

For peer review only

Quality assessment of the trials was not performed, as no quality assessment for CCTs has yet been developed and current criteria predominantly relate to concealment of randomisation (e.g. ROBINS-I and Cochrane risk of bias) consequently quality assessment and variability between trials was not applicable.[6,7] Since the outcomes of each trial greatly differed, also the risk of bias assessment for systematic reviews (e.g. GRADE) was not applicable.[8]

The randomisation rate, participation rate, and difference in baseline characteristics between the randomised and preference cohorts were explored and described, but not compared using statistics. To assess differences in baseline characteristics, mean and SDs were compared. If median IQRs were reported, it was converted to mean and SDs.[9] When baseline characteristics were presented per experimental and control group, the sum of mean and SDs of these two groups were calculated for the randomised and preference cohorts using weighted t-test. The lost to follow-up and cross-over rates were compared using a random effect model meta-analysis for proportions.

To realise the comparison of the primary outcomes of randomised and preference cohorts, a reanalysis was conducted. Because the trials involved a range of diseases, outcome measures, and sample sizes, different treatment effects scales were converted into standardised effect sizes in the reanalysis. Treatment effects were calculated directly for continuous outcome variables as standardised mean differences (difference in means divided by the pooled standard deviation). For binary outcomes log odds ratios were calculated and converted into standardised effect size differences.[10] In case none of the patients in the preference cohort choose the control treatment, the treatment effect of the experimental treatment was compared with the control treatment of the randomised cohort. Only trials for which a ‘net’ effect (primary outcome minus baseline value of the primary outcome) could be calculated, were included in the meta-analyses. In case the ‘net’ effect was missing, but baseline values and primary outcomes were available, the SD was estimated.[11] Heterogeneity was not assessed as trials outcomes were different for each study included. Meta-analysis of randomised versus preference cohort was performed using a random effect model with an inverse variance weighting. A final meta-regression was performed using a wald test to compare the standardised treatment effects.

A $P< 0.05$ was considered a significant difference. R’s programming environment was used (version 3.5.1 , R Foundation for Statistical Computing, Vienna, Austria).

For peer review only - http://bmjopen.bmj.com/site/about/guidelines.xhtml

**Patient and Public Involvement**

The Dutch Crohn and Colitis patient federation (CCUVN) was involved when we were exploring alternative designs for future studies. As in a CCT also patients with a treatment preference are included, the CCUVN found it important to analyse the validity of this design. As it is a systematic review, the burden of trial participation was not applicable. The results of this review are available at the CCUVN website.

For peer review only

## RESULTS

In total 117, out of 3734 records identified, were full-text screened. Fifty-eight comprehensive cohort trials from 2005 onwards were found, of which 44 (including 24 873 patients) were eligible for at least basic data extraction (Table 1), and 20 could be included in the meta-analyses (Prisma flowchart Figure 1).[12–69] Exclusion reasons for the meta-analyses were, no availability of both treatment outcomes in the randomised and preference cohort separately in 14 trials[12,13,16,19,20,23,29,31,32,35,46,55,68,69], no availability of standard deviations, which could also not be converted from other available data in five trials[18,40,43,54,66], and the number of events or the power of one or both cohort(s) was too low to perform separate randomised and preference analyses in five trials.[14,17,30,47,65] The trials covered a wide range of clinical areas and interventions. The main areas were Gynaecology (n= 11), Orthopaedics (n= 10), and Psychiatry (n= 5). Of the 44 included trials, 32 trials compared an intervention versus conservative treatment, including 16 surgical interventions (Table 1). In all trials but one, if patients refused randomisation they received their preference treatment (Figure 2). In the other study a Zelen Randomisation was performed, randomising all eligible patients and afterwards asking for their consent to participate in the randomised arm or if they preferred the other intervention.[23] Parental preference was relevant in five trials involving children, as permission of parents was required and the preference between patients and parents could not be distinguished.[18,32,48,55,69]

*External validity*

Following results concern the influence of patients' preference on external validity. Information on the number of eligible patients who agreed to participate (in either the randomised or preference cohort), was available in 39 out the 44 CCTs. The participation rate of eligible patients in the CCTs ranged from 48% to 100%. In which 16 CCTs reported a participation rate higher than 80%, and 14 CCTs a participation rate higher than 95%. Of these included participants in the 44 CCTs, 18% to 99% declined randomisation (hence these patients were included in the preference cohort). The randomisation refusal rate was more than 50% in 26 CCTs.

To assess if a specific patient group accepted randomisation, 35 of the 44 CCTs reported at least one comparison between randomised and preference cohorts on baseline sociodemographic factors. At

For peer review only

least one significant difference between randomised and preference cohorts was found in 20 of the 35 trials. Overall, 38 significant differences were found in 161 sociodemographic comparisons (24%). The proportion of significant findings was not dependent on sample size (smaller trials n< 300; 19/85, 22% and larger trials n≥ 300; 19/76, 25%). Patients with a preference compared with those accepting randomisation were more likely to be older, female, higher educated, employed, Caucasian, not obese, non-smokers, unmarried, and experienced with one treatment arm (Supplementary Table 1).

Thirty-four of the 44 CCTs reported at least one comparison between randomised and preference cohorts on clinical baseline characteristics. At least one significant difference was found in 20 of the 34 trials. Overall, 36 significant differences were found in 220 clinical comparisons (16%). The proportion of significant findings was not dependent on sample size (smaller trials n< 300; 12/78, 15% and larger trials n≥ 300; 24/142, 17%). Patients with a preference had more severe clinical problems in seven trials and less severe clinical problems in ten trials, while in the remaining three trials no consistent pattern could be found (Supplementary Table 1).

*Internal validity*

Following results concern the influence of patients' preference on internal validity. Information on lost to follow-up in both the randomised and preference cohorts was available in 33 of the 44 CCTs. For the randomised cohorts, the proportion of individuals lost to follow-up was < 10% in 14 trials, 10% to < 20% in 9 trials, and ≥ 20% in 10 trials. For the preference cohorts the corresponding numbers of trials were 17, 9, and 7. The mean percentage of participants lost to follow-up was significantly higher in the randomised cohorts (16·1% (SD 16·8%)) compared with the preference cohorts (13.3% (SD 14.7%)), RR 1.3, (CI95% 1.0 – 1.6), $P = 0.03$).

Information on cross-overs in both the randomised and preference cohorts was available in 20 of 44 CCTs. For the randomised cohorts, the proportion of individuals that crossed-over to the other study treatment was < 10% in 11 trials, 10% to < 20% in 5 trials, and ≥ 20% in 4 trials. For the preference cohorts the corresponding numbers of trials were 14, 5, and 1. The mean percentage of cross-overs was significantly higher in the randomised cohorts (14.5% (SD 16.9%)) compared with the preference cohorts (6.3**%** (SD 11.5%)), RR 2.6 (CI95% 1.7-3.9), $P < 0.001$).

For peer review only

To assess the influence of patients' preference on primary outcomes, for 20 of the 44 CCTs it was possible to perform reanalyses using standardised effect sizes (Figure 1). In all these trials the primary outcome was subjective.

Figure 3 shows the magnitude of the experimental treatment effect over the control treatment effect of the randomised and preference cohort separately using standardised effect sizes. The trial are listed by sample size. A positive experimental treatment effect was seen in 13 trials. The influence of patients' preference on primary outcomes according to different standardised treatment effects between randomised and preference cohorts was small, in 13 of the 20 trials (65%) this was 0.2 or less (scale -2 to 2), in 5 trials (25%) between 0.21 and 0.5, and in 2 trials (10%) higher than 0.5. Of the 20 CCTs, the overall mean difference in primary outcome between randomised and preference cohorts was not significantly different, 0.093 (95%CI -0.178 to 0.364) $P$ = 0.502 (Figure 2). Only two trials showed a significant different treatment effect between the randomised and preference cohort.[61,62] In both trials the experimental treatment effect was favourable over the control treatment effect in both in the randomised and preference cohort, but the favourable effect of the experimental treatment was significantly greater in the preference cohort. Both CCTs compared acupuncture versus conservative treatment. In one trial the improvement of the osteoarthritis index in patients with osteoarthritis of the knee or hip was assessed, the other trial assessed the functional ability score in patients with chronic low back pain.

In seven of these 20 trials, an adjusted primary outcome for baseline confounders was available[21,25,27,52,56,58,67] In these trials, the mean difference in primary outcome between randomised and preference cohorts was even smaller -0.026 (95%CI -0.263 to 0.211) $P$ = 0.832. In 18 trials (also) a non-adjusted primary outcome was available. Using these outcomes, the mean difference in primary outcomes was 0.228 (95%CI -0.117 to 0.572) $P$ = 0.196 (Figure 4 and 5).

For peer review only

**DISCUSSION:**

These study results challenge the current consensus about the hierarchy of study designs. Our results indicate that patients' preference led to a substantial proportion of patient refusing randomisation (refusal of randomisation was more than 50% in 26 trials), while it did not affect the primary outcome of a CCT.

Regarding our first hypothesis, it can be conclude that patients' preference does negatively influence participation to RCTs as demonstrated by the low participation to the randomised cohort in CCTs. The participation in the CCTs was remarkably high (ranging from 48% - 100%), improving external validity when compared with the classic RCT (ranging from <0.001 - 40%).[70] Cautiously, it could be argued that a typical patient group characterised by e.g. higher education, Caucasian race, and non-obese individuals are more likely to refuse randomisation. In contrast, differences in clinical characteristics showed no consistent pattern in the randomised or preference cohorts. Therefore, not including a patients' preference cohort in a trial could result in a potential loss of inclusions of a specific patient group, further decreasing external validity.

Regarding our second hypothesis, it can be conclude that patients' preference does not significantly affect the primary outcome of a CCT, as the primary outcomes of patients in the randomised and preference cohorts were similar. Since patients with a preference are treated accordingly in a CCT, it can be assumed that the randomised cohort of a CCT includes patients indifferent to the type of treatment. Following, it is unlikely that outcomes of randomised patients will be biased by treatment preference. Hence, they could be seen as the gold standard. Lost to follow-up and cross-overs were significantly higher in the randomised cohort compared with the preference cohort. As a result, the data of the preference cohort could be interpreted more easily than the randomised data. Perhaps, consciously choosing a treatment ensures a certain dedication and tolerance for the treatment.

Our results are strengthened by the previous systematic review of King et al, including CCTs from 1966 to 2004. Based on their results, they also postulated that treatment preference influences the willingness to accept randomisation, and that the evidence of its significant affect on internal validity is low.[3] A possible limitation of their study is that they did not measure patients' preference as specifically as in our analyses, since they also included a minority of 2-stage trials, as physician preference.

For peer review only - http://bmjopen.bmj.com/site/about/guidelines.xhtml

An RCT is once designed to reliably compare medication to placebo.[71] In the hierarchy of research designs, the results of RCTs are considered to be evidence of the highest grade. Lessons learned from the history of RCT, early studies from 1970 and 1980s suggested that observational studies suffer too much from confounders and frequently result in overestimation of treatment effects compared with RCTs.[72,73] Consequently, many experts advocated that results of observational studies should not be used for defining evidence-based medical care: *''If the study wasn't randomized, we suggest that you stop reading it and go on to the next article''*.[74] However, two updates of this work including studies between 1985 and 1995 found little evidence that estimates of treatment effects in observational studies are consistently larger from those obtained in RCTs.[75,76] It is suggested that observational studies have methodologically improved over time with the use of a control group, carefully defining in- and exclusion criteria, and by better understanding confounders. The fundamental criticism of the CCT could be that within the preference cohort the unrecognized confounding factors may distort the results. Yet, our results showed that preference cohorts provide valid information comparable with the randomised results.

Today, the classic levels of evidence are subject of debate, as the disadvantages of RCTs have become more insightful in modern practice. In general, patients participating in RCTs are highly selected. Less than 10% of patients participate in trials, partly due to exclusion of patients with a specific treatment preference.[77] This limits the extrapolation of RCT results to patients seen in routine practice. Another consequence is that the majority of trials takes several years to be completed. This not only causes a burden on health research costs, but also results in a questionable ethical dilemma. Developments are fast and the relevance of trials may therefore change over time. Consequently, if an RCT is optimally designed but takes too long, the results will be outdated.

This especially applies, when designing a trial in which it can be foreseen that patients' preference will be a prominent factor. For example in trials comparing treatments of significant different nature (medical versus surgical). Anticipation on the expected patients' preference by eliminating this factor is at the expense of the validity of a lot of RCTs. Especially when patient-centred outcomes are used, one should consider whether the most important patients group has been excluded. Trials must be internally valid, but lack of consideration of external validity causes the widespread underuse of

For peer review only

treatments -that showed superiority in RCTs- in routine practice. Moreover, in these situations a CCT could be the superior design over an RCT.

CCTs provide unique data on external and internal validity as the patients in the preference cohort are followed according the same conditions as the patients in the randomised cohorts. A limitation of our review is that interventions and settings between CCTs were very diverse. On the other hand, because of this diversity, it could also be stated that randomised data and preference data often produce similar results; in all kind of settings. None of the primary outcomes of trials included in the meta-analyses were objective and thought to be 'non-influenceable' by a patient's experience of the treatment. Nevertheless, as it is supposed preference would more likely affect subjective outcomes, evaluating objective outcomes as e.g. mortality is of less interest. Concerning the assessment of external validity, it should be noted that in only a minority of trials the differences in sociodemographic and clinical parameters between the cohorts of a CCT were evident. Another limitation is that it was not possible to objectively establish the quality of included trials, as there is currently no valid critical appraisal tool to apply for a CCT. Consequently, our results may have been influenced by the inclusions of flawed trials.

In conclusion, CCTs seem to be a reliable alternative for RCTs, especially in trials comparing treatments of vastly difference nature (e.g. medical vs surgical) or using patient-centred outcomes. In case patients' preference can be assumed, CCT enables faster inclusion of a more representative population improving external validity without compromising internal validity.

For peer review only

**Author Contributors:** KW and CB design the study, KW and PW performed the search, KW and SvD did the statistical analyses, KW wrote the first draft with input of CB and WB.

**No competing interests:** All authors have completed the ICMJE uniform disclosure form at www.icmje.org/coi_disclosure.pdf and declare: no support from any organisation for the submitted work; no financial relationships with any organisations that might have an interest in the submitted work in the previous three years; no other relationships or activities that could appear to have influenced the submitted work.

**Acknowledgments:** None.

**Ethics approval**: Not applicable.

**Funding:** This research received no specific grant from any funding agency in the public, commercial or not-for-profit sectors.

**Data sharing:** Anonymised patient level data can be made available on reasonable request after approval from the trial management committee and after signing a data access agreement. Proposals should be directed to the corresponding author. Consent was not obtained for data sharing, but the presented data are anonymised and the risk of identification is low.

**Transparency:** The lead author (CB) affirms that the manuscript is an honest, accurate, and transparent account of the study being reported; that no important aspects of the study have been omitted; and that any discrepancies from the study as planned have been explained.

For peer review only

**Pubmed search strategy:**

5-10-2018

(patient preference design*[tiab] OR patient preference model*[tiab] OR patient preference trial*[tiab] OR patient preference method*[tiab] OR comprehensive cohort stud*[tiab] OR comprehensive cohort design*[tiab] OR patient preference group[tiab] OR patient preference allocation arms[tiab] OR preference allocation[tiab] OR randomized preference trial*[tiab] OR randomised preference trial*[tiab] OR preference arms[tiab] OR preferences[ti] OR treatment preference basis[tiab] OR (patient preference*[tiab] AND random*[ti]) OR (prefer*[ti] AND random*[ti]) OR (registry patient*[tiab] AND randomized[tiab])) AND ("Clinical Trial"[pt] OR trial[ti] OR preference trial[tiab]) AND ("2004/09"[Date - Publication] : "3000"[Date - Publication])

And

((patient preferences[ti] AND clinical trials[ti]) OR nonrandomized[ti] OR (patient preference[ti] AND randomization[ti]) OR (random[ti] AND nonrandom assignment[ti]) OR (randomized[ti] AND non-randomized[ti]) OR (nonrandom assignment[ti]) OR (randomized[ti] AND nonrandomized[ti]) OR (randomi*[tiab] AND preference arm) OR  (partially randomized study[tiab] AND "Randomized Controlled Trial"[pt]) OR (unwilling to be randomized[tiab] AND "Randomized Controlled Trial"[pt]) OR (choice[tiab] AND randomisation[tiab] AND "Randomized Controlled Trial"[pt]))  AND (random*[tiab]) AND ("Clinical Trial"[pt] OR trial[ti] OR clinical trials[ti])  AND ("2004/09"[Date - Publication] : "3000"[Date - Publication])

"comprehensive cohort*"[tiab]  AND ("2004/09"[Date - Publication] : "3000"[Date - Publication])

For peer review only

**Figure and Tables**

**Figure legends:**

**Figure 1.** Study selection according to PRISMA

**Figure 2.** A comprehensive cohort trial

**Figure 3.** Forest plot of the preference effect on the primary outcome by comparing the treatment effect of the experimental treatment over the control treatment (standardized effect size) of the randomised cohort versus the preference cohort.

**Figure 4.** Forest plot of the preference effect on the primary outcome of trials in which the primary outcome is adjusted for confounders by comparing the treatment effect of the experimental treatment over the control treatment (standardized effect size) of the randomised cohort versus the preference cohort.

**Figure 5**. Forest plot of the preference effect on the primary outcome of trials in which the primary outcome is not adjusted for confounders by comparing the treatment effect of the experimental treatment over the control treatment (standardized effect size) of the randomised cohort versus the preference cohort.

**Supplementary material**

**Supplement table 1**. Significant sociodemographic findings preference vs randomised cohorts

**Supplement 2.** Study protocol

**Supplement 3.** PRISMA checklist

For peer review only

**Table 1.** Comprehensive cohort trials included in the review

| Source | Population | No. R. | P. | Field | Intervention and comparison groups | Prim. Outcome(s) |
|---|---|---|---|---|---|---|
| Ashok et al,[12] 2005 | Woman presenting for termination of pregnancy | 400 | 86 | Gynaecology | Medical vs surgical termination^+ | Acceptability at 2 wk |
| Barnard et al,[13] 2016 | Premenopausal women with symptomatic uterine fibroids | 59 | 34 | Gynaecology | UAE vs MRgFUS^+ | Perioperative outcomes at 3 mo |
| Bergk, J. et al,[35] 2011 | Patients with DSM-IV disorder | 27 | 81 | Psychiatry | Mechanical restraint vs seclusion | CES at 4 wk |
| Boers et al,[46] 2017 | Pregnant women with disproportional intrauterine growth | 650 | 452 | Gynaecology | Induction vs expectative monitoring^ | (S)AE neonate at discharge |
| Brinkhaus et al,[57] 2017* | Patients with allergic asthma | 357 | 1088 | Social medicine | Acupuncture vs control^ | AQLQ at 3 mo |
| Brinkhaus et al,[66] 2008 | Patients with allergic rhinitis | 981 | 4256 | Social medicine | Acupuncture vs control^ | RQLQ at 30 d |
| Buhagiar et al, [67] 2017* | Patients after total knee arthroplasty | 165 | 87 | Orthopaedics | Home based vs inpatients recovery | Walking distance at 36 wk |
| Chekerov et al,[68] 2017 | Elderly with ovarian cancer receiving chemotherapy | 3 | 116 | Gynaecology | oral vs iv treosulfan | DFS at 2 y |
| Creutzig et al,[69] 2014 | Paediatric patients with relapsed AML | 101 | 54 | Haematology | L-DNR/Flag vs Flag | OS at 4 y |
| Crowther et al,[14] 2012 | Pregnant women with one prior caesarean | 22 | 2323 | Gynaecology | Caesarean vs vaginal birth^+ | Death and SAE at 30 d |
| Dalal et al,[15] 2006* | Participants in cardiac rehabilitation after acute MI | 104 | 126 | Cardiology | Home based vs hospital recovery | HAD at 9 mo |
| Ejlertsen et al,[16] 2008 | Premenopausal patients with breast cancer | 525 | 1628 | Oncology | Chemotherapy vs ovarian ablation^+ | DFS at 10 y |
| Erkan et al,[17] 2007 | Patients with positive aPL but no vascular and/or pregnancy events. | 98 | 74 | Internal medicine | Aspirin vs placebo or no aspirin^ | Acute thrombosis per 100-patients y |
| Fong et al,[18] 2015 | Patients with adolescent idiopathic scoliosis | 19 | 50 | Orthopedics | Brace vs observational^ | Recruitment feasibility |

For peer review only

| | | | | | | |
|---|---|---|---|---|---|---|
| Gall et al,[19] 2007 | Patients undergoing colon cancer surgery | 203 | 135 | Surgery | GP – vs surgeon follow up | PCS score at 24 mo |
| Glazener et al,[20] 2016 | Patients with vaginal wall prolapse | 1348 | 1126 | Gynaecology | Mesh vs no mesh^+ | POPSS at 12 mo |
| Grant et al,[21] 2008* | Patients with gastro-oesophageal reflux disease | 357 | 453 | Upper GI | Surgery vs medication^+ | Reflux QOL at 1 y |
| Hatcher et al,[23] 2005 | Patients presenting with self-harm | 552 | 542 | Psychiatry | PST plus standard care vs standard care^ | Repeated self-harm at 1 y |
| Howard et al,[25] 2010* | Women requiring voluntary psychiatric admission | 42 | 61 | Psychiatry | crisis houses vs psychiatric wards | Functioning (GAF) at 12 wk |
| Hubacher et al,[26] 2017* | Women 18-29 years who were seeking a short -acting method | 382 | 524 | Gynaecology | long-acting vs short-acting contraceptive^ | Continuation rate at 1 y |
| Jones et al,[27] 2011* | Palliative cancer patients | 41 | 36 | Oncology | advance vs usual care^ | VAS (S) at 8 wk |
| Karlsen et al,[29] 2007 | Patients with proximal ureter stones | 50 | 21 | Urology | Shock wave vs ureteroscopy^+ | Stone free rate at 3 mo |
| Kearney et all,[30] 2011 | Patients with an acute Achilles tendon rupture | 20 | 29 | orthopedics | Surgery vs conservative^+ | Disability rating index at 9 mo |
| Kroz et all,[31] 2017 | Patients with breast cancer -related fatigue | 65 | 61 | Oncology | Multimodel combined program vs aerobic training^ | PSQI at 10 wk |
| Lock et al,[32] 2010 | Children with recurrent sore throats | 268 | 461 | Children Surgery | Surgery vs medication^+ | No. episodes of sore throats at 2 y |
| Majumdar et al,[33] 2010* | Patients with lower urinary tract symptoms (LUTS) | 99 | 210 | Urology | Urodynamics vs conservative^+ | Kings QOL at 6 mo |
| Mittal et al,[37] 2017* | Patients with type B ankle fracture | 160 | 276 | Orthopedics | Surgery vs no surgery^+ | FAOQ and PCI at 12 mo |
| Prescott et al,[40] 2007 | Women after breast conserving surgery | 255 | 100 | Oncology | Non- vs radiotherapy^ | QoL after 5 y |
| Purepong et al,[41] 2015* | Office workers suffering from low back pain (LBP) | 64 | 37 | Physical therapy | Backrest vs no intervention^ | VAS at 3 mo |
| Raue et al,[43] 2011 | Patients operated for diverticulitis | 149 | 294 | Surgery | Laparoscopic vs open approach | QoL at 30 d |
| Robson et al,[44] 2009* | Termination of pregnancy less than 14 weeks gestation | 349 | 1528 | Gynaecology | Medicine vs surgery TOP^+ | Acceptability TOP at 2 wk |

For peer review only

| | | | | | | |
|---|---|---|---|---|---|---|
| Schweikert et al,[47] 2009 | Patient for cardiac rehabilitation | 4 | 163 | Cardiology | Out-patient vs in-patient recovery | EQ-5D at 12 mo |
| Shi guang et al,[50] 2014* | Patients with vascular dementia | 48 | 20 | Alternative medicine | Acupuncture vs training^ | SDSVD at |
| Sinclair et al,[51] 2017* | Patients with severe lung disease | 67 | 82 | Pulmonology | Advance care planning vs standard | ACP uptake at 6 mo |
| Schwieger et al,[48] 2016* | Adolescent with idiopathic scoliosis (AIS) | 132 | 187 | Orthopaedics | Brace vs observation^ | QOL at 2 y |
| Underwood et al,[52] 2008* | Older patients with chronic knee pain | 282 | 303 | Orthopaedics | Topic vs oral ibuprofen | WOMAC at 12 mo |
| van der Kooij et al,[54] 2013 | Uterine fibroids | 177 | 103 | Gynaecology | Embolization vs hysterectomy^+ | HRQoL at 12 mo |
| Van Heest et al,[55] 2015 | Children with upper extremity cerebral palsy | 29 | 10 | Orthopedics | Surgery vs botuline therapy^+ | SHUEE at 24 wk |
| Weinstein et al,[58] 2006* | Patients with spondylolisthesis | 304 | 303 | Orthopaedics | Surgical vs non-surgical^+ | Physical functioning (SF-36 Phys) at 2 y |
| Weinstein et al,[56] 2008* | Patients with spinal stenosis | 289 | 365 | Orthopaedics | Surgical vs non-surgical^+ | Physical functioning (SF-36 Phys) at 2 y |
| Witbrodt,[60] 2007* | addicted people | 293 | 321 | Social medicine | Community residential vs day hospital^ | Abstinence at 12 mo |
| Witt el al,[61] 2006* | Patients with chronic low back pain | 2841 | 8537 | Rheumatology | Acupuncture vs control^ | HFAQ at 3 mo |
| Witt et al,[62] 2006* | Patients with osteoarthritis | 712 | 2921 | Rheumatology | Acupuncture vs control^ | Osteoarthritis index (WOMAC) at 3 mo |
| Woodward et al,[65] 2004 | Pregnant women | 60 | 20 | Gynaecology | Water- vs land birth | Baby condition at 6 wk |

*These 20 trials could be used to calculate standardised effect sizes of the randomised- and preference cohort separately, and were included in our reanalysis on the effect of preference on outcome. ^These 32 trials compared interventions versus conservative treatment. +These 16 trials compared surgical interventions versus conservative treatment.

Abbreviations: Wk, week; mo, months; y, year; MRgFUS, magnetic resonance imaging-guided focused ultrasound surgery; UAE, uterine artery embolization; HRQoL, Health related Quality of Life; CES, Coercion Experience Scale; WOMAC, Western Ontario and McMaster Universities Osteoarthritis Index; HFAQ, Hannover Functional Ability Questionnaire; AQLQ, Astma Quality of Life; SAE, Serious adverse event; HAD, Hospital Anxiety Depression scale; GAF, Global assessment of functioning; BPRS, Brief psychiatric rating scale; VAS, Visual analogue scales; FAOQ, Foot and Ankle outcomes questionnaire; PCI, Physical component score; RMDQ, Roland-Morris Disability Questionnaire; TOP, Termination of pregnancy; SVSVD, Scale of differentiation of syndromes of vascular dementia; ACP, Advance care planning; DFS, disease free survival; OS, overall survival; PCS, peritoneal cancer score; PST, problem solving therapy; RQLQ,

For peer review only - http://bmjopen.bmj.com/site/about/guidelines.xhtml

Rhinitis Quality of life questionnaire; L-DNR, liposomal daunorubicin; FLAG, fludarabut; POPSS, Pelvic organ prolapse symptom score; SHUEE, Shriners Hospital Upper Extremity Evaluation; SF-36 Phys, short-from 36 scale physical functioning; PSQI, Pittsburg sleep Quality index; R, randomised; P, preference.

For peer review only

## References

1 Effectiveness. NC for R and DU systematic reviews of research on. Centre for Reviews and Disseminationt's Guidance for Those Carrying Out or Commissioning Reveiws. *York, Engl Univ York* 2001;**Report 4**.

2 Brewin CR, Bradley C. Patient preferences and randomised clinical trials. *Br Med J* 1989;**299**:313–5. doi:10.1136/bmj.299.6694.313

3 King M, Nazareth I, Lampe F, *et al.* Impact of Participant and Physician Intervention Preferences on Randomized Trials. *JAMA* 2005;**293**:1089. doi:10.1001/jama.293.9.1089

4 Higgins JPT GS (Eds. . Cochrane handbook for systematic reviews of interventions. Version 5.1.0. *Cochrane Collab Oxford;* Published Online First: 2011.http://handbook-5-1.cochrane.org/

5 Moher D, Liberati A, Tetzlaff J, *et al.* Preferred reporting items for systematic reviews and meta-analyses: the PRISMA statement. *BMJ* 2009;**339**:b2535–b2535. doi:10.1136/bmj.b2535

6 Sterne JA, Hernán MA, Reeves BC, *et al.* ROBINS-I: a tool for assessing risk of bias in non-randomised studies of interventions. *BMJ* 2016;**355**:i4919. doi:10.1136/bmj.i4919

7 Higgins JPT, Altman DG, Gotzsche PC, *et al.* The Cochrane Collaboration's tool for assessing risk of bias in randomised trials. *BMJ* 2011;**343**:d5928–d5928. doi:10.1136/bmj.d5928

8 Guyatt GH, Ebrahim S, Alonso-Coello P, *et al.* GRADE guidelines 17: assessing the risk of bias associated with missing participant outcome data in a body of evidence. *J Clin Epidemiol* 2017;**87**:14–22. doi:10.1016/j.jclinepi.2017.05.005

9 Wan X, Wang W, Liu J, *et al.* Estimating the sample mean and standard deviation from the sample size, median, range and/or interquartile range. *BMC Med Res Methodol* 2014;**14**:135. doi:10.1186/1471-2288-14-135

10 MW Lipsey DW. Practical meta-analysis. *Thousan Oaks, Calif Sage Publ* 2001.

11 16.1.3.2 Imputing standard deviations for changes from baseline. http://handbook-5-1.cochrane.org/chapter_16/16_1_3_2_imputing_standard_deviations_for_changes_from_baseline.htm (accessed 10 Jul 2018).

12 Ashok PW, Hamoda H, Flett GM, *et al.* Patient preference in a randomized study comparing medical and surgical abortion at 10-13 weeks gestation. *Contraception* 2005;**71**:143–8. doi:10.1016/j.contraception.2004.08.013

13 Barnard EP, AbdElmagied AM, Vaughan LE, *et al.* Periprocedural outcomes comparing fibroid embolization and focused ultrasound: a randomized controlled trial and comprehensive cohort analysis. *Am J Obstet Gynecol* 2017;**216**:500.e1-500.e11. doi:10.1016/j.ajog.2016.12.177

14 Crowther CA, Dodd JM, Hiller JE, *et al.* Planned Vaginal Birth or Elective Repeat Caesarean: Patient Preference Restricted Cohort with Nested Randomised Trial. *PLoS Med* 2012;**9**:e1001192. doi:10.1371/journal.pmed.1001192

15 Dalal HM, Evans PH, Campbell JL, *et al.* Home-based versus hospital-based rehabilitation after myocardial infarction: A randomized trial with preference arms--Cornwall Heart Attack Rehabilitation Management Study (CHARMS). *Int J Cardiol* 2007;**119**:202–11. doi:10.1016/j.ijcard.2006.11.018

16 Ejlertsen B, Jensen M-B, Mouridsen HT, *et al.* DBCG trial 89B comparing adjuvant CMF and ovarian ablation: similar outcome for eligible but non-enrolled and randomized breast cancer

For peer review only

patients. *Acta Oncol* 2008;**47**:709–17. doi:10.1080/02841860802001475

17 Erkan D, Harrison MJ, Levy R, *et al.* Aspirin for primary thrombosis prevention in the antiphospholipid syndrome: a randomized, double-blind, placebo-controlled trial in asymptomatic antiphospholipid antibody-positive individuals. *Arthritis Rheum* 2007;**56**:2382–91.http://search.ebscohost.com/login.aspx?direct=true&db=rzh&AN=105979335&lang=nl&site=ehost-live&scope=site

18 Fong DYT, Cheung KMC, Wong YW, *et al.* An alternative to a randomised control design for assessing the efficacy and effectiveness of bracing in adolescent idiopathic scoliosis. *Bone Joint J* 2015;**97**–**B**:973–81. doi:10.1302/0301-620X.97B7.35147

19 Gall CA, Weller D, Esterman A, *et al.* Patient satisfaction and health-related quality of life after treatment for colon cancer. *Dis Colon Rectum* 2007;**50**:801–9. doi:10.1007/s10350-006-0815-8

20 Glazener C, Breeman S, Elders A, *et al.* Clinical effectiveness and cost-effectiveness of surgical options for the management of anterior and/or posterior vaginal wall prolapse: two randomised controlled trials within a comprehensive cohort study – results from the PROSPECT Study. *Health Technol Assess (Rockv)* 2016;**20**:1–452. doi:10.3310/hta20950

21 Grant A, Boachie C, Cotton S, *et al.* Clinical and economic evaluation of laparoscopic surgery compared with medical management for gastro-oesophageal reflux disease: 5-year follow-up of multicentre randomised trial (the REFLUX trial). *Heal Technol Assess* 2013;**17**:1–167. doi:10.3310/hta17220

22 Hartley S, Haddock G. Self-help therapy and recovery in psychosis: methodological considerations and service user involvement in a partially randomised preference trial. *Trials* 2011;**12**:A84. doi:10.1186/1745-6215-12-S1-A84

23 Hatcher S, Sharon C, Parag V, *et al.* Problem-solving therapy for people who present to hospital with self-harm: Zelen randomised controlled trial. *Br J Psychiatry* 2011;**199**:310–6. doi:10.1192/bjp.bp.110.090126

24 Barnestein-Fonseca P, Vazquez-Alarcon R, Leiva-Fernandez F, *et al.* Inhalation Technique Evolution After Training in Copd. The Role of the Device. *Value Heal* 2014;**17**:A600. doi:10.1016/j.jval.2014.08.2076

25 Howard L, Flach C, Leese M, *et al.* Effectiveness and cost-effectiveness of admissions to women's crisis houses compared with traditional psychiatric wards: pilot patient-preference randomised controlled trial. *Br J Psychiatry Suppl* 2010;**53**:s32-40. doi:10.1192/bjp.bp.110.081083

26 Hubacher D, Spector H, Monteith C, *et al.* Long-acting reversible contraceptive acceptability and unintended pregnancy among women presenting for short-acting methods: a randomized patient preference trial. *Am J Obstet Gynecol* 2017;**216**:101–9. doi:10.1016/j.ajog.2016.08.033

27 Jones L, Harrington J, Barlow CA, *et al.* Advance care planning in advanced cancer: can it be achieved? An exploratory randomized patient preference trial of a care planning discussion. *Palliat Support Care* 2011;**9**:3–13. doi:10.1017/S1478951510000490

28 Karidakis GK, Karachalios T. Oxidized Zirconium Head on Crosslinked Polyethylene Liner in Total Hip Arthroplasty: A 7- to 12-year In Vivo Comparative Wear Study. *Clin Orthop Relat Res* 2015;**473**:3836–45. doi:10.1007/s11999-015-4503-7

29 Karlsen SJ, Renkel J, Tahir AR, *et al.* Extracorporeal shockwave lithotripsy versus ureteroscopy for 5- to 10-mm stones in the proximal ureter: Prospective effectiveness patient-preference trial. *J Endourol* 2007;**21**:28–33. doi:10.1089/end.2006.0153

30 Kearney RS, Achten J, Parsons NR, *et al.* The comprehensive cohort model in a pilot trial in orthopaedic trauma. *BMC Med Res Methodol* 2011;**11**:39. doi:10.1186/1471-2288-11-39

For peer review only

31 Kröz M, Reif M, Glinz A, *et al.* Impact of a combined multimodal-aerobic and multimodal intervention compared to standard aerobic treatment in breast cancer survivors with chronic cancer-related fatigue - results of a three-armed pragmatic trial in a comprehensive cohort design. *BMC Cancer* 2017;**17**:166. doi:10.1186/s12885-017-3142-7

32 Lock C, Wilson, J, Steen N, *et al.* Comparison of case note review methods for evaluating quality and safety in health care. *Health Technol Assess (Rockv)* 2010;**14**:1–164, iii–iv. doi:10.3310/hta14130

33 Majumdar A, Latthe P, Toozs-Hobson P. Urodynamics prior to treatment as an intervention: A pilot study. *Neurourol Urodyn* 2009;:n/a-n/a. doi:10.1002/nau.20810

34 Mills N, Metcalfe C, Ronsmans C, *et al.* A comparison of socio-demographic and psychological factors between patients consenting to randomisation and those selecting treatment (the ProtecT study). *Contemp Clin Trials* 2006;**27**:413–9. doi:10.1016/j.cct.2006.04.008

35 Bergk J, Einsiedler B, Flammer E, *et al.* A randomized controlled comparison of seclusion and mechanical restraint in inpatient settings. *Psychiatr Serv* 2011;**62**:1310–7. doi:10.1176/ps.62.11.pss6211_1310

36 Mitchell-Jones N, Farren JA, Tobias A, *et al.* Ambulatory versus inpatient management of severe nausea and vomiting of pregnancy: a randomised control trial with patient preference arm. *BMJ Open* 2017;**7**:e017566. doi:10.1136/bmjopen-2017-017566

37 Mittal R, Harris IA, Adie S, *et al.* Surgery for Type B Ankle Fracture Treatment: a Combined Randomised and Observational Study (CROSSBAT). *BMJ Open* 2017;**7**:e013298. doi:10.1136/bmjopen-2016-013298

38 Narasimmaraj P, Stover Fiscalini A, Kaplan C, *et al.* Abstract P3-10-01: A pilot feasibility study of the WISDOM study, a preference-tolerant randomized controlled trial evaluating a risk-based breast cancer screening strategy. *Cancer Res* 2016;**76**:P3-10-01-P3-10-01. doi:10.1158/1538-7445.SABCS15-P3-10-01

39 Nozaki I, Kato K, Igaki H, *et al.* Evaluation of safety profile of thoracoscopic esophagectomy for T1bN0M0 cancer using data from JCOG0502: a prospective multicenter study. *Surg Endosc* 2015;**29**:3519–26. doi:10.1007/s00464-015-4102-4

40 Prescott RJ, Kunkler IH, Williams LJ, *et al.* A randomised controlled trial of postoperative radiotherapy following breast-conserving surgery in a minimum-risk older population. The PRIME trial. *Health Technol Assess* 2007;**11**:1–149, iii–iv.http://www.ncbi.nlm.nih.gov/pubmed/17669280 (accessed 8 May 2018).

41 Purepong N, Channak S, Boonyong S, *et al.* The effect of an acupressure backrest on pain and disability in office workers with chronic low back pain: A randomized, controlled study and patients' preferences. *Complement Ther Med* 2015;**23**:347–55. doi:10.1016/j.ctim.2015.03.005

42 Ramanathan K, Ioannou K, Keshmiri H, *et al.* 326 Disparity Between Clinical Trials and Registry Outcomes: Reflections From the Bypass Angioplasty Revascularization Investigation 2 Diabetes (BARI-2D) Trial. *Can J Cardiol* 2012;**28**:S218–9. doi:10.1016/j.cjca.2012.07.307

43 Raue W, Langelotz C, Paolucci V, *et al.* Problems of randomization to open or laparoscopic sigmoidectomy for diverticular disease. *Int J Colorectal Dis* 2011;**26**:369–75. doi:10.1007/s00384-010-1074-7

44 Robson S, Kelly T, Howel, D, *et al.* Randomised preference trial of medical versus surgical termination of pregnancy less than 14 weeks' gestation (TOPS). *Health Technol Assess (Rockv)* 2009;**13**. doi:10.3310/hta13530

45 Ryan M, Nitsun M, Gilbert L, *et al.* A prospective study of the effectiveness of group and individual psychotherapy for women CSA survivors. *Psychol Psychother* 2005;**78**:465–79. doi:10.1348/147608305X42226

For peer review only

46 KE Boers, L van Wyk, JAM van der Post, A Kwee, MG van Pampus, HA Bremer, FMC Delemarre, KWM Bloemenkamp, S le Cessie, FME Roumen, JG Thornton, JMM van Lith, BW J Mol SS. *Comparison of participants and non-participnatns in a trial of induction of labour versus expectant monitoring for intrauterine growth restriction at term (teh DIGITAT trial); a prospective cohort study*. 2012.https://openaccess.leidenuniv.nl/bitstream/handle/1887/18948/06.pdf?sequence=12

47 Schweikert B, Hahmann H, Steinacker JM, *et al.* Intervention study shows outpatient cardiac rehabilitation to be economically at least as attractive as inpatient rehabilitation. *Clin Res Cardiol* 2009;**98**:787–95. doi:10.1007/s00392-009-0081-6

48 Schwieger T, Campo S, Weinstein SL, *et al.* Body Image and Quality-of-Life in Untreated Versus Brace-Treated Females With Adolescent Idiopathic Scoliosis. *Spine (Phila Pa 1976)* 2016;**41**:311–9. doi:10.1097/brs.0000000000001210

49 Shavelle D, Kapasi N, Banerjee S, *et al.* TCT-127 Prior Coronary Artery Bypass Graft Surgery and Hemodynamically Supported High Risk Percutaneous Coronary Intervention: Observations from The PROTECT II Randomized Trial and The cVAD Registry. *J Am Coll Cardiol* 2016;**68**:B51. doi:10.1016/j.jacc.2016.09.033

50 Shi GX, Liu CZ, Guan W, *et al.* Effects of acupuncture on Chinese medicine syndromes of vascular dementia. *Chin J Integr Med* 2014;**20**:661–6. doi:10.1007/s11655-013-1323-4

51 Sinclair C, Auret KA, Evans SF, *et al.* Advance care planning uptake among patients with severe lung disease: A randomised patient preference trial of a nurse-led, facilitated advance care planning intervention. *BMJ Open* 2017;**7**:e013415. doi:10.1136/bmjopen-2016-013415

52 Underwood M, Ashby D, Cross P, *et al.* Advice to use topical or oral ibuprofen for chronic knee pain in older people: randomised controlled trial and patient preference study. *BMJ* 2008;**336**:138–42. doi:10.1136/bmj.39399.656331.25

53 Impact of Vitamin D on Insulin Resistance... (PDF Download Available). https://www.researchgate.net/publication/264216208_Impact_of_Vitamin_D_on_Insulin_Resistance_in_Patients_with_Type_II_Diabetes_A_Comprehensive_Cohort_Design (accessed 8 May 2018).

54 van der Kooij SM, Hehenkamp WJK, Birnie E, *et al.* The effect of treatment preference and treatment allocation on patients' health-related quality of life in the randomized EMMY trial. *Eur J Obstet Gynecol Reprod Biol* 2013;**169**:69–74. doi:10.1016/j.ejogrb.2013.01.019

55 Van Heest AE, Bagley A, Molitor F, *et al.* Tendon transfer surgery in upper-extremity cerebral palsy is more effective than botulinum toxin injections or regular, ongoing therapy. *J Bone Jt Surg Am* 2015;**97**:529–36. doi:10.2106/jbjs.m.01577

56 Weinstein JN, Tosteson TD, Lurie JD, *et al.* Surgical versus Nonsurgical Therapy for Lumbar Spinal Stenosis. *N Engl J Med* 2008;**358**:794–810. doi:10.1056/NEJMoa0707136

57 Brinkhaus B, Roll S, Jena S, *et al.* Acupuncture in Patients with Allergic Asthma: A Randomized Pragmatic Trial. *J Altern Complement Med* 2017;**23**:268–77. doi:10.1089/acm.2016.0357

58 Weinstein JN, Lurie JD, Tosteson TD, *et al.* Surgical versus nonsurgical treatment for lumbar degenerative spondylolisthesis. *N Engl J Med* 2007;**356**:2257–70. doi:10.1056/NEJMoa070302

59 Wiegel T, Stöckle M, Bartkowiak D. PREFEREnce-based Randomized Evaluation of Treatment Modalities in Low or Early Intermediate-risk Prostate Cancer. *Eur Urol* 2015;**67**:1–2. doi:10.1016/j.eururo.2014.09.016

60 Witbrodt J, Bond J, Kaskutas LA, *et al.* Day hospital and residential addiction treatment: randomized and nonrandomized managed care clients. *J Consult Clin Psychol* 2007;**75**:947–59. doi:10.1037/0022-006x.75.6.947

For peer review only

61 Witt CM, Jena S, Selim D, *et al.* Pragmatic randomized trial evaluating the clinical and economic effectiveness of acupuncture for chronic low back pain. *Am J Epidemiol* 2006;**164**:487–96. doi:aje/kwj224

62 Witt CM, Jena S, Brinkhaus B, *et al.* Acupuncture in patients with osteoarthritis of the knee or hip: a randomized, controlled trial with an additional nonrandomized arm. *Arthritis Rheum* 2006;**54**:3485–93. doi:10.1002/art.22154

63 C.G. Wood, P. Srivastava, L. Lacombe, A.I. Gorelov, S. Gorelov, P. Mulders, H. Zielinski, F. Teofilovici, L. Isakov BE. Survival update from a multicenter, randomized, phase III trial of vitespen versus observation as adjuvant therapy for renal cell carcinoma in patients at high risk of recurrence. *J Clin Oncol* 2009;**27**:3009–3009. doi:10.1200/jco.2009.27.15s.3009 Journal of Clinical Oncology 27, no. 15S (May 20 2009) 3009-3009.

64 Yoneda T, Shoji K, Takase H, *et al.* Effectiveness and safety of 1-year ad libitum consumption of a high-catechin beverage under nutritional guidance. *Metab Syndr Relat Disord* 2009;**7**:349–56. doi:10.1089/met.2008.0061

65 Woodward J, Kelly SM. A pilot study for a randomised controlled trial of waterbirth versus land birth. *BJOG* 2004;**111**:537–45. doi:10.1111/j.1471-0528.2004.00132.x

66 Brinkhaus B, Witt CM, Jena S, *et al.* Acupuncture in patients with allergic rhinitis: a pragmatic randomized trial. *Ann Allergy, Asthma Immunol* 2008;**101**:535–43.http://search.ebscohost.com/login.aspx?direct=true&db=rzh&AN=105590802&lang=nl&site=ehost-live&scope=site

67 Buhagiar MA, Naylor JM, Harris IA, *et al.* Effect of Inpatient Rehabilitation vs a Monitored Home-Based Program on Mobility in Patients With Total Knee Arthroplasty: The HIHO Randomized Clinical Trial. *JAMA J Am Med Assoc* 2017;**317**:1037–46. doi:10.1001/jama.2017.1224

68 Chekerov R, Harter P, Fuxius S, *et al.* Preference of elderly patients' to oral or intravenous chemotherapy in heavily pre-treated recurrent ovarian cancer: final results of a prospective multicenter trial. *Gynecol Oncol Res Pract* 2017;**4**:6. doi:10.1186/s40661-017-0040-2

69 Creutzig U, Semmler J, Kaspers GL, *et al.* Re-induction with L-DNR/FLAG improves response after AML relapse, but not long-term survival. *Klin Padiatr* 2014;**226**:323–31. doi:10.1055/s-0034-1385918

70 Rothwell PM. External validity of randomised controlled trials: "To whom do the results of this trial apply?" *Lancet* 2005;**365**:82–93. doi:10.1016/S0140-6736(04)17670-8

71 Bothwell LE, Greene JA, Podolsky SH, *et al.* Assessing the Gold Standard — Lessons from the History of RCTs. *N Engl J Med* 2016;**374**:2175–81. doi:10.1056/NEJMms1604593

72 Chalmers TC, Celano P, Sacks HS, *et al.* Bias in treatment assignment in controlled clinical trials. *N Engl J Med* 1983;**309**:1358–61. doi:10.1056/NEJM198312013092204

73 Colditz GA, Miller JN, Mosteller F. How study design affects outcomes in comparisons of therapy. I: Medical. *Stat Med* 1989;**8**:441–54.http://www.ncbi.nlm.nih.gov/pubmed/2727468 (accessed 7 Aug 2018).

74 Sackett DL. *Evidence-based medicine : how to practice and teach EBM*. Churchill Livingstone 2000. https://books.google.nl/books?hl=nl&id=Qh1ntQEACAAJ&focus=searchwithinvolume&q=stop+reading (accessed 7 Aug 2018).

75 Benson K, Hartz AJ. A Comparison of Observational Studies and Randomized, Controlled Trials. *N Engl J Med* 2000;**342**:1878–86. doi:10.1056/NEJM200006223422506

76 Concato J, Shah N, Horwitz RI. Randomized, controlled trials, observational studies, and the

hierarchy of research designs. *N Engl J Med* 2000;**342**:1887–92. doi:10.1056/NEJM200006223422507

77 Murthy VH, Krumholz HM, Gross CP. Participation in Cancer Clinical Trials. *JAMA* 2004;**291**:2720. doi:10.1001/jama.291.22.2720

For peer review only

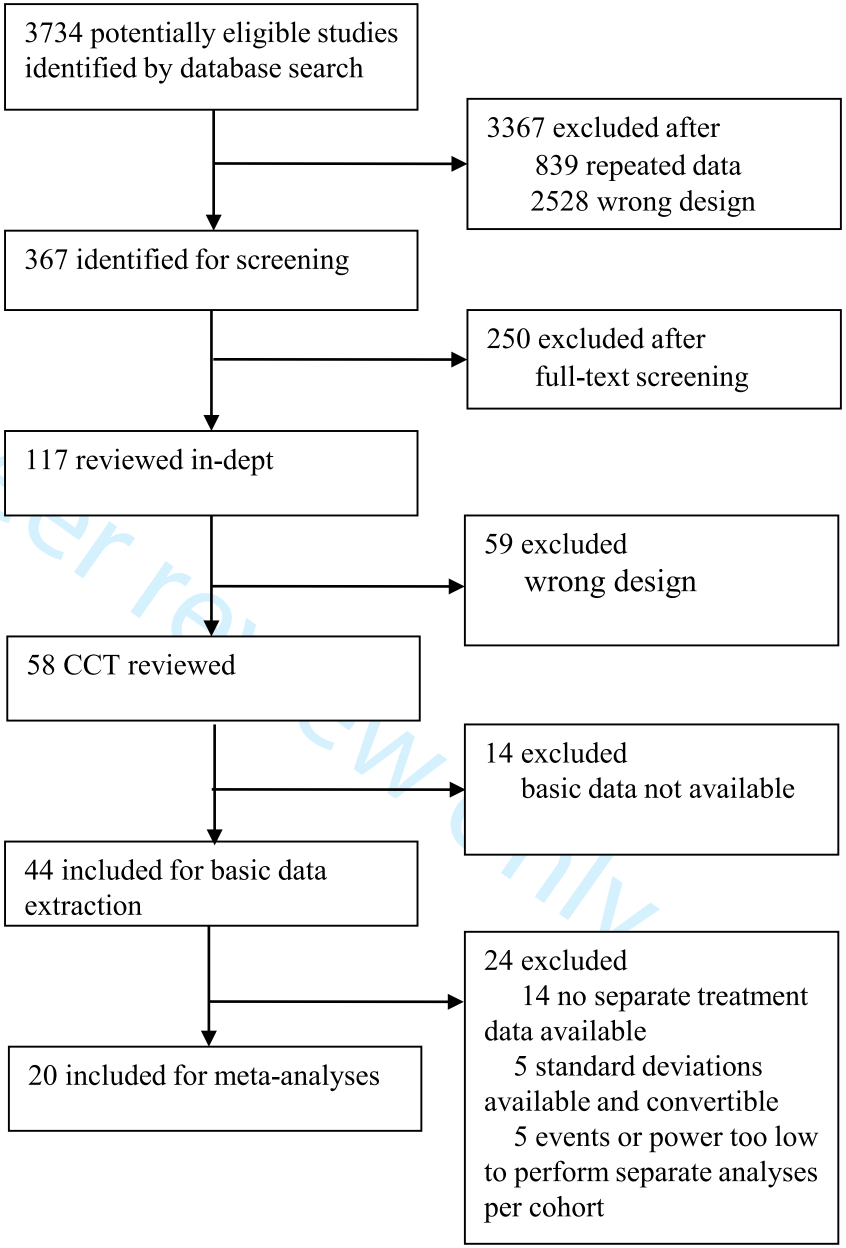


For peer review only

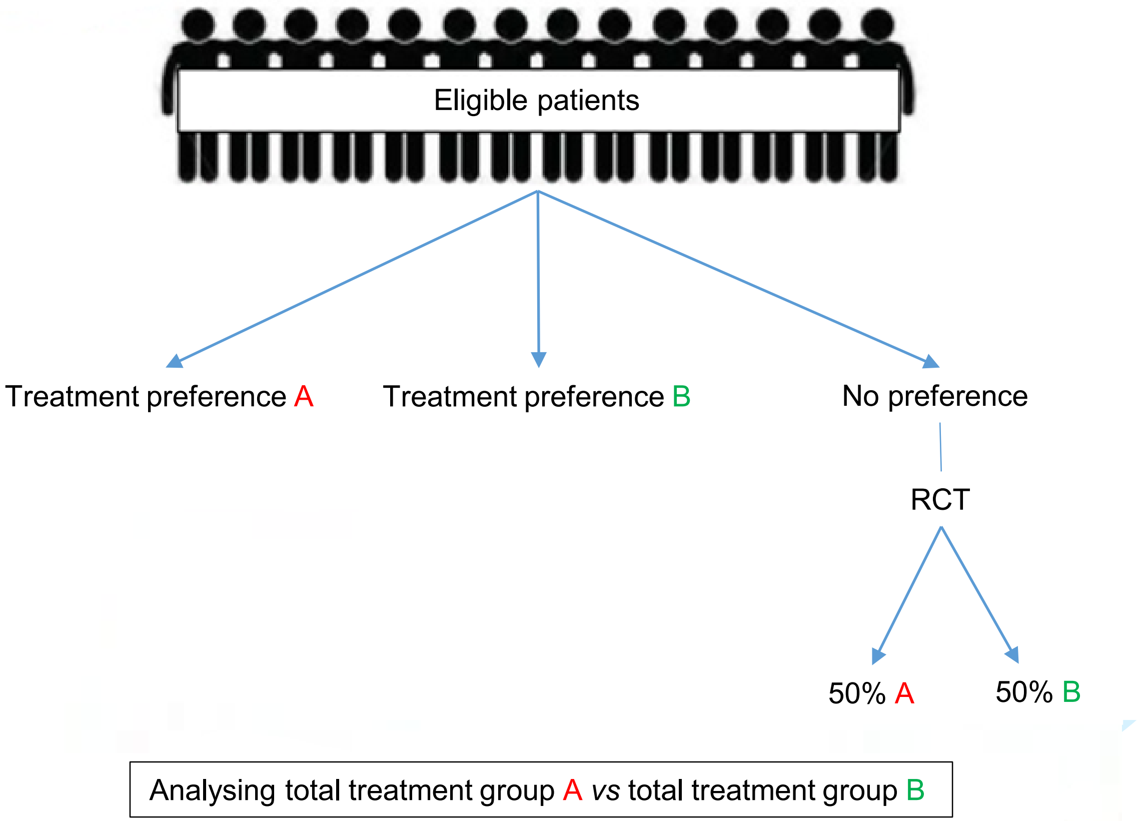
Eligible patients
Treatment preference A
Treatment preference B
No preference
RCT
50% A
50% B
Analysing total treatment group A vs total treatment group B

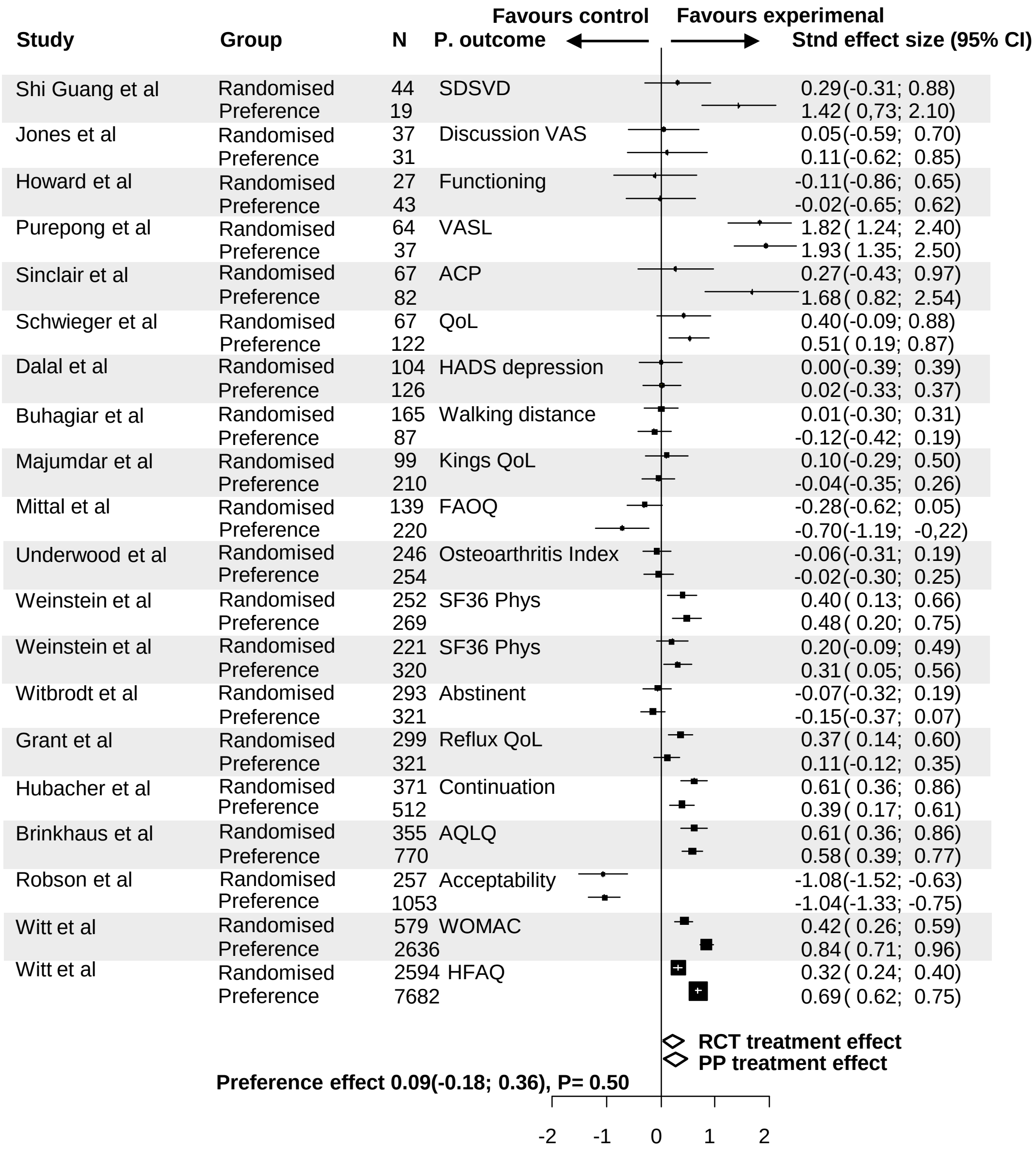
Favours control
Favours experimenal
Study
Group
N
P. outcome
Stnd effect size (95% CI)
Shi Guang et al Randomised 44 SDSVD 0.29(-0.31; 0.88)
Preference 19 1.42( 0,73; 2.10)
Jones et al Randomised 37 Discussion VAS 0.05(-0.59; 0.70)
Preference 31 0.11(-0.62; 0.85)
Howard et al Randomised 27 Functioning -0.11(-0.86; 0.65)
Preference 43 -0.02(-0.65; 0.62)
Purepong et al Randomised 64 VASL 1.82( 1.24; 2.40)
Preference 37 1.93( 1.35; 2.50)
Sinclair et al Randomised 67 ACP 0.27(-0.43; 0.97)
Preference 82 1.68( 0.82; 2.54)
Schwieger et al Randomised 67 QoL 0.40(-0.09; 0.88)
Preference 122 0.51( 0.19; 0.87)
Dalal et al Randomised 104 HADS depression 0.00(-0.39; 0.39)
Preference 126 0.02(-0.33; 0.37)
Buhagiar et al Randomised 165 Walking distance 0.01(-0.30; 0.31)
Preference 87 -0.12(-0.42; 0.19)
Majumdar et al Randomised 99 Kings QoL 0.10(-0.29; 0.50)
Preference 210 -0.04(-0.35; 0.26)
Mittal et al Randomised 139 FAOQ -0.28(-0.62; 0.05)
Preference 220 -0.70(-1.19; -0,22)
Underwood et al Randomised 246 Osteoarthritis Index -0.06(-0.31; 0.19)
Preference 254 -0.02(-0.30; 0.25)
Weinstein et al Randomised 252 SF36 Phys 0.40( 0.13; 0.66)
Preference 269 0.48( 0.20; 0.75)
Weinstein et al Randomised 221 SF36 Phys 0.20(-0.09; 0.49)
Preference 320 0.31( 0.05; 0.56)
Witbrodt et al Randomised 293 Abstinent -0.07(-0.32; 0.19)
Preference 321 -0.15(-0.37; 0.07)
Grant et al Randomised 299 Reflux QoL 0.37( 0.14; 0.60)
Preference 321 0.11(-0.12; 0.35)
Hubacher et al Randomised 371 Continuation 0.61( 0.36; 0.86)
Preference 512 0.39( 0.17; 0.61)
Brinkhaus et al Randomised 355 AQLQ 0.61( 0.36; 0.86)
Preference 770 0.58( 0.39; 0.77)
Robson et al Randomised 257 Acceptability -1.08(-1.52; -0.63)
Preference 1053 -1.04(-1.33; -0.75)
Witt et al Randomised 579 WOMAC 0.42( 0.26; 0.59)
Preference 2636 0.84( 0.71; 0.96)
Witt et al Randomised 2594 HFAQ 0.32( 0.24; 0.40)
Preference 7682 0.69( 0.62; 0.75)
RCT treatment effect
PP treatment effect
Preference effect 0.09(-0.18; 0.36), P= 0.50
-2
-1
0
1
2

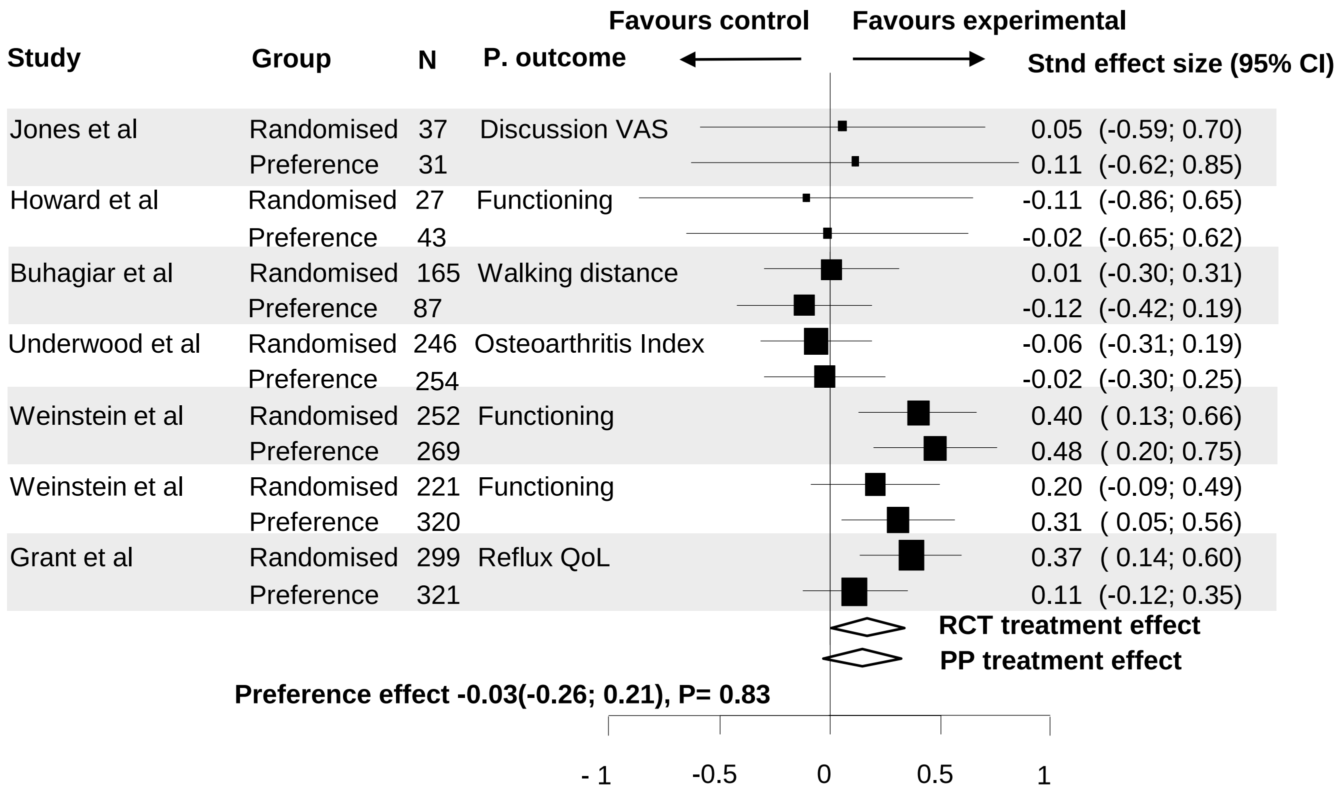
Favours control
Favours experimental
Study
Group
N
P. outcome
Stnd effect size (95% CI)
Jones et al
Randomised
37
Discussion VAS
0.05 (-0.59; 0.70)
Preference
31
0.11 (-0.62; 0.85)
Howard et al
Randomised
27
Functioning
-0.11 (-0.86; 0.65)
Preference
43
-0.02 (-0.65; 0.62)
Buhagiar et al
Randomised
165
Walking distance
0.01 (-0.30; 0.31)
Preference
87
-0.12 (-0.42; 0.19)
Underwood et al
Randomised
246
Osteoarthritis Index
-0.06 (-0.31; 0.19)
Preference
254
-0.02 (-0.30; 0.25)
Weinstein et al
Randomised
252
Functioning
0.40 ( 0.13; 0.66)
Preference
269
0.48 ( 0.20; 0.75)
Weinstein et al
Randomised
221
Functioning
0.20 (-0.09; 0.49)
Preference
320
0.31 ( 0.05; 0.56)
Grant et al
Randomised
299
Reflux QoL
0.37 ( 0.14; 0.60)
Preference
321
0.11 (-0.12; 0.35)
RCT treatment effect
PP treatment effect
Preference effect -0.03(-0.26; 0.21), P= 0.83
- 1
-0.5
0
0.5
1

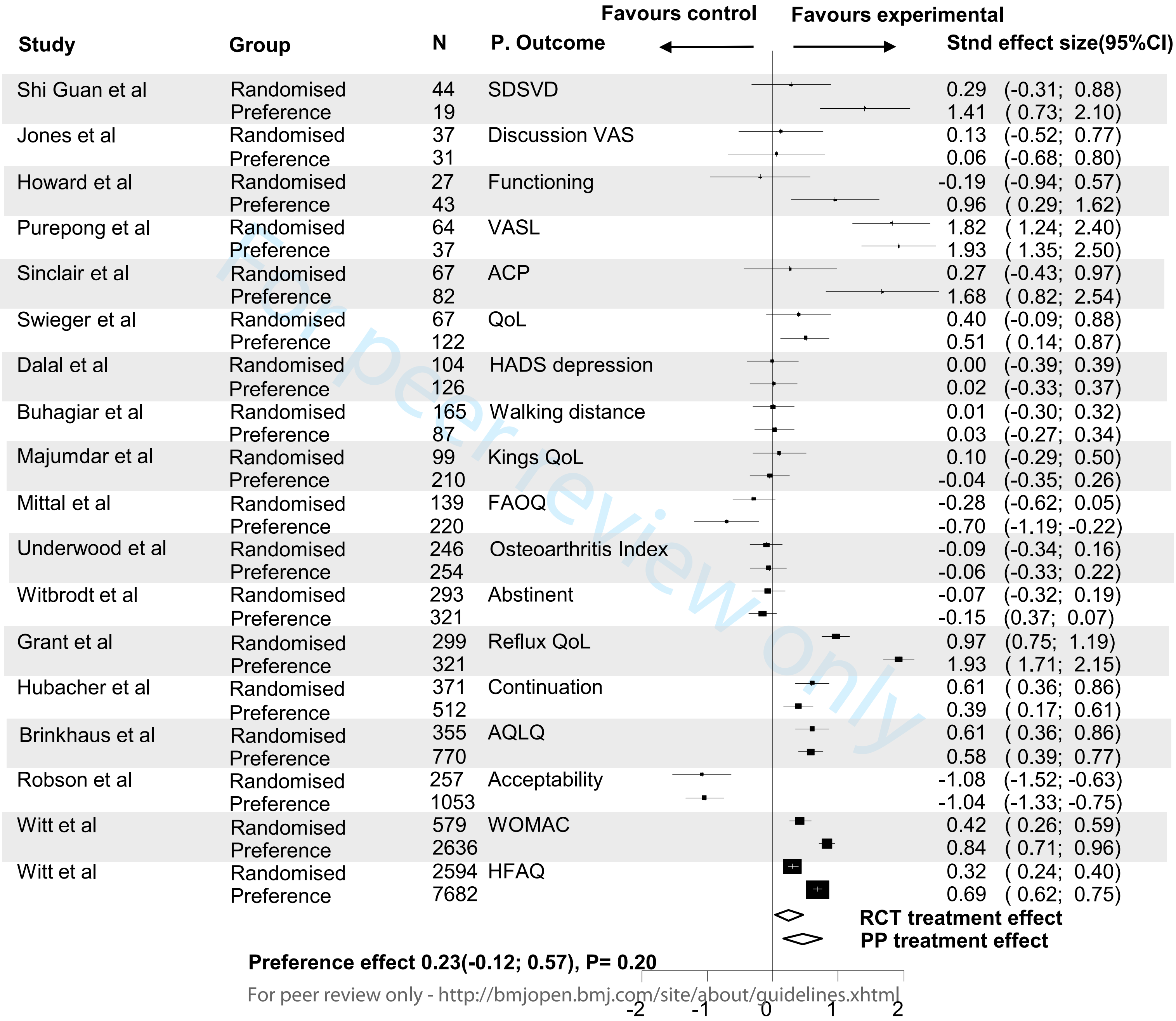


For peer review only

**Supplementary Table 1.** Significant sociodemographic findings preference vs randomised cohorts

| Preference cohorts in comparison to randomised cohorts | | |
|---|---|---|
| *Sociodemographic differences* | | |
| **Age** | Older[17,27,41,44,52,60] | 6/34 trials tested |
| | Younger[46,50] | 2/34 |
| **Gender** | Female[35,50] | 2/24 trials tested |
| | Male[67] | 1/24 |
| **Education** | Higher[17,46,51,61] | 4/19 trials tested |
| | Lower | 0/19 |
| **Employment** | Yes[14,18,26] | 3/13 trials tested |
| | No[52] | 1/13 trials tested |
| **Race** | Caucasian[14,17,54,56] | 4/14 trials tested |
| | Non-Caucasian[23] | 1/14 |
| **Obese** | Yes | 0/7 trials tested |
| | No[13,41,43,46] | 4/7 |
| **Smoking** | Yes | 0/5 trials tested |
| | No[13,46] | 2/5 |
| **Married** | Yes | 0/9 trials tested |
| | No[51] | 1/9 |
| **Experienced** | Yes[27,52,65] | 3/9 trials tested |
| | No[26] | 1/9 |
| *Clinical differences* | | |
| **Clinical problems** | More severe[13,21,23,26,37,54,60] | 7/20 trials tested |
| | Less severe[14,16,25,32,41,50,51,56,57,61] | 10/20 |
| | Not consistent[40,43,67] | 3/20 |

For peer review only

PROSPERO
International prospective register of systematic reviews

National Institute for Health Research

University of York
Centre for Reviews and Dissemination

# Systematic review

## 1. * Review title.

Give the working title of the review, for example the one used for obtaining funding. Ideally the title should state succinctly the interventions or exposures being reviewed and the associated health or social problems. Where appropriate, the title should use the PI(E)COS structure to contain information on the Participants, Intervention (or Exposure) and Comparison groups, the Outcomes to be measured and Study designs to be included.

Influence of patients' preference in randomised controlled trials

## 2. Original language title.

For reviews in languages other than English, this field should be used to enter the title in the language of the review. This will be displayed together with the English language title.

## 3. * Anticipated or actual start date.

Give the date when the systematic review commenced, or is expected to commence.
01/02/2017

## 4. * Anticipated completion date.

Give the date by which the review is expected to be completed.
12/03/2019

## 5. * Stage of review at time of this submission.

Indicate the stage of progress of the review by ticking the relevant Started and Completed boxes. Additional information may be added in the free text box provided.
Please note: Reviews that have progressed beyond the point of completing data extraction at the time of initial registration are not eligible for inclusion in PROSPERO. Should evidence of incorrect status and/or completion date being supplied at the time of submission come to light, the content of the PROSPERO record will be removed leaving only the title and named contact details and a statement that inaccuracies in the stage of the review date had been identified.
This field should be updated when any amendments are made to a published record and on completion and publication of the review. If this field was pre-populated from the initial screening questions then you are not able to edit it until the record is published.

The review has not yet started: Yes

For peer review only

**PROSPERO**
**International prospective register of systematic reviews**

National Institute for Health Research

| Review stage | Started | Completed |
|---|---|---|
| Preliminary searches | No | No |
| Piloting of the study selection process | No | No |
| Formal screening of search results against eligibility criteria | No | No |
| Data extraction | No | No |
| Risk of bias (quality) assessment | No | No |
| Data analysis | No | No |

Provide any other relevant information about the stage of the review here (e.g. Funded proposal, protocol not yet finalised).

## 6. * Named contact.

The named contact acts as the guarantor for the accuracy of the information presented in the register record.

Karin Wasmann

## Email salutation (e.g. "Dr Smith" or "Joanne") for correspondence:

Miss Wasmann

## 7. * Named contact email.

Give the electronic mail address of the named contact.

k.a.wasmann@amc.nl

## 8. Named contact address

Give the full postal address for the named contact.

Amsterdam UMC, department of surgery, Meibergdreef 9, 1105 AZ Amsterdam

## 9. Named contact phone number.

Give the telephone number for the named contact, including international dialling code.

00316-57066120

## 10. * Organisational affiliation of the review.

Full title of the organisational affiliations for this review and website address if available. This field may be completed as 'None' if the review is not affiliated to any organisation.

Amsterdam UMC

## Organisation web address:

## 11. * Review team members and their organisational affiliations.

Give the title, first name, last name and the organisational affiliations of each member of the review team. Affiliation refers to groups or organisations to which review team members belong.

Miss Karin Wasmann. Amsterdam UMC

For peer review only

**PROSPERO**
**International prospective register of systematic reviews**

NHS National Institute for Health Research

## 12. * Funding sources/sponsors.

Give details of the individuals, organizations, groups or other legal entities who take responsibility for initiating, managing, sponsoring and/or financing the review. Include any unique identification numbers assigned to the review by the individuals or bodies listed.

None

## 13. * Conflicts of interest.

List any conditions that could lead to actual or perceived undue influence on judgements concerning the main topic investigated in the review.

None

## 14. Collaborators.

Give the name and affiliation of any individuals or organisations who are working on the review but who are not listed as review team members.

## 15. * Review question.

State the question(s) to be addressed by the review, clearly and precisely. Review questions may be specific or broad. It may be appropriate to break very broad questions down into a series of related more specific questions. Questions may be framed or refined using PI(E)COS where relevant.

Influence of patients' preference in randomised controlled trials.

1) Patients’ preference will negatively influence participation to RCTs, decreasing external validity.

Therefore, the external validity of a patient preference trial (PPT) will be higher.

2) Patients’ preferences will influence outcomes in unblinded RCTs, decreasing internal validity. By using a PPT, patients with a preference will be included in the preference cohort and the remaining indifferent patients will be included in the RCT cohort, providing insight in the internal validity.

## 16. * Searches.

Give details of the sources to be searched, search dates (from and to), and any restrictions (e.g. language or publication period). The full search strategy is not required, but may be supplied as a link or attachment.

A systematic review including meta-analyses of PPTs was conducted. A search in PubMed, Embase, PsycINFO, and the Cochrane Library for PPTs published between Jan 1, 2005 and Oct 5, 2018 was executed without language restriction. The subject in the search strategy was PPT and possible aliases of PPT.

## 17. URL to search strategy.

Give a link to a published pdf/word document detailing either the search strategy or an example of a search strategy for a specific database if available (including the keywords that will be used in the search strategies), or upload your search strategy.Do NOT provide links to your search results.

https://www.crd.york.ac.uk/PROSPEROFILES/94438_STRATEGY_20190109.pdf

Alternatively, upload your search strategy to CRD in pdf format. Please note that by doing so you are consenting to the file being made publicly accessible.

Do not make this file publicly available until the review is complete

For peer review only

## 18. * Condition or domain being studied.

Give a short description of the disease, condition or healthcare domain being studied. This could include health and wellbeing outcomes.

Patient preference trials initiated for patients with any condition.

## 19. * Participants/population.

Give summary criteria for the participants or populations being studied by the review. The preferred format includes details of both inclusion and exclusion criteria.

Clinical trial patients who were ask for treatment preference. If so, they were allocated to the preferred treatment and indifferent patients were randomised.

## 20. * Intervention(s), exposure(s).

Give full and clear descriptions or definitions of the nature of the interventions or the exposures to be reviewed.

The preference cohort.

## 21. * Comparator(s)/control.

Where relevant, give details of the alternatives against which the main subject/topic of the review will be compared (e.g. another intervention or a non-exposed control group). The preferred format includes details of both inclusion and exclusion criteria.

The randomised cohort.

## 22. * Types of study to be included.

Give details of the types of study (study designs) eligible for inclusion in the review. If there are no restrictions on the types of study design eligible for inclusion, or certain study types are excluded, this should be stated. The preferred format includes details of both inclusion and exclusion criteria.

Patient preference trials.

## 23. Context.

Give summary details of the setting and other relevant characteristics which help define the inclusion or exclusion criteria.

We included PPTs describing results of both the randomised and preference cohort, as long as in both cohorts patients met the same in- and exclusion criteria and were treated according to the same treatment protocol. We excluded trials in which allocation was based on doctors' preference, without available separate data for the randomised and preference cohort, with economical primary outcomes, or with nonclinical populations. We did not exclude trials based on quality criteria, as no quality assessment for PPTs has yet been developed and current criteria predominantly relate to concealment of randomisation (consequently quality assessment and variability between trials was not applicable). Furthermore, it was decided not to include older PPTs (before 2005), as it is important to consider the value of this design for current daily practice. A previous systematic review addressing on the value of PPTs was published in 2005, which can be used to interpret results from older studies.

For peer review only

## 24. * Main outcome(s).

Give the pre-specified main (most important) outcomes of the review, including details of how the outcome is defined and measured and when these measurement are made, if these are part of the review inclusion criteria.

The primary outcomes are external and internal validity. Whether patients' preference influences external validity, data will be extracted on participation rates: i) the overall participation rate of eligible patients in the PPT and ii) the proportion of patients accepting randomisation. To assess if a specific patient group accepts randomisation, data will be extracted on baseline characteristics of the randomised and preference cohort of within a PPT separately. These characteristics will be categorised into sociodemographic and clinical factors. Following, these factors will be compared between the randomised and preference cohorts of PPTs. Whether patients' preference influences internal validity, data will be extracted on lost to follow-up, cross-overs, and primary outcomes of the randomised and preference cohort within a PPT separately. Following, these outcomes will be compared between the randomised and preference cohorts of PPTs. The primary outcomes of PPTs will be identified through explicit statements, study hypotheses, reported power analyses, and will be checked="checked" value="1" on similarity with the study protocol. If this is not sufficient, the most likely primary outcome will be chosen by consensus.

### Timing and effect measures

To compare the primary outcomes between the randomised and preference cohorts within PPTs, the treatment effect of the experimental vs. control treatment of the randomised cohort will be compared with the treatment effect of the experimental vs. control treatment of the preference cohort.

## 25. * Additional outcome(s).

List the pre-specified additional outcomes of the review, with a similar level of detail to that required for main outcomes. Where there are no additional outcomes please state 'None' or 'Not applicable' as appropriate to the review

Separate analyses on adjusted and non-adjusted primary outcomes will be performed.

### Timing and effect measures

Not applicable

## 26. * Data extraction (selection and coding).

Give the procedure for selecting studies for the review and extracting data, including the number of researchers involved and how discrepancies will be resolved. List the data to be extracted.

The two first authors will independently screen the citations and abstracts for eligible articles using a pre-piloted standardised data-form (Covidence; Veritas Health Innovation, Melbourne, VIC, Australia). Disagreements will be discussed at steering group meetings. The same two authors will extract data with the use of the same data-form. We will consider multiple publications reporting on the same trial as one single

For peer review only

trial for our analyses.

## 27. * Risk of bias (quality) assessment.

State whether and how risk of bias will be assessed (including the number of researchers involved and how discrepancies will be resolved), how the quality of individual studies will be assessed, and whether and how this will influence the planned synthesis.

We will not exclude trials based on quality criteria, as no quality assessment for PPTs has yet been developed and current criteria predominantly relate to concealment of randomisation (consequently quality assessment and variability between trials do not apply).

## 28. * Strategy for data synthesis.

Give the planned general approach to synthesis, e.g. whether aggregate or individual participant data will be used and whether a quantitative or narrative (descriptive) synthesis is planned. It is acceptable to state that a quantitative synthesis will be used if the included studies are sufficiently homogenous.

The level of sought data are summary estimates (aggregate data). A quantative synthesis is planned. To realize the comparison of the primary outcomes of randomised and preference cohorts, probably a reanalysis needs to be conducted. Because the trials probably involved a range of diseases, outcome measures, and sample sizes, different treatment effects scales it is neccesary to convert these into standardised effect sizes in a reanalysis. Treatment effects are calculated directly for continuous outcome variables as standardised mean differences (difference in means divided by the pooled standard deviation). For binary outcomes log odds ratios are calculated and converted into standardised effect size differences. In case none of the patients in the preference cohort choose the control treatment, the treatment effect of the experimental treatment will be compared with the control treatment of the randomised cohort. Only trials for which a 'net' effect (primary outcome minus baseline value of the primary outcome) can be calculated, will be included in the meta-analyses. In case the 'net' effect is missing, but baseline values and primary outcomes are available, the SD will be estimated. A final meta-regression will be performed using a wald test to compare the standardised treatment effects. A P 0.05 is considered a significant difference. R's programming environment will be used (version 3.5.1 , R Foundation for Statistical Computing, Vienna, Austria). Five researches are involved. Disagreements are discussed at steering group meetings.

## 29. * Analysis of subgroups or subsets.

Give details of any plans for the separate presentation, exploration or analysis of different types of participants (e.g. by age, disease status, ethnicity, socioeconomic status, presence or absence or co-morbidities); different types of intervention (e.g. drug dose, presence or absence of particular components of intervention); different settings (e.g. country, acute or primary care sector, professional or family care); or different types of study (e.g. randomised or non-randomised).

Adjusted and non-adjusted primary outcomes.

## 30. * Type and method of review.

Select the type of review and the review method from the lists below. Select the health area(s) of interest for your review.

For peer review only - http://bmjopen.bmj.com/site/about/guidelines.xhtml

## Type of review

Cost effectiveness
No

Diagnostic
No

Epidemiologic
Yes

Individual patient data (IPD) meta-analysis
No

Intervention
No

Meta-analysis
No

Methodology
No

Narrative synthesis
No

Network meta-analysis
No

Pre-clinical
No

Prevention
No

Prognostic
No

Prospective meta-analysis (PMA)
No

Review of reviews
No

Service delivery
No

Synthesis of qualitative studies
No

Systematic review
Yes

Other
No

## Health area of the review

Alcohol/substance misuse/abuse
No

Blood and immune system
No

Cancer
No

Cardiovascular
No

Care of the elderly
No

Child health
No

For peer review only

PROSPERO
International prospective register of systematic reviews

Complementary therapies
No
Crime and justice
No
Dental
No
Digestive system
No
Ear, nose and throat
No
Education
No
Endocrine and metabolic disorders
No
Eye disorders
No
General interest
Yes
Genetics
No
Health inequalities/health equity
No
Infections and infestations
No
International development
No
Mental health and behavioural conditions
No
Musculoskeletal
No
Neurological
No
Nursing
No
Obstetrics and gynaecology
No
Oral health
No
Palliative care
No
Perioperative care
No
Physiotherapy
No
Pregnancy and childbirth
No
Public health (including social determinants of health)
No
Rehabilitation
No
Respiratory disorders
No
Service delivery
No
Skin disorders
No
Social care
No

For peer review only

Surgery
No
Tropical Medicine
No
Urological
No
Wounds, injuries and accidents
No
Violence and abuse
No

## 31. Language.

Select each language individually to add it to the list below, use the bin icon to remove any added in error.

There is an English language summary.

## 32. Country.

Select the country in which the review is being carried out from the drop down list. For multi-national collaborations select all the countries involved.
Netherlands

## 33. Other registration details.

Give the name of any organisation where the systematic review title or protocol is registered (such as with The Campbell Collaboration, or The Joanna Briggs Institute) together with any unique identification number assigned. (N.B. Registration details for Cochrane protocols will be automatically entered). If extracted data will be stored and made available through a repository such as the Systematic Review Data Repository (SRDR), details and a link should be included here. If none, leave blank.

## 34. Reference and/or URL for published protocol.

Give the citation and link for the published protocol, if there is one

Give the link to the published protocol.

Alternatively, upload your published protocol to CRD in pdf format. Please note that by doing so you are consenting to the file being made publicly accessible.

No I do not make this file publicly available until the review is complete

Please note that the information required in the PROSPERO registration form must be completed in full even if access to a protocol is given.

## 35. Dissemination plans.

Give brief details of plans for communicating essential messages from the review to the appropriate audiences.

## Do you intend to publish the review on completion?

Yes

## 36. Keywords.

Give words or phrases that best describe the review. Separate keywords with a semicolon or new line. Keywords will help users find the review in the Register (the words do not appear in the public record but are included in searches). Be as specific and precise as possible. Avoid acronyms and abbreviations unless these are in wide use.

Comprehensive cohort design, patietns preference trial, pateitns'prference, randomised control trials.

For peer review only

**PROSPERO**
**International prospective register of systematic reviews**

National Institute for Health Research

## 37. Details of any existing review of the same topic by the same authors.

Give details of earlier versions of the systematic review if an update of an existing review is being registered, including full bibliographic reference if possible.

## 38. * Current review status.

Review status should be updated when the review is completed and when it is published. For newregistrations the review must be Ongoing.
Please provide anticipated publication date

Review_Ongoing

## 39. Any additional information.

Provide any other information the review team feel is relevant to the registration of the review.

I'm very sorry that I wrote the fields #24-#29 in past time during my revisions, I have corrected this. Currently the data extraction is almost done. Since some deley has occured, we think we will finish the data extraction and analyses in March 2019 instead of past November (I've amended this part). We think prospero is a very usefull and valuable registration, therefore we hope you will register the study.

## 40. Details of final report/publication(s).

This field should be left empty until details of the completed review are available.

Give the link to the published review.

For peer review only

# PRISMA 2009 Checklist

| Section/topic | # | Checklist item | Reported on page # |
|---|---|---|---|
| **TITLE** | | | |
| Title | 1 | Identify the report as a systematic review, meta-analysis, or both. | 1 |
| **ABSTRACT** | | | |
| Structured summary | 2 | Provide a structured summary including, as applicable: background; objectives; data sources; study eligibility criteria, participants, and interventions; study appraisal and synthesis methods; results; limitations; conclusions and implications of key findings; systematic review registration number. | 4,5 |
| **INTRODUCTION** | | | |
| Rationale | 3 | Describe the rationale for the review in the context of what is already known. | 6 |
| Objectives | 4 | Provide an explicit statement of questions being addressed with reference to participants, interventions, comparisons, outcomes, and study design (PICOS). | 6 |
| **METHODS** | | | |
| Protocol and registration | 5 | Indicate if a review protocol exists, if and where it can be accessed (e.g., Web address), and, if available, provide registration information including registration number. | 7 |
| Eligibility criteria | 6 | Specify study characteristics (e.g., PICOS, length of follow-up) and report characteristics (e.g., years considered, language, publication status) used as criteria for eligibility, giving rationale. | 7 |
| Information sources | 7 | Describe all information sources (e.g., databases with dates of coverage, contact with study authors to identify additional studies) in the search and date last searched. | 7 |
| Search | 8 | Present full electronic search strategy for at least one database, including any limits used, such that it could be repeated. | 7 |
| Study selection | 9 | State the process for selecting studies (i.e., screening, eligibility, included in systematic review, and, if applicable, included in the meta-analysis). | 7,8 |
| Data collection process | 10 | Describe method of data extraction from reports (e.g., piloted forms, independently, in duplicate) and any processes for obtaining and confirming data from investigators. | 7 |
| Data items | 11 | List and define all variables for which data were sought (e.g., PICOS, funding sources) and any assumptions and simplifications made. | 7,8 |
| Risk of bias in individual studies | 12 | Describe methods used for assessing risk of bias of individual studies (including specification of whether this was done at the study or outcome level), and how this information is to be used in any data synthesis. | 7,8 |
| Summary measures | 13 | State the principal summary measures (e.g., risk ratio, difference in means). | 7,8 |
| Synthesis of results | 14 | Describe the methods of handling data and combining results of studies, if done, including measures of consistency (e.g., $I^2$) for each meta-analysis. | 9,10 |

For peer review only - http://bmjopen.bmj.com/site/about/guidelines.xhtml

# PRISMA 2009 Checklist

| Section/topic | # | Checklist item | Reported on page # |
|---|---|---|---|
| Risk of bias across studies | 15 | Specify any assessment of risk of bias that may affect the cumulative evidence (e.g., publication bias, selective reporting within studies). | 7-10 |
| Additional analyses | 16 | Describe methods of additional analyses (e.g., sensitivity or subgroup analyses, meta-regression), if done, indicating which were pre-specified. | 9,10 |
| **RESULTS** | | | |
| Study selection | 17 | Give numbers of studies screened, assessed for eligibility, and included in the review, with reasons for exclusions at each stage, ideally with a flow diagram. | 10 |
| Study characteristics | 18 | For each study, present characteristics for which data were extracted (e.g., study size, PICOS, follow-up period) and provide the citations. | 10 |
| Risk of bias within studies | 19 | Present data on risk of bias of each study and, if available, any outcome level assessment (see item 12). | n/a (see page 7-10) |
| Results of individual studies | 20 | For all outcomes considered (benefits or harms), present, for each study: (a) simple summary data for each intervention group (b) effect estimates and confidence intervals, ideally with a forest plot. | 10 |
| Synthesis of results | 21 | Present results of each meta-analysis done, including confidence intervals and measures of consistency. | 10-13 (figure 3-5) |
| Risk of bias across studies | 22 | Present results of any assessment of risk of bias across studies (see Item 15). | n/a (see page 7-10) |
| Additional analysis | 23 | Give results of additional analyses, if done (e.g., sensitivity or subgroup analyses, meta-regression [see Item 16]). | 10-13 |
| **DISCUSSION** | | | |
| Summary of evidence | 24 | Summarize the main findings including the strength of evidence for each main outcome; consider their relevance to key groups (e.g., healthcare providers, users, and policy makers). | 14 |
| Limitations | 25 | Discuss limitations at study and outcome level (e.g., risk of bias), and at review-level (e.g., incomplete retrieval of identified research, reporting bias). | 15 |
| Conclusions | 26 | Provide a general interpretation of the results in the context of other evidence, and implications for future research. | 14-16 |
| **FUNDING** | | | |
| Funding | 27 | Describe sources of funding for the systematic review and other support (e.g., supply of data); role of funders for the systematic review. | 5 |

For peer review only

# PRISMA 2009 Checklist

*From:* Moher D, Liberati A, Tetzlaff J, Altman DG, The PRISMA Group (2009). Preferred Reporting Items for Systematic Reviews and Meta-Analyses: The PRISMA Statement. PLoS Med 6(6): e1000097. doi:10.1371/journal.pmed1000097

For more information, visit: **www.prisma-statement.org**.



For peer review only

For peer review only - http://bmjopen.bmj.com/site/about/guidelines.xhtml

BMJ Open

# Partially randomised patient preference trials as an alternative design to randomised controlled trials: systematic review and meta-analyses



| | |
|---|---|
| Journal: | *BMJ Open* |
| Manuscript ID | bmjopen-2019-031151.R1 |
| Article Type: | Original research |
| Date Submitted by the Author: | 12-Jul-2019 |




| | |
|---|---|
| Complete List of Authors: | Wasmann, Karin; Amsterdam UMC - Locatie AMC, ;<br>Wijsman, Pieta; Spaarne Gasthuis<br>van Dieren, Susan; Amsterdam UMC - Locatie AMC<br>Bemelman, Willem; Amsterdam UMC - Locatie AMC<br>Buskens, Christianne; Amsterdam UMC - Locatie AMC |




| | |
|---|---|
| <b>Primary Subject Heading</b>: | Epidemiology |
| Secondary Subject Heading: | Epidemiology, Health policy, Health services research, Research methods |
| Keywords: | Randomised controlled trial, comprehensive cohort design, internal validity, external validity, patients’ preference, randomised pateint preference trial |



SCHOLARONE™
Manuscripts

# Partially randomised patient preference trials as an alternative design to randomised controlled trials: systematic review and meta-analyses

Karin A. Wasmann, MD[1], Pieta C. Wijsman[2], MD, Susan van Dieren[3], PhD, Willem A. Bemelman[1*], PhD and Christianne J. Buskens[1], PhD

1. Department of surgery, Amsterdam UMC, University of Amsterdam, Amsterdam, the Netherlands, k.a.wasmann@amsterdamumc.nl, w.a.bemelman@amc.nl, c.j.buskens@amc.nl

2. Department of internal medicine, Spaarne Gasthuis, Hoofddorp, the Netherlands, pwijsman@spaarnegasthuis.nl

3. Department of Epidemiology, University of Amsterdam, Amsterdam, The Netherlands, s.vandieren@amc.nl

*This author is a full professor

**Corresponding author during review process**

Drs. Karin A T G M Wasmann

Department of Surgery, Amsterdam UMC, University of Amsterdam

Meibergdreef 9, 1105 AZ Amsterdam, the Netherlands

k.a.wasmann@amsterdamumc.nl

**Corresponding author**

Dr. Christianne J Buskens

Department of Surgery, Amsterdam UMC, University of Amsterdam

Meibergdreef 9, 1105 AZ Amsterdam, the Netherlands

c.j.buskens@amc.nl

**Word count:** 3751

For peer review only

**Abstract**

**Objective:** Randomised controlled trials (RCT) are the gold standard to provide unbiased data. However, when patients have a treatment preference, randomisation may influence participation and outcomes (e.g. external and internal validity). The aim of this study was to assess the influence of patients' preference in RCTs by analysing partially randomised patient preference trials (RPPT); a RCT and preference cohort combined.

**Design:** Systematic review and meta-analyses.

**Data Sources:** MEDLINE, Embase, PsychINFO, and the Cochrane library.

**Eligibility Criteria for selecting studies:** RPPTs published between Jan, 2005 and Oct, 2018, reporting on allocation of patients to random- and preference cohorts were included.

**Data extraction and synthesis:** Two independent reviewers extracted data. The main outcomes were the difference in external validity (participation and baseline characteristics) and internal validity (lost to follow-up, cross-over and the primary outcome) between the random- versus the preference cohort within each RPPT, compared in a meta-regression using a Wald test. Risk of bias was not assessed, as no quality assessment for RPPTs has yet been developed.

**Results:** In total 117 of 3734 identified articles met screening criteria and 44 were eligible (24873 patients). The participation rate in RPPTs was >95% in 14 trials(range:48-100%) and the randomisation refusal rate was >50% in 26 trials(range:19-99%). Higher education, female, older age, race, and prior experience with one treatment-arm were characteristics of patients declining randomisation. The lost to follow-up and cross-over rate were significantly higher in the randomised cohort compared to the preference cohort. Following the meta-analysis, the reported primary outcomes were comparable between both cohorts of the RPPTs, mean difference 0.093(95%CI:-0.178;0.364, *P*=0.502).

For peer review only

**Conclusions:** Patients' preference led to a substantial proportion of a specific patient group refusing randomisation, while it did not influence the primary outcome within a RPPT. Therefore, RPPTs could increase external validity without compromising the internal validity compared with RCTs.

**Trial registration:** PROSPERO, #CRD42019094438.

**Key words:** Randomised controlled trials, comprehensive cohort design, internal validity, external validity, patients' preference, randomised patient preference trials

For peer review only

## Article Summary

### Strengths and limitations of this study

- This systematic review and meta-analyses of partially randomised patient preference trials (RPPTs) provide unique data on external and internal validity between randomised and patients' preference cohorts.
- It provides a valid alternative study design to an RCT, especially when patient preferences can be expected.
- It was not possible to objectively establish the quality of included trials, as there is currently no valid critical appraisal tool to apply for a RPPT.
- Uniform counselling is of crucial importance in RPPTs, which has not been standardly reported in the included studies.

For peer review only

**Introduction:**

Randomised controlled trials (RCTs) are suggested to provide the most reliable evidence for treatment efficacy.[1] However, participants are no passive recipients of interventions. Patients with a treatment preference may decline enrolment to avoid being randomised to their non-preferred treatment. Consequently, treatment preferences can decrease the generalizability of RCT results to the clinical population (i.e. reduce external validity). Additionally, trials comparing experimental *vs* standard treatment, are likely to include patients preferring experimental treatment, as trial participation is not needed for patients preferring standard treatment, further reducing external validity. Internal validity may be reduced, as randomisation to the (non-) preferred strategy could influence adherence to treatment protocol and study outcomes. Subjective study outcomes can directly be affected by treatment preference, whereas objective outcomes are most likely affected indirectly via adherence (so called reluctant acquiescence phenomenon). Especially for an unblinded trial comparing treatments of significant different nature (e.g. medical *vs* surgical) the RCT could be an inappropriate design. Throughout the years, several approaches, using various names, have been proposed as alternative designs to diminish the influence of patients' preference on validity: a partially randomised patient preference trial (RPPT), a comprehensive cohort trial, a patient preference trial, and more.[2] In general the aim of these designs is to treat patients with a preference for a treatment strategies accordingly, whereas only those patients without a distinct preference will be randomised in the usual way.[3] In the era of patients becoming more active participants in research, the use of RPPTs increases. The two previous systematic reviews addressing influence of preference on validity, concluded that this influence was limited.[4,5] However, one review only included studies addressing psychotherapy, and the other dates from 2005. So far, the value of the RPPT remains unclear, nor has it been addressed in the Oxford Levels of Evidence (CEBM).[6]

The aim of the study was to assess the influence of patients' preference following randomisation in current daily clinical practice, by comparing randomised cohorts with preference cohorts within all RPPTs published since 2005. Two hypotheses were tested: 1) Patients' preference will negatively influence participation in RCTs, decreasing external validity. Therefore, the external validity of a RPPT will be higher. 2) Patients' preferences will influence adherence and outcomes in RCTs, decreasing

For peer review only

internal validity. However, as only the remaining indifferent patients will be included in the RCT cohort of a RPPT, this RCT cohort can be considered as the true gold standard for internal validity.

For peer review only

**METHODS:**

**Design**

A systematic review and meta-analyses of RPPTs was conducted. This study is reported in accordance with the Cochrane Handbook for Systematic Reviews of Interventions[7] and the Preferred Reportion Items for Systematic Reviews and Meta-Analyses statement (supplement 1).[8] The study protocol is available online (supplement 2). The protocol is registered at PROSPERO (#CRD42019094438).

**Data sources and searches**

A search in PubMed, Embase, Psycinfo, and the Cochrane Library for RPPTs published between Jan 1, 2005 and Oct 5, 2018 was executed without language restriction with the assistance of a librarian. The subject in the search strategy was RPPT and possible aliases of RPPT (see Pubmed Search Strategy). Database searches were supplemented by hand searching reference lists of relevant articles. Additionally, authors were contacted to seek for data from unpublished studies identified. Non-English-language articles were translated for possible inclusion.

**Study selection**

RPPTs describing results of both the randomised- and preference cohort, as long as in both cohorts patients met the same in- and exclusion criteria and were treated according to the same treatment protocol were included. Trials in which a two-stage randomised design was conduction, allocation was based on doctors' preference, without available separate data for the randomised and preference cohort, with economical primary outcomes, or with nonclinical populations were excluded. Furthermore, it was decided not to include older RPPTs (before 2005), as it is important to consider the value of this design for current daily practice. A previous systematic review addressing on the value of RPPTs was published in 2005, which can be used to interpret results from older studies.[4]

**Data extraction**

The two first authors independently screened the citations and abstracts for eligible articles using a pre-piloted standardised data-form (Covidence; Veritas Health Innovation, Melbourne, VIC, Australia). Disagreements were discussed at steering group meetings.

For peer review only

The same two authors extracted data with the use of the same data-form. Multiple publications reporting on the same trial were considered as one single trial for these analyses.

The level of sought data were summary estimates. Authors were contacted for further information when necessary. In case they were not forthcoming, the study was included in the review, but excluded from our reanalysis and or meta-analyses.

**Risk of bias assessment**

Quality assessment of the trials was not performed, as no quality assessment for RPPTs has yet been developed and current criteria predominantly relate to concealment of randomisation (e.g. ROBINS-I and Cochrane risk of bias) consequently quality assessment and variability between trials was not applicable.[9,10] Since the outcomes of each trial greatly differed, also the risk of bias assessment for systematic reviews (e.g. GRADE) was not applicable.[11]

**Outcomes**

The primary outcomes were external and internal validity between randomised and preference cohorts within RPPTs. To analyse whether patients' preference influenced external validity, data were extracted on participation rates in the randomised and preference cohort. To assess if a specific patient group accepted randomisation, data were extracted on baseline characteristics of the randomised and preference cohort of a RPPT separately. These characteristics were categorised into sociodemographic and clinical factors. Following, these factors were compared between the randomised and preference cohorts of RPPTs.

To analyse whether patients' preference influenced internal validity, data were extracted on lost to follow-up, cross-overs, and primary outcomes of the randomised and preference cohort of a RPPT separately. Following, these outcomes were compared between the randomised and preference cohorts within RPPTs. The primary outcomes of RPPTs were identified through explicit statements, study hypotheses, reported power analyses, and were checked on similarity with the study protocol. If this was not sufficient, the most likely primary outcome was chosen by consensus (KW and SvD), or the

For peer review only

study was excluded. To compare the primary outcomes between the randomised and preference cohorts within RPPTs, the outcome effects were compared between the randomised cohort and the preference cohort. It is emphasized that comparisons of outcome between randomised and preference cohorts are subject to bias, and if not done by the study itself, it was not possible to adjust for confounding factors. If in studies the adjusted and non-adjusted primary outcomes were available, the adjusted outcomes were used. Following, separate analyses on adjusted and non-adjusted primary outcomes were performed.

For peer review only

**Statistical analysis**

The randomisation rate, participation rate, and difference in baseline characteristics between the randomised and preference cohorts were explored and described, but not compared using statistics. To assess differences in baseline characteristics, mean and SDs were compared. If median IQRs were reported, it was converted to mean and SDs.[12] When baseline characteristics were presented per experimental and control group, the sum of mean and SDs of these two groups were calculated for the randomised and preference cohorts using a weighted t-test. The lost to follow-up and cross-over rates were compared using a random effect model meta-analysis for proportions.

To realise the comparison of the primary outcomes of randomised and preference cohorts, a reanalysis was conducted. Because the trials involved a range of diseases, outcome measures, and sample sizes, different treatment effects scales were converted into standardised effect sizes in the reanalysis. Treatment effects were calculated directly for continuous outcome variables as standardised mean differences (difference in means divided by the pooled standard deviation). For binary outcomes log odds ratios were calculated and converted into standardised effect size differences.[13] In case none of the patients in the preference cohort choose the control treatment, the treatment effect of the experimental treatment was compared with the control treatment of the randomised cohort. Only trials for which a 'net' effect (primary outcome minus baseline value of the primary outcome) could be calculated, were included in the meta-analyses. In case the 'net' effect was missing, but baseline values and primary outcomes were available, the SD was estimated.[14] Heterogeneity was not assessed as trials outcomes were different for each study included. Meta-analysis of randomised versus preference cohort was performed using a random effect model with an inverse variance weighting. A final meta-regression was performed using a Wald test to compare the standardised treatment effects.

A $P < 0.05$ was considered a significant difference. R's programming environment was used (version 3.5.1 , R Foundation for Statistical Computing, Vienna, Austria).

**Patient and Public Involvement**

There was no direct involvement of patients or the public in the development of the research question, selection of the outcomes measures, design and implementation of the study, or interpretation of the results.

For peer review only

**RESULTS**

In total 117, out of 3734 records identified, were full-text screened. Fifty-eight partially randomised patient preference trials from 2005 onwards were found, of which 44 (including 24 873 patients) were eligible for at least basic data extraction (Table 1), and 20 could be included in the meta-analyses (Prisma flowchart Figure 1).[15-72] Exclusion reasons for the meta-analyses were, no availability of both treatment outcomes in the randomised and preference cohort separately in 14 trials[15,16,18,19,23,24, 27,30,31,34,39,41,42,63], no availability of standard deviations, which could also not be converted from other available data in five trials[21,29,49,52,62], and the number of events or the power of one or both cohort(s) was too low to perform separate randomised and preference analyses in five trials.[25,28,40,55,72] The trials covered a wide range of clinical areas and interventions. The main areas were Gynaecology (n= 11), Orthopaedics (n= 10), and Psychiatry (n= 5). Of the 44 included trials, 32 trials compared an intervention versus conservative treatment, including 16 surgical interventions (Table 1). In all trials but one, if patients refused randomisation they received their preference treatment (Figure 2). In the other study a Zelen Randomisation was performed, randomising all eligible patients and afterwards asking for their consent to participate in the randomised arm or if they preferred the other intervention.[34] Parental preference was relevant in five trials involving children, as permission of parents was required and the preference between patients and parents could not be distinguished.[24,29,42,56,63]

*External validity*

Following results concern the influence of patients' preference on external validity. Information on the number of eligible patients who agreed to participate (in either the randomised or preference cohort), was available in 39 out the 44 RPPTs. The participation rate of eligible patients in the RPPTs ranged from 48% to 100%. In which 16 RPPTs reported a participation rate higher than 80%, and 14 RPPTs a participation rate higher than 95%. Of these included participants in the 44 RPPTs, 18% to 99% declined randomisation (hence these patients were included in the preference cohort). The randomisation refusal rate was more than 50% in 26 RPPTs.

To assess if a specific patient group accepted randomisation, 35 of the 44 RPPTs reported at least one comparison between randomised and preference cohorts on baseline sociodemographic factors. At

For peer review only

least one significant difference between randomised and preference cohorts was found in 20 of the 35 trials. Overall, 38 significant differences were found in 161 sociodemographic comparisons (24%). The proportion of significant findings was not dependent on sample size (smaller trials n< 300; 19/85, 22% and larger trials n≥ 300; 19/76, 25%). Patients with a preference compared with those accepting randomisation were more likely to be older, female, higher educated, employed, Caucasian, not obese, non-smokers, unmarried, and experienced with one treatment arm (Supplement 3, Table).

Thirty-four of the 44 RPPTs reported at least one comparison between randomised and preference cohorts on clinical baseline characteristics. At least one significant difference was found in 20 of the 34 trials. Overall, 36 significant differences were found in 220 clinical comparisons (16%). The proportion of significant findings was not dependent on sample size (smaller trials n< 300; 12/78, 15% and larger trials n≥ 300; 24/142, 17%). Patients with a preference had more severe clinical problems in seven trials and less severe clinical problems in ten trials, while in the remaining three trials no consistent pattern could be found (Supplement 3, Table).

*Internal validity*

Following results concern the influence of patients' preference on internal validity. Information on lost to follow-up in both the randomised and preference cohorts was available in 33 of the 44 RPPTs. For the randomised cohorts, the proportion of individuals lost to follow-up was < 10% in 14 trials, 10% to < 20% in 9 trials, and ≥ 20% in 10 trials. For the preference cohorts the corresponding numbers of trials were 17, 9, and 7. The mean percentage of participants lost to follow-up was significantly higher in the randomised cohorts (16·1% (SD 16·8%)) compared with the preference cohorts (13.3% (SD 14.7%)), RR 1.3, (CI95% 1.0 – 1.6), $P = 0.03$).

Information on cross-overs in both the randomised and preference cohorts was available in 20 of 44 RPPTs. For the randomised cohorts, the proportion of individuals that crossed-over to the other study treatment was < 10% in 11 trials, 10% to < 20% in 5 trials, and ≥ 20% in 4 trials. For the preference cohorts the corresponding numbers of trials were 14, 5, and 1. The mean percentage of cross-overs was significantly higher in the randomised cohorts (14.5% (SD 16.9%)) compared with the preference cohorts (6.3**%** (SD 11.5%)), RR 2.6 (CI95% 1.7-3.9), $P < 0.001$).

For peer review only

To assess the influence of patients' preference on primary outcomes, for 20 of the 44 RPPTs it was possible to perform reanalyses using standardised effect sizes (Figure 1).

Figure 3 shows the magnitude of the experimental treatment effect over the control treatment effect of the randomised and preference cohort separately using standardised effect sizes. The trial are listed by sample size. A positive experimental treatment effect was seen in 13 trials. The influence of patients' preference on primary outcomes according to different standardised treatment effects between randomised and preference cohorts was small, in 13 of the 20 trials (65%) this was 0.2 or less (scale -2 to 2), in 5 trials (25%) between 0.21 and 0.5, and in 2 trials (10%) higher than 0.5. Of the 20 RPPTs, the overall mean difference in primary outcome between randomised and preference cohorts was not significantly different, 0.093 (95%CI -0.178 to 0.364) $P$ = 0.502 (Figure 2). Only two trials showed a significant different treatment effect between the randomised and preference cohort.[68,69] In both trials the experimental treatment effect was favourable over the control treatment effect in both in the randomised and preference cohort, but the favourable effect of the experimental treatment was significantly greater in the preference cohort. Both RPPTs compared acupuncture versus conservative treatment. In one trial the improvement of the osteoarthritis index in patients with osteoarthritis of the knee or hip was assessed, the other trial assessed the functional ability score in patients with chronic low back pain.

In seven of these 20 trials, an adjusted primary outcome for baseline confounders was available[22,32,35,37,60,64,65] In these trials, the mean difference in primary outcome between randomised and preference cohorts was even smaller -0.026 (95%CI -0.263 to 0.211) $P$ = 0.832. In 18 trials (also) a non-adjusted primary outcome was available. Using these outcomes, the mean difference in primary outcomes was 0.228 (95%CI -0.117 to 0.572) $P$ = 0.196 (Figure 4 and 5).

For peer review only

**DISCUSSION:**

These study results challenge the current consensus about the hierarchy of study designs. Our results indicate that patients' preference led to a substantial proportion of patient refusing randomisation (refusal of randomisation was more than 50% in 26 trials), while it did not affect the primary outcome of a RPPT.

Regarding our first hypothesis, it can be conclude that patients' preference does negatively influence participation to RCTs as demonstrated by the low participation to the randomised cohort in RPPTs. The participation in the RPPTs was remarkably high (ranging from 48% - 100%), improving external validity when compared with the classic RCT (ranging from <0.001 - 40%).[73] Cautiously, it could be argued that a typical patient group characterised by e.g. higher education, Caucasian race, and non-obese individuals are more likely to refuse randomisation. In contrast, differences in clinical characteristics showed no consistent pattern in the randomised or preference cohorts. Therefore, not including a patients' preference cohort in a trial could result in a potential loss of inclusions of a specific patient group, further decreasing external validity.

Regarding our second hypothesis, it can be conclude that patients' preference does not significantly affect the primary outcome of a RPPT, as the primary outcomes of patients in the randomised and preference cohorts were similar. Since the aim of a RPPT is to treat patients according to their preference, it can be assumed that the randomised cohort of a RPPT includes patients indifferent to the type of treatment. Following, it is unlikely that outcomes of randomised patients will be biased by treatment preference. Hence, they could be seen as the gold standard. Lost to follow-up and cross-overs were significantly higher in the randomised cohort compared with the preference cohort. As a result, the data of the preference cohort could be interpreted more easily than the randomised data. Perhaps, consciously choosing a treatment ensures a certain dedication and tolerance for the treatment.

Our results are strengthened by the previous systematic review of King et al, including RPPTs from 1966 to 2004. Based on their results, they also postulated that treatment preference influences the willingness to accept randomisation, and that the evidence of its significant affect on internal validity is low.[4] A possible limitation of their study is that they did not measure patients' preference as specifically as in our analyses, since they also included a minority of two-stage randomised trials, as physician preference.

For peer review only - http://bmjopen.bmj.com/site/about/guidelines.xhtml

An RCT is once designed to reliably compare medication to placebo.[74] In the hierarchy of research designs, the results of RCTs are considered to be evidence of the highest grade. Lessons learned from the history of RCT, early studies from 1970 and 1980s suggested that observational studies suffer too much from confounders and frequently result in overestimation of treatment effects compared with RCTs.[75,76] Consequently, many experts advocated that results of observational studies should not be used for defining evidence-based medical care: *''If the study wasn't randomized, we suggest that you stop reading it and go on to the next article''.*[77] However, two updates of this work including studies between 1985 and 1995 found little evidence that estimates of treatment effects in observational studies are consistently larger from those obtained in RCTs.[78,79] It is suggested that observational studies have methodologically improved over time with the use of a control group, carefully defining in- and exclusion criteria, and by better understanding confounders. The fundamental criticism of the RPPT could be that within the preference cohort the unrecognized confounding factors may distort the results. Yet, our results showed that preference cohorts provide valid information comparable with the randomised results.

Today, the classic levels of evidence are subject of debate, as the disadvantages of RCTs have become more insightful in modern practice. In general, patients participating in RCTs are highly selected. Less than 10% of patients participate in trials, partly due to exclusion of patients with a specific treatment preference.[80] This limits the extrapolation of RCT results to patients seen in routine practice. Another consequence is that the majority of trials takes several years to be completed. This not only causes a burden on health research costs, but also results in a questionable ethical dilemma**.** Developments are fast and the relevance of trials may therefore change over time. Consequently, if an RCT is optimally designed but takes too long, the results will be outdated.

This especially applies, when designing a trial in which it can be foreseen that patients' preference will be a prominent factor. For example in trials comparing treatments of significant different nature (medical versus surgical). Anticipation on the expected patients' preference by eliminating this factor is at the expense of the validity of a lot of RCTs. Especially when patient-centred outcomes are used, one should consider whether the most important patients group has been excluded. Trials must be internally valid, but lack of consideration of external validity causes the widespread underuse of

For peer review only

treatments -that showed superiority in RCTs- in routine practice. Moreover, in these situations a RPPT could be the superior design over an RCT.

RPPTs provide unique data on external and internal validity as the patients in the preference cohort are followed according the same conditions as the patients in the randomised cohorts. A limitation of our review is that interventions and settings between RPPTs were very diverse. On the other hand, because of this diversity, it could also be stated that randomised data and preference data often produce similar results; in all kind of settings. Concerning the assessment of external validity, it should be noted that in only a minority of trials the differences in sociodemographic and clinical parameters between the cohorts of a RPPT were evident. Furthermore, in some cases none of the patients in the preference cohort choose the control treatment. In these cases, the treatment effect of the experimental treatment was compared with the control treatment of the randomised cohort. These are not optimal comparisons, but considered to be more appropriate then excluding these data. Moreover, as the idea of RPPTs is a relatively new concept, various terms were used in the inclusion period of this systematic review. In the publication of Walter et al in 2017 different concepts were compared and they clearly defined the terms fully randomised patient preference trial and partially randomised patient preference trial. To achieve a 'fully randomised patient preference trial' preference of all participants should be identified. Therefore, uniform counselling is of crucial importance in RPPTs. The majority of included studies claim to be randomised patient preference trials. However, in most of currently included studies, the details of how patients were counselled has not been addressed. As we can't guarantee that a study identified the preference of all eligible patients, we decided to use the term partially randomised patient preference trials. Another result of the novelty of such a design is that it was not possible to objectively establish the quality of included trials, as there is currently no valid critical appraisal tool to apply for a RPPT. Consequently, our results may have been influenced by the inclusions of flawed trials.

In conclusion, RPPTs seem to be a reliable alternative for RCTs, especially in trials comparing treatments of vastly difference nature (e.g. medical vs surgical) or using patient-centred outcomes. In case patients' preference can be assumed, RPPT enables faster inclusion of a more representative population improving external validity without compromising internal validity.

For peer review only

**Author Contributors:** KW and CB design the study, KW and PW performed the search, KW and SvD did the statistical analyses, KW wrote the first draft with input of CB and WB.

**No competing interests:** All authors have completed the ICMJE uniform disclosure form at www.icmje.org/coi_disclosure.pdf and declare: no support from any organisation for the submitted work; no financial relationships with any organisations that might have an interest in the submitted work in the previous three years; no other relationships or activities that could appear to have influenced the submitted work.

**Acknowledgments:** None.

**Ethics approval**: Not applicable.

**Funding:** This research received no specific grant from any funding agency in the public, commercial or not-for-profit sectors.

**Data sharing:** Anonymised patient level data can be made available on reasonable request after approval from the trial management committee and after signing a data access agreement. Proposals should be directed to the corresponding author. Consent was not obtained for data sharing, but the presented data are anonymised and the risk of identification is low.

**Transparency:** The lead author (CB) affirms that the manuscript is an honest, accurate, and transparent account of the study being reported; that no important aspects of the study have been omitted; and that any discrepancies from the study as planned have been explained.

For peer review only

**Pubmed search strategy:**

5-10-2018

(patient preference design*[tiab] OR patient preference model*[tiab] OR patient preference trial*[tiab] OR patient preference method*[tiab] OR comprehensive cohort stud*[tiab] OR comprehensive cohort design*[tiab] OR patient preference group[tiab] OR patient preference allocation arms[tiab] OR preference allocation[tiab] OR randomized preference trial*[tiab] OR randomised preference trial*[tiab] OR preference arms[tiab] OR preferences[ti] OR treatment preference basis[tiab] OR (patient preference*[tiab] AND random*[ti]) OR (prefer*[ti] AND random*[ti]) OR (registry patient*[tiab] AND randomized[tiab])) AND ("Clinical Trial"[pt] OR trial[ti] OR preference trial[tiab]) AND ("2004/09"[Date - Publication] : "3000"[Date - Publication])

And

((patient preferences[ti] AND clinical trials[ti]) OR nonrandomized[ti] OR (patient preference[ti] AND randomization[ti]) OR (random[ti] AND nonrandom assignment[ti]) OR (randomized[ti] AND non-randomized[ti]) OR (nonrandom assignment[ti]) OR (randomized[ti] AND nonrandomized[ti]) OR (randomi*[tiab] AND preference arm) OR (partially randomized study[tiab] AND "Randomized Controlled Trial"[pt]) OR (unwilling to be randomized[tiab] AND "Randomized Controlled Trial"[pt]) OR (choice[tiab] AND randomisation[tiab] AND "Randomized Controlled Trial"[pt])) AND (random*[tiab]) AND ("Clinical Trial"[pt] OR trial[ti] OR clinical trials[ti]) AND ("2004/09"[Date - Publication] : "3000"[Date - Publication])

"comprehensive cohort*"[tiab] AND ("2004/09"[Date - Publication] : "3000"[Date - Publication])

For peer review only

**Figure and Tables**

**Figure legends:**

**Figure 1.** Study selection according to PRISMA

**Figure 2.** A randomised patient preference trial

**Figure 3.** Forest plot of the preference effect on the primary outcome between the randomised and preference cohort, by comparing the overall treatment effect (standardized effect size) within the randomised cohorts versus the overall treatment effect within the preference cohorts.

**Figure 4.** Forest plot of the preference effect on the primary outcome between the randomised and preference cohort of trials in which the primary outcome is adjusted for confounders. The overall treatment effect (standardized effect size) within the randomised cohorts was compared to the overall treatment effect within the preference cohorts.

**Figure 5**. Forest plot of the preference effect on the primary outcome between the randomised and preference cohort of trials in which the primary outcome is not adjusted for confounders. The overall treatment effect (standardized effect size) within the randomised cohorts was compared to the overall treatment effect within the preference cohorts.

**Supplementary material**

**Supplement 1.** PRISMA checklist

**Supplement 2.** Study protocol

**Supplement 3, table**. Significant sociodemographic findings preference vs randomised cohorts

For peer review only

**Table 1.** Partially randomised patient preference trials included in the review

| Source | Population | No. R. | No. P. | Field | Intervention and comparison groups | Prim. Outcome(s) |
|---|---|---|---|---|---|---|
| Ashok et al,[15] 2005 | Woman presenting for termination of pregnancy | 400 | 86 | Gynaecology | Medical vs surgical termination^+ | Acceptability at 2 wk |
| Barnard et al,[16] 2016 | Premenopausal women with symptomatic uterine fibroids | 59 | 34 | Gynaecology | UAE vs MRgFUS^+ | Perioperative outcomes at 3 mo |
| Bergk, J. et al,[18] 2011 | Patients with DSM-IV disorder | 27 | 81 | Psychiatry | Mechanical restraint vs seclusion | CES at 4 wk |
| Boers et al,[19] 2017 | Pregnant women with disproportional intrauterine growth | 650 | 452 | Gynaecology | Induction vs expectative monitoring^ | (S)AE neonate at discharge |
| Brinkhaus et al,[20] 2017* | Patients with allergic asthma | 357 | 1088 | Social medicine | Acupuncture vs control^ | AQLQ at 3 mo |
| Brinkhaus et al,[21] 2008 | Patients with allergic rhinitis | 981 | 4256 | Social medicine | Acupuncture vs control^ | RQLQ at 30 d |
| Buhagiar et al, [22] 2017* | Patients after total knee arthroplasty | 165 | 87 | Orthopaedics | Home based vs inpatients recovery | Walking distance at 36 wk |
| Chekerov et al,[23] 2017 | Elderly with ovarian cancer receiving chemotherapy | 3 | 116 | Gynaecology | oral vs iv treosulfan | DFS at 2 y |
| Creutzig et al,[24] 2014 | Paediatric patients with relapsed AML | 101 | 54 | Haematology | L-DNR/Flag vs Flag | OS at 4 y |
| Crowther et al,[25] 2012 | Pregnant women with one prior caesarean | 22 | 2323 | Gynaecology | Caesarean vs vaginal birth^+ | Death and SAE at 30 d |
| Dalal et al,[26] 2006* | Participants in cardiac rehabilitation after acute MI | 104 | 126 | Cardiology | Home based vs hospital recovery | HAD at 9 mo |
| Ejlertsen et al,[27] 2008 | Premenopausal patients with breast cancer | 525 | 1628 | Oncology | Chemotherapy vs ovarian ablation^+ | DFS at 10 y |
| Erkan et al,[28] 2007 | Patients with positive aPL but no vascular and/or pregnancy events. | 98 | 74 | Internal medicine | Aspirin vs placebo or no aspirin^ | Acute thrombosis per 100-patients y |
| Fong et al,[29] 2015 | Patients with adolescent idiopathic scoliosis | 19 | 50 | Orthopedics | Brace vs observational^ | Recruitment feasibility |

For peer review only

| | | | | | | |
|---|---|---|---|---|---|---|
| Gall et al,[30] 2007 | Patients undergoing colon cancer surgery | 203 | 135 | Surgery | GP – vs surgeon follow-up | PCS score at 24 mo |
| Glazener et al,[31] 2016 | Patients with vaginal wall prolapse | 1348 | 1126 | Gynaecology | Mesh vs no mesh^+ | POPSS at 12 mo |
| Grant et al,[32] 2008* | Patients with gastro-oesophageal reflux disease | 357 | 453 | Upper GI | Surgery vs medication^+ | Reflux QOL at 1 y |
| Hatcher et al,[34] 2005 | Patients presenting with self-harm | 552 | 542 | Psychiatry | PST plus standard care vs standard care^ | Repeated self-harm at 1 y |
| Howard et al,[35] 2010* | Women requiring voluntary psychiatric admission | 42 | 61 | Psychiatry | crisis houses vs psychiatric wards | Functioning (GAF) at 12 wk |
| Hubacher et al,[36] 2017* | Women 18-29 years who were seeking a short -acting method | 382 | 524 | Gynaecology | long-acting vs short-acting contraceptive^ | Continuation rate at 1 y |
| Jones et al,[37] 2011* | Palliative cancer patients | 41 | 36 | Oncology | advance vs usual care^ | VAS (S) at 8 wk |
| Karlsen et al,[39] 2007 | Patients with proximal ureter stones | 50 | 21 | Urology | Shock wave vs ureteroscopy^+ | Stone free rate at 3 mo |
| Kearney et all,[40] 2011 | Patients with an acute Achilles tendon rupture | 20 | 29 | orthopedics | Surgery vs conservative^+ | Disability rating index at 9 mo |
| Kroz et all,[41] 2017 | Patients with breast cancer -related fatigue | 65 | 61 | Oncology | Multimodel combined program vs aerobic training^ | PSQI at 10 wk |
| Lock et al,[42] 2010 | Children with recurrent sore throats | 268 | 461 | Children Surgery | Surgery vs medication^+ | No. episodes of sore throats at 2 y |
| Majumdar et al,[43] 2010* | Patients with lower urinary tract symptoms (LUTS) | 99 | 210 | Urology | Urodynamics vs conservative^+ | Kings QOL at 6 mo |
| Mittal et al,[46] 2017* | Patients with type B ankle fracture | 160 | 276 | Orthopedics | Surgery vs no surgery^+ | FAOQ and PCI at 12 mo |
| Prescott et al,[49] 2007 | Women after breast conserving surgery | 255 | 100 | Oncology | Non- vs radiotherapy^ | QoL after 5 y |
| Purepong et al,[50] 2015* | Office workers suffering from low back pain (LBP) | 64 | 37 | Physical therapy | Backrest vs no intervention^ | VAS at 3 mo |
| Raue et al,[52] 2011 | Patients operated for diverticulitis | 149 | 294 | Surgery | Laparoscopic vs open approach | QoL at 30 d |
| Robson et al,[53] 2009* | Termination of pregnancy less than 14 weeks gestation | 349 | 1528 | Gynaecology | Medicine vs surgery TOP^+ | Acceptability TOP at 2 wk |

For peer review only

| | | | | | | |
|---|---|---|---|---|---|---|
| Schweikert et al,[55] 2009 | Patient for cardiac rehabilitation | 4 | 163 | Cardiology | Out-patient vs in-patient recovery | EQ-5D at 12 mo |
| Shi guang et al,[58] 2014* | Patients with vascular dementia | 48 | 20 | Alternative medicine | Acupuncture vs training^ | SDSVD at |
| Sinclair et al,[59] 2017* | Patients with severe lung disease | 67 | 82 | Pulmonology | Advance care planning vs standard | ACP uptake at 6 mo |
| Schwieger et al,[56] 2016* | Adolescent with idiopathic scoliosis (AIS) | 132 | 187 | Orthopaedics | Brace vs observation^ | QOL at 2 y |
| Underwood et al,[60] 2008* | Older patients with chronic knee pain | 282 | 303 | Orthopaedics | Topic vs oral ibuprofen | WOMAC at 12 mo |
| van der Kooij et al,[62] 2013 | Uterine fibroids | 177 | 103 | Gynaecology | Embolization vs hysterectomy^+ | HRQoL at 12 mo |
| Van Heest et al,[63] 2015 | Children with upper extremity cerebral palsy | 29 | 10 | Orthopedics | Surgery vs botuline therapy^+ | SHUEE at 24 wk |
| Weinstein et al,[65] 2006* | Patients with spondylolisthesis | 304 | 303 | Orthopaedics | Surgical vs non-surgical^+ | Physical functioning (SF-36 Phys) at 2 y |
| Weinstein et al,[64] 2008* | Patients with spinal stenosis | 289 | 365 | Orthopaedics | Surgical vs non-surgical^+ | Physical functioning (SF-36 Phys) at 2 y |
| Witbrodt,[67] 2007* | addicted people | 293 | 321 | Social medicine | Community residential vs day hospital^ | Abstinence at 12 mo |
| Witt el al,[68] 2006* | Patients with chronic low back pain | 2841 | 8537 | Rheumatology | Acupuncture vs control^ | HFAQ at 3 mo |
| Witt et al,[69] 2006* | Patients with osteoarthritis | 712 | 2921 | Rheumatology | Acupuncture vs control^ | Osteoarthritis index (WOMAC) at 3 mo |
| Woodward et al,[72] 2004 | Pregnant women | 60 | 20 | Gynaecology | Water- vs land birth | Baby condition at 6 wk |

*These 20 trials could be used to calculate standardised effect sizes of the randomised- and preference cohort separately, and were included in our reanalysis on the effect of preference on outcome. ^These 32 trials compared interventions versus conservative treatment. +These 16 trials compared surgical interventions versus conservative treatment.

Abbreviations: Wk, week; mo, months; y, year; MRgFUS, magnetic resonance imaging-guided focused ultrasound surgery; UAE, uterine artery embolization; HRQoL, Health related Quality of Life; CES, Coercion Experience Scale; WOMAC, Western Ontario and McMaster Universities Osteoarthritis Index; HFAQ, Hannover Functional Ability Questionnaire; AQLQ, Astma Quality of Life; SAE, Serious adverse event; HAD, Hospital Anxiety Depression scale; GAF, Global assessment of functioning; BPRS, Brief psychiatric rating scale; VAS, Visual analogue scales; FAOQ, Foot and Ankle outcomes questionnaire; PCI, Physical component score; RMDQ, Roland-Morris Disability Questionnaire; TOP, Termination of pregnancy; SVSVD, Scale of differentiation of syndromes of vascular dementia; ACP, Advance care planning; DFS, disease free survival; OS, overall survival; PCS, peritoneal cancer score; PST, problem solving therapy; RQLQ,

For peer review only

Rhinitis Quality of life questionnaire; L-DNR, liposomal daunorubicin; FLAG, fludarabut; POPSS, Pelvic organ prolapse symptom score; SHUEE, Shriners Hospital Upper Extremity Evaluation; SF-36 Phys, short-from 36 scale physical functioning; PSQI, Pittsburg sleep Quality index; R, randomised; P, preference.

For peer review only

## References


1 Effectiveness. NC for R and DU systematic reviews of research on. Centre for Reviews and Disseminationt's Guidance for Those Carrying Out or Commissioning Reveiws. *York, Engl Univ York* 2001;**Report 4**.

2 Walter SD, Turner RM, Macaskill P, *et al.* Estimation of treatment preference effects in clinical trials when some participants are indifferent to treatment choice. *BMC Med Res Methodol* 2017;**17**:29. doi:10.1186/s12874-017-0304-x

3 Brewin CR, Bradley C. Patient preferences and randomised clinical trials. *Br Med J* 1989;**299**:313–5. doi:10.1136/bmj.299.6694.313

4 King M, Nazareth I, Lampe F, *et al.* Impact of Participant and Physician Intervention Preferences on Randomized Trials. *JAMA* 2005;**293**:1089. doi:10.1001/jama.293.9.1089

5 Swift JK, Callahan JL. The impact of client treatment preferences on outcome: a meta-analysis. *J Clin Psychol* 2009;**65**:368–81. doi:10.1002/jclp.20553

6 Oxford Centre for Evidence-based Medicine - Levels of Evidence (March 2009) - CEBM. https://www.cebm.net/2009/06/oxford-centre-evidence-based-medicine-levels-evidence-march-2009/ (accessed 3 Jul 2019).

7 Higgins JPT GS (Eds. . Cochrane handbook for systematic reviews of interventions. Version 5.1.0. *Cochrane Collab Oxford;* Published Online First: 2011.http://handbook-5-1.cochrane.org/

8 Moher D, Liberati A, Tetzlaff J, *et al.* Preferred reporting items for systematic reviews and meta-analyses: the PRISMA statement. *BMJ* 2009;**339**:b2535–b2535. doi:10.1136/bmj.b2535

9 Sterne JA, Hernán MA, Reeves BC, *et al.* ROBINS-I: a tool for assessing risk of bias in non-randomised studies of interventions. *BMJ* 2016;**355**:i4919. doi:10.1136/bmj.i4919

10 Higgins JPT, Altman DG, Gotzsche PC, *et al.* The Cochrane Collaboration's tool for assessing risk of bias in randomised trials. *BMJ* 2011;**343**:d5928–d5928. doi:10.1136/bmj.d5928

11 Guyatt GH, Ebrahim S, Alonso-Coello P, *et al.* GRADE guidelines 17: assessing the risk of bias associated with missing participant outcome data in a body of evidence. *J Clin Epidemiol* 2017;**87**:14–22. doi:10.1016/j.jclinepi.2017.05.005

12 Wan X, Wang W, Liu J, *et al.* Estimating the sample mean and standard deviation from the sample size, median, range and/or interquartile range. *BMC Med Res Methodol* 2014;**14**:135. doi:10.1186/1471-2288-14-135

13 MW Lipsey DW. Practical meta-analysis. *Thousan Oaks, Calif Sage Publ* 2001.

14 16.1.3.2 Imputing standard deviations for changes from baseline. http://handbook-5-1.cochrane.org/chapter_16/16_1_3_2_imputing_standard_deviations_for_changes_from_baseline.htm (accessed 10 Jul 2018).

15 Ashok PW, Hamoda H, Flett GM, *et al.* Patient preference in a randomized study comparing medical and surgical abortion at 10-13 weeks gestation. *Contraception* 2005;**71**:143–8. doi:10.1016/j.contraception.2004.08.013

16 Barnard EP, AbdElmagied AM, Vaughan LE, *et al.* Periprocedural outcomes comparing fibroid embolization and focused ultrasound: a randomized controlled trial and comprehensive cohort analysis. *Am J Obstet Gynecol* 2017;**216**:500.e1-500.e11. doi:10.1016/j.ajog.2016.12.177


For peer review only

17 Barnestein-Fonseca P, Vazquez-Alarcon R, Leiva-Fernandez F, *et al.* Inhalation Technique Evolution After Training in Copd. The Role of the Device. *Value Heal* 2014;**17**:A600. doi:10.1016/j.jval.2014.08.2076

18 Bergk J, Einsiedler B, Flammer E, *et al.* A randomized controlled comparison of seclusion and mechanical restraint in inpatient settings. *Psychiatr Serv* 2011;**62**:1310–7. doi:10.1176/ps.62.11.pss6211_1310

19 KE Boers, L van Wyk, JAM van der Post, A Kwee, MG van Pampus, HA Bremer, FMC Delemarre, KWM Bloemenkamp, S le Cessie, FME Roumen, JG Thornton, JMM van Lith, BW J Mol SS. *Comparison of participants and non-participnatns in a trial of induction of labour versus expectant monitoring for intrauterine growth restriction at term (teh DIGITAT trial); a prospective cohort study.* 2012.https://openaccess.leidenuniv.nl/bitstream/handle/1887/18948/06.pdf?sequence=12

20 Brinkhaus B, Roll S, Jena S, *et al.* Acupuncture in Patients with Allergic Asthma: A Randomized Pragmatic Trial. *J Altern Complement Med* 2017;**23**:268–77. doi:10.1089/acm.2016.0357

21 Brinkhaus B, Witt CM, Jena S, *et al.* Acupuncture in patients with allergic rhinitis: a pragmatic randomized trial. *Ann Allergy, Asthma Immunol* 2008;**101**:535–43.http://search.ebscohost.com/login.aspx?direct=true&db=rzh&AN=105590802&lang=nl&site=ehost-live&scope=site

22 Buhagiar MA, Naylor JM, Harris IA, *et al.* Effect of Inpatient Rehabilitation vs a Monitored Home-Based Program on Mobility in Patients With Total Knee Arthroplasty: The HIHO Randomized Clinical Trial. *JAMA J Am Med Assoc* 2017;**317**:1037–46. doi:10.1001/jama.2017.1224

23 Chekerov R, Harter P, Fuxius S, *et al.* Preference of elderly patients' to oral or intravenous chemotherapy in heavily pre-treated recurrent ovarian cancer: final results of a prospective multicenter trial. *Gynecol Oncol Res Pract* 2017;**4**:6. doi:10.1186/s40661-017-0040-2

24 Creutzig U, Semmler J, Kaspers GL, *et al.* Re-induction with L-DNR/FLAG improves response after AML relapse, but not long-term survival. *Klin Padiatr* 2014;**226**:323–31. doi:10.1055/s-0034-1385918

25 Crowther CA, Dodd JM, Hiller JE, *et al.* Planned Vaginal Birth or Elective Repeat Caesarean: Patient Preference Restricted Cohort with Nested Randomised Trial. *PLoS Med* 2012;**9**:e1001192. doi:10.1371/journal.pmed.1001192

26 Dalal HM, Evans PH, Campbell JL, *et al.* Home-based versus hospital-based rehabilitation after myocardial infarction: A randomized trial with preference arms--Cornwall Heart Attack Rehabilitation Management Study (CHARMS). *Int J Cardiol* 2007;**119**:202–11. doi:10.1016/j.ijcard.2006.11.018

27 Ejlertsen B, Jensen M-B, Mouridsen HT, *et al.* DBCG trial 89B comparing adjuvant CMF and ovarian ablation: similar outcome for eligible but non-enrolled and randomized breast cancer patients. *Acta Oncol* 2008;**47**:709–17. doi:10.1080/02841860802001475

28 Erkan D, Harrison MJ, Levy R, *et al.* Aspirin for primary thrombosis prevention in the antiphospholipid syndrome: a randomized, double-blind, placebo-controlled trial in asymptomatic antiphospholipid antibody-positive individuals. *Arthritis Rheum* 2007;**56**:2382–91.http://search.ebscohost.com/login.aspx?direct=true&db=rzh&AN=105979335&lang=nl&site=ehost-live&scope=site

29 Fong DYT, Cheung KMC, Wong YW, *et al.* An alternative to a randomised control design for assessing the efficacy and effectiveness of bracing in adolescent idiopathic scoliosis. *Bone Joint J* 2015;**97–B**:973–81. doi:10.1302/0301-620X.97B7.35147

30 Gall CA, Weller D, Esterman A, *et al.* Patient satisfaction and health-related quality of life after

For peer review only

treatment for colon cancer. *Dis Colon Rectum* 2007;**50**:801–9. doi:10.1007/s10350-006-0815-8

31 Glazener C, Breeman S, Elders A, *et al.* Clinical effectiveness and cost-effectiveness of surgical options for the management of anterior and/or posterior vaginal wall prolapse: two randomised controlled trials within a comprehensive cohort study – results from the PROSPECT Study. *Health Technol Assess (Rockv)* 2016;**20**:1–452. doi:10.3310/hta20950

32 Grant A, Boachie C, Cotton S, *et al.* Clinical and economic evaluation of laparoscopic surgery compared with medical management for gastro-oesophageal reflux disease: 5-year follow-up of multicentre randomised trial (the REFLUX trial). *Heal Technol Assess* 2013;**17**:1–167. doi:10.3310/hta17220

33 Hartley S, Haddock G. Self-help therapy and recovery in psychosis: methodological considerations and service user involvement in a partially randomised preference trial. *Trials* 2011;**12**:A84. doi:10.1186/1745-6215-12-S1-A84

34 Hatcher S, Sharon C, Parag V, *et al.* Problem-solving therapy for people who present to hospital with self-harm: Zelen randomised controlled trial. *Br J Psychiatry* 2011;**199**:310–6. doi:10.1192/bjp.bp.110.090126

35 Howard L, Flach C, Leese M, *et al.* Effectiveness and cost-effectiveness of admissions to women's crisis houses compared with traditional psychiatric wards: pilot patient-preference randomised controlled trial. *Br J Psychiatry Suppl* 2010;**53**:s32-40. doi:10.1192/bjp.bp.110.081083

36 Hubacher D, Spector H, Monteith C, *et al.* Long-acting reversible contraceptive acceptability and unintended pregnancy among women presenting for short-acting methods: a randomized patient preference trial. *Am J Obstet Gynecol* 2017;**216**:101–9. doi:10.1016/j.ajog.2016.08.033

37 Jones L, Harrington J, Barlow CA, *et al.* Advance care planning in advanced cancer: can it be achieved? An exploratory randomized patient preference trial of a care planning discussion. *Palliat Support Care* 2011;**9**:3–13. doi:10.1017/S1478951510000490

38 Karidakis GK, Karachalios T. Oxidized Zirconium Head on Crosslinked Polyethylene Liner in Total Hip Arthroplasty: A 7- to 12-year In Vivo Comparative Wear Study. *Clin Orthop Relat Res* 2015;**473**:3836–45. doi:10.1007/s11999-015-4503-7

39 Karlsen SJ, Renkel J, Tahir AR, *et al.* Extracorporeal shockwave lithotripsy versus ureteroscopy for 5- to 10-mm stones in the proximal ureter: Prospective effectiveness patient-preference trial. *J Endourol* 2007;**21**:28–33. doi:10.1089/end.2006.0153

40 Kearney RS, Achten J, Parsons NR, *et al.* The comprehensive cohort model in a pilot trial in orthopaedic trauma. *BMC Med Res Methodol* 2011;**11**:39. doi:10.1186/1471-2288-11-39

41 Kröz M, Reif M, Glinz A, *et al.* Impact of a combined multimodal-aerobic and multimodal intervention compared to standard aerobic treatment in breast cancer survivors with chronic cancer-related fatigue - results of a three-armed pragmatic trial in a comprehensive cohort design. *BMC Cancer* 2017;**17**:166. doi:10.1186/s12885-017-3142-7

42 Lock C, Wilson, J, Steen N, *et al.* Comparison of case note review methods for evaluating quality and safety in health care. *Health Technol Assess (Rockv)* 2010;**14**:1–164, iii–iv. doi:10.3310/hta14130

43 Majumdar A, Latthe P, Toozs-Hobson P. Urodynamics prior to treatment as an intervention: A pilot study. *Neurourol Urodyn* 2009;:n/a-n/a. doi:10.1002/nau.20810

44 Mills N, Metcalfe C, Ronsmans C, *et al.* A comparison of socio-demographic and psychological factors between patients consenting to randomisation and those selecting treatment (the ProtecT study). *Contemp Clin Trials* 2006;**27**:413–9. doi:10.1016/j.cct.2006.04.008

45 Mitchell-Jones N, Farren JA, Tobias A, *et al.* Ambulatory versus inpatient management of

For peer review only

severe nausea and vomiting of pregnancy: a randomised control trial with patient preference arm. *BMJ Open* 2017;**7**:e017566. doi:10.1136/bmjopen-2017-017566

46 Mittal R, Harris IA, Adie S, *et al.* Surgery for Type B Ankle Fracture Treatment: a Combined Randomised and Observational Study (CROSSBAT). *BMJ Open* 2017;**7**:e013298. doi:10.1136/bmjopen-2016-013298

47 Narasimmaraj P, Stover Fiscalini A, Kaplan C, *et al.* Abstract P3-10-01: A pilot feasibility study of the WISDOM study, a preference-tolerant randomized controlled trial evaluating a risk-based breast cancer screening strategy. *Cancer Res* 2016;**76**:P3-10-01-P3-10-01. doi:10.1158/1538-7445.SABCS15-P3-10-01

48 Nozaki I, Kato K, Igaki H, *et al.* Evaluation of safety profile of thoracoscopic esophagectomy for T1bN0M0 cancer using data from JCOG0502: a prospective multicenter study. *Surg Endosc* 2015;**29**:3519–26. doi:10.1007/s00464-015-4102-4

49 Prescott RJ, Kunkler IH, Williams LJ, *et al.* A randomised controlled trial of postoperative radiotherapy following breast-conserving surgery in a minimum-risk older population. The PRIME trial. *Health Technol Assess* 2007;**11**:1–149, iii–iv.http://www.ncbi.nlm.nih.gov/pubmed/17669280 (accessed 8 May 2018).

50 Purepong N, Channak S, Boonyong S, *et al.* The effect of an acupressure backrest on pain and disability in office workers with chronic low back pain: A randomized, controlled study and patients' preferences. *Complement Ther Med* 2015;**23**:347–55. doi:10.1016/j.ctim.2015.03.005

51 Ramanathan K, Ioannou K, Keshmiri H, *et al.* 326 Disparity Between Clinical Trials and Registry Outcomes: Reflections From the Bypass Angioplasty Revascularization Investigation 2 Diabetes (BARI-2D) Trial. *Can J Cardiol* 2012;**28**:S218–9. doi:10.1016/j.cjca.2012.07.307

52 Raue W, Langelotz C, Paolucci V, *et al.* Problems of randomization to open or laparoscopic sigmoidectomy for diverticular disease. *Int J Colorectal Dis* 2011;**26**:369–75. doi:10.1007/s00384-010-1074-7

53 Robson S, Kelly T, Howel, D, *et al.* Randomised preference trial of medical versus surgical termination of pregnancy less than 14 weeks' gestation (TOPS). *Health Technol Assess (Rockv)* 2009;**13**. doi:10.3310/hta13530

54 Ryan M, Nitsun M, Gilbert L, *et al.* A prospective study of the effectiveness of group and individual psychotherapy for women CSA survivors. *Psychol Psychother* 2005;**78**:465–79. doi:10.1348/147608305X42226

55 Schweikert B, Hahmann H, Steinacker JM, *et al.* Intervention study shows outpatient cardiac rehabilitation to be economically at least as attractive as inpatient rehabilitation. *Clin Res Cardiol* 2009;**98**:787–95. doi:10.1007/s00392-009-0081-6

56 Schwieger T, Campo S, Weinstein SL, *et al.* Body Image and Quality-of-Life in Untreated Versus Brace-Treated Females With Adolescent Idiopathic Scoliosis. *Spine (Phila Pa 1976)* 2016;**41**:311–9. doi:10.1097/brs.0000000000001210

57 Shavelle D, Kapasi N, Banerjee S, *et al.* TCT-127 Prior Coronary Artery Bypass Graft Surgery and Hemodynamically Supported High Risk Percutaneous Coronary Intervention: Observations from The PROTECT II Randomized Trial and The cVAD Registry. *J Am Coll Cardiol* 2016;**68**:B51. doi:10.1016/j.jacc.2016.09.033

58 Shi GX, Liu CZ, Guan W, *et al.* Effects of acupuncture on Chinese medicine syndromes of vascular dementia. *Chin J Integr Med* 2014;**20**:661–6. doi:10.1007/s11655-013-1323-4

59 Sinclair C, Auret KA, Evans SF, *et al.* Advance care planning uptake among patients with severe lung disease: A randomised patient preference trial of a nurse-led, facilitated advance care planning intervention. *BMJ Open* 2017;**7**:e013415. doi:10.1136/bmjopen-2016-013415

For peer review only

60 Underwood M, Ashby D, Cross P, *et al.* Advice to use topical or oral ibuprofen for chronic knee pain in older people: randomised controlled trial and patient preference study. *BMJ* 2008;**336**:138–42. doi:10.1136/bmj.39399.656331.25

61 Impact of Vitamin D on Insulin Resistance... (PDF Download Available). https://www.researchgate.net/publication/264216208_Impact_of_Vitamin_D_on_Insulin_Resistance_in_Patients_with_Type_II_Diabetes_A_Comprehensive_Cohort_Design (accessed 8 May 2018).

62 van der Kooij SM, Hehenkamp WJK, Birnie E, *et al.* The effect of treatment preference and treatment allocation on patients' health-related quality of life in the randomized EMMY trial. *Eur J Obstet Gynecol Reprod Biol* 2013;**169**:69–74. doi:10.1016/j.ejogrb.2013.01.019

63 Van Heest AE, Bagley A, Molitor F, *et al.* Tendon transfer surgery in upper-extremity cerebral palsy is more effective than botulinum toxin injections or regular, ongoing therapy. *J Bone Jt Surg Am* 2015;**97**:529–36. doi:10.2106/jbjs.m.01577

64 Weinstein JN, Tosteson TD, Lurie JD, *et al.* Surgical versus Nonsurgical Therapy for Lumbar Spinal Stenosis. *N Engl J Med* 2008;**358**:794–810. doi:10.1056/NEJMoa0707136

65 Weinstein JN, Lurie JD, Tosteson TD, *et al.* Surgical versus nonsurgical treatment for lumbar degenerative spondylolisthesis. *N Engl J Med* 2007;**356**:2257–70. doi:10.1056/NEJMoa070302

66 Wiegel T, Stöckle M, Bartkowiak D. PREFEREnce-based Randomized Evaluation of Treatment Modalities in Low or Early Intermediate-risk Prostate Cancer. *Eur Urol* 2015;**67**:1–2. doi:10.1016/j.eururo.2014.09.016

67 Witbrodt J, Bond J, Kaskutas LA, *et al.* Day hospital and residential addiction treatment: randomized and nonrandomized managed care clients. *J Consult Clin Psychol* 2007;**75**:947–59. doi:10.1037/0022-006x.75.6.947

68 Witt CM, Jena S, Selim D, *et al.* Pragmatic randomized trial evaluating the clinical and economic effectiveness of acupuncture for chronic low back pain. *Am J Epidemiol* 2006;**164**:487–96. doi:aje/kwj224

69 Witt CM, Jena S, Brinkhaus B, *et al.* Acupuncture in patients with osteoarthritis of the knee or hip: a randomized, controlled trial with an additional nonrandomized arm. *Arthritis Rheum* 2006;**54**:3485–93. doi:10.1002/art.22154

70 C.G. Wood, P. Srivastava, L. Lacombe, A.I. Gorelov, S. Gorelov, P. Mulders, H. Zielinski, F. Teofilovici, L. Isakov BE. Survival update from a multicenter, randomized, phase III trial of vitespen versus observation as adjuvant therapy for renal cell carcinoma in patients at high risk of recurrence. *J Clin Oncol* 2009;**27**:3009–3009. doi:10.1200/jco.2009.27.15s.3009 Journal of Clinical Oncology 27, no. 15S (May 20 2009) 3009-3009.

71 Yoneda T, Shoji K, Takase H, *et al.* Effectiveness and safety of 1-year ad libitum consumption of a high-catechin beverage under nutritional guidance. *Metab Syndr Relat Disord* 2009;**7**:349–56. doi:10.1089/met.2008.0061

72 Woodward J, Kelly SM. A pilot study for a randomised controlled trial of waterbirth versus land birth. *BJOG* 2004;**111**:537–45. doi:10.1111/j.1471-0528.2004.00132.x

73 Rothwell PM. External validity of randomised controlled trials: "To whom do the results of this trial apply?" *Lancet* 2005;**365**:82–93. doi:10.1016/S0140-6736(04)17670-8

74 Bothwell LE, Greene JA, Podolsky SH, *et al.* Assessing the Gold Standard — Lessons from the History of RCTs. *N Engl J Med* 2016;**374**:2175–81. doi:10.1056/NEJMms1604593

75 Chalmers TC, Celano P, Sacks HS, *et al.* Bias in treatment assignment in controlled clinical trials. *N Engl J Med* 1983;**309**:1358–61. doi:10.1056/NEJM198312013092204


For peer review only

76 Colditz GA, Miller JN, Mosteller F. How study design affects outcomes in comparisons of therapy. I: Medical. *Stat Med* 1989;**8**:441–54.http://www.ncbi.nlm.nih.gov/pubmed/2727468 (accessed 7 Aug 2018).

77 Sackett DL. *Evidence-based medicine : how to practice and teach EBM*. Churchill Livingstone 2000. https://books.google.nl/books?hl=nl&id=Qh1ntQEACAAJ&focus=searchwithinvolume&q=stop+reading (accessed 7 Aug 2018).

78 Benson K, Hartz AJ. A Comparison of Observational Studies and Randomized, Controlled Trials. *N Engl J Med* 2000;**342**:1878–86. doi:10.1056/NEJM200006223422506

79 Concato J, Shah N, Horwitz RI. Randomized, controlled trials, observational studies, and the hierarchy of research designs. *N Engl J Med* 2000;**342**:1887–92. doi:10.1056/NEJM200006223422507

80 Murthy VH, Krumholz HM, Gross CP. Participation in Cancer Clinical Trials. *JAMA* 2004;**291**:2720. doi:10.1001/jama.291.22.2720

For peer review only

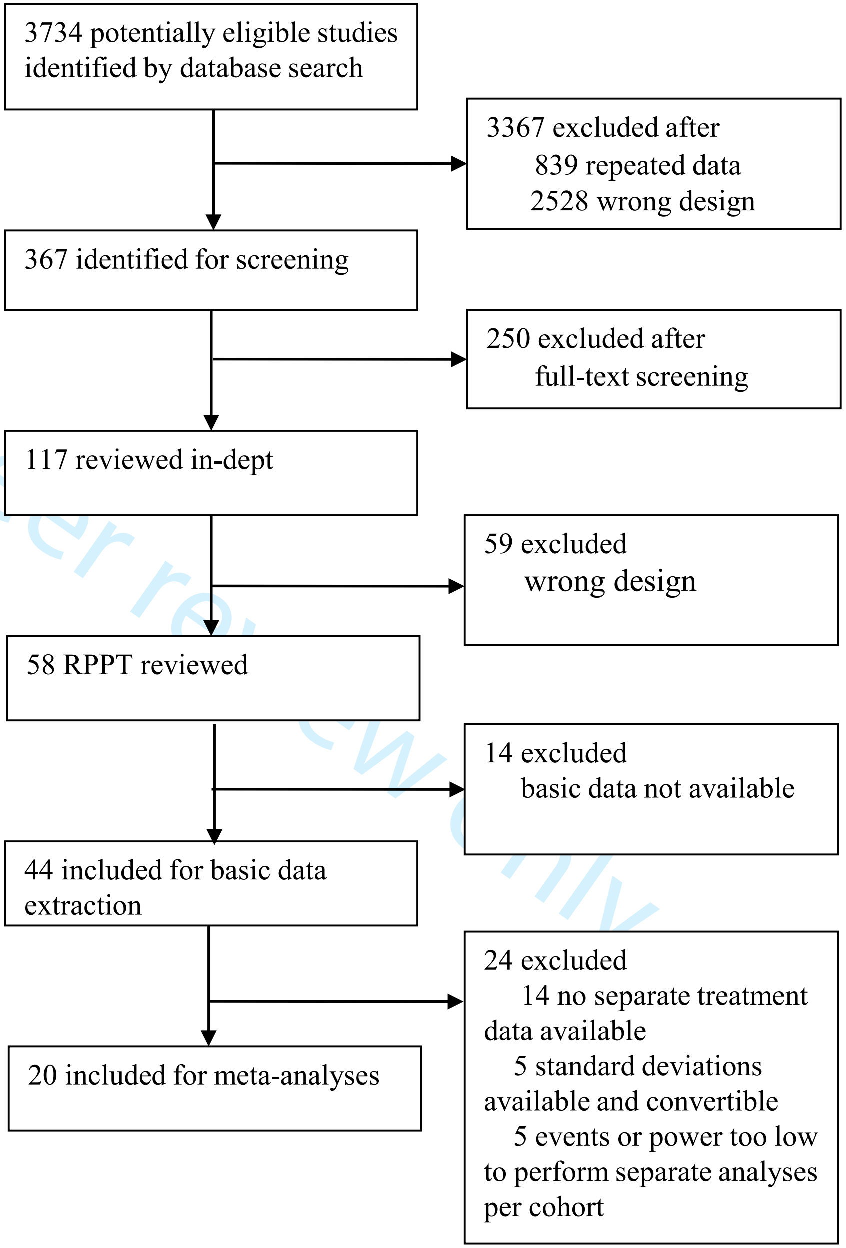


For peer review only - http://bmjopen.bmj.com/site/about/guidelines.xhtml

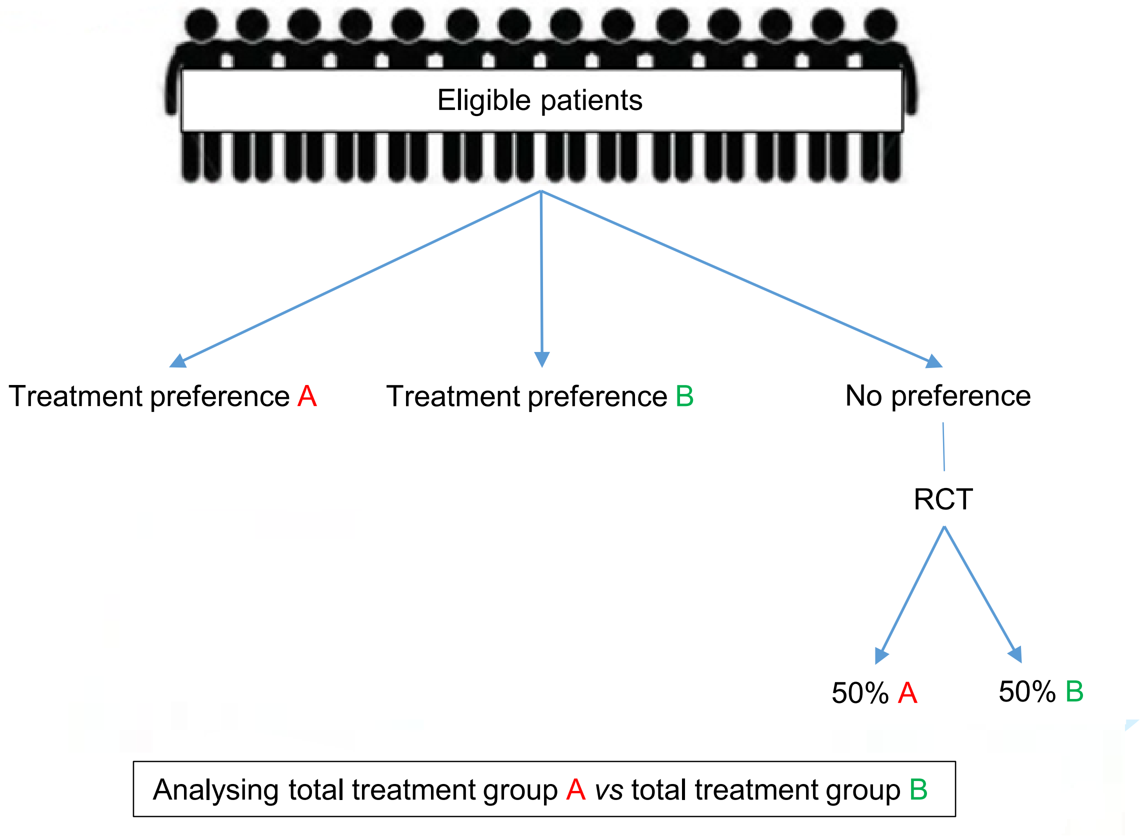
Eligible patients
Treatment preference A
Treatment preference B
No preference
RCT
50% A
50% B
Analysing total treatment group A vs total treatment group B

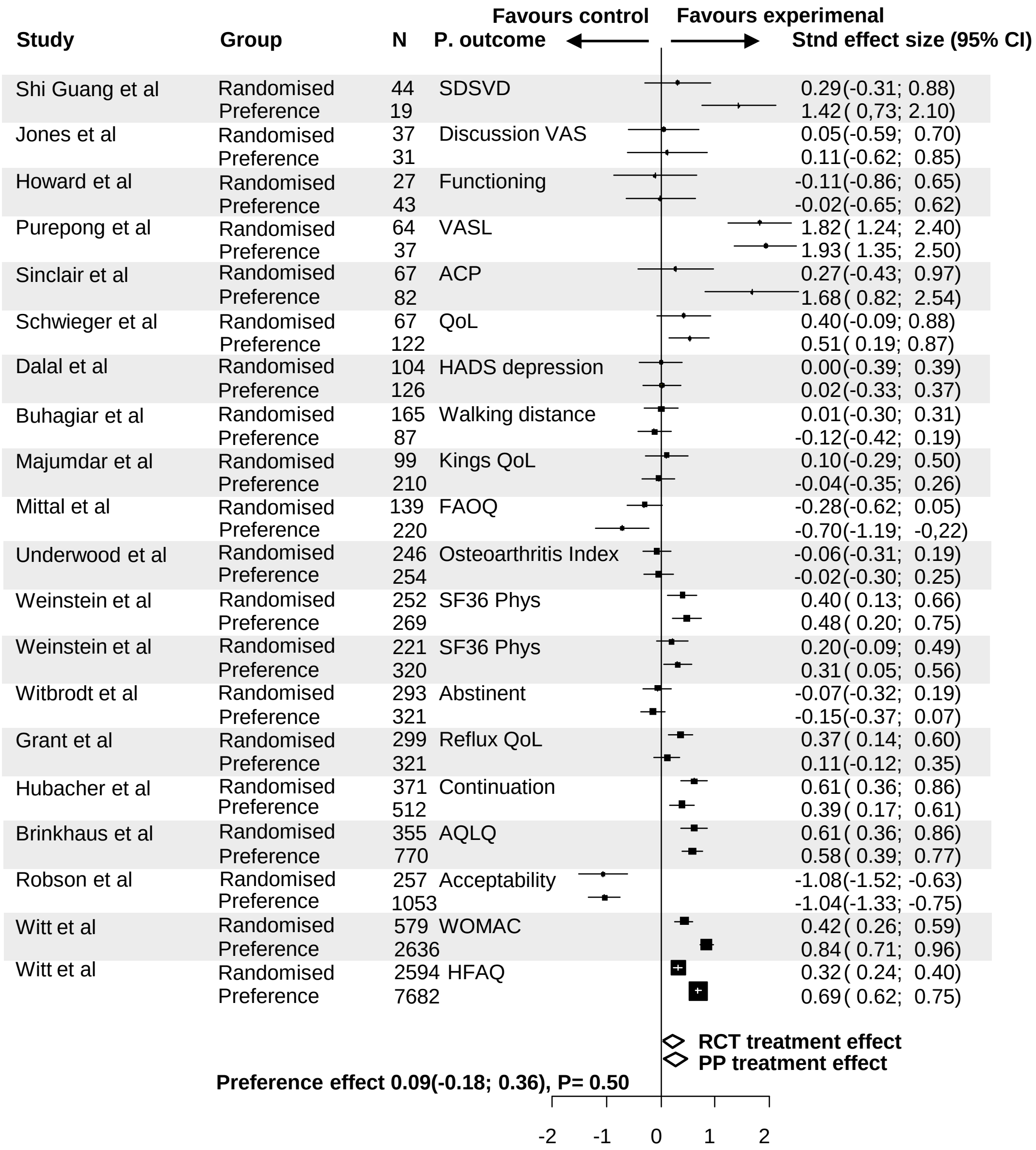
Favours control
Favours experimenal
Study
Group
N
P. outcome
Stnd effect size (95% CI)
Shi Guang et al Randomised 44 SDSVD 0.29(-0.31; 0.88)
Preference 19 1.42( 0,73; 2.10)
Jones et al Randomised 37 Discussion VAS 0.05(-0.59; 0.70)
Preference 31 0.11(-0.62; 0.85)
Howard et al Randomised 27 Functioning -0.11(-0.86; 0.65)
Preference 43 -0.02(-0.65; 0.62)
Purepong et al Randomised 64 VASL 1.82( 1.24; 2.40)
Preference 37 1.93( 1.35; 2.50)
Sinclair et al Randomised 67 ACP 0.27(-0.43; 0.97)
Preference 82 1.68( 0.82; 2.54)
Schwieger et al Randomised 67 QoL 0.40(-0.09; 0.88)
Preference 122 0.51( 0.19; 0.87)
Dalal et al Randomised 104 HADS depression 0.00(-0.39; 0.39)
Preference 126 0.02(-0.33; 0.37)
Buhagiar et al Randomised 165 Walking distance 0.01(-0.30; 0.31)
Preference 87 -0.12(-0.42; 0.19)
Majumdar et al Randomised 99 Kings QoL 0.10(-0.29; 0.50)
Preference 210 -0.04(-0.35; 0.26)
Mittal et al Randomised 139 FAOQ -0.28(-0.62; 0.05)
Preference 220 -0.70(-1.19; -0,22)
Underwood et al Randomised 246 Osteoarthritis Index -0.06(-0.31; 0.19)
Preference 254 -0.02(-0.30; 0.25)
Weinstein et al Randomised 252 SF36 Phys 0.40( 0.13; 0.66)
Preference 269 0.48( 0.20; 0.75)
Weinstein et al Randomised 221 SF36 Phys 0.20(-0.09; 0.49)
Preference 320 0.31( 0.05; 0.56)
Witbrodt et al Randomised 293 Abstinent -0.07(-0.32; 0.19)
Preference 321 -0.15(-0.37; 0.07)
Grant et al Randomised 299 Reflux QoL 0.37( 0.14; 0.60)
Preference 321 0.11(-0.12; 0.35)
Hubacher et al Randomised 371 Continuation 0.61( 0.36; 0.86)
Preference 512 0.39( 0.17; 0.61)
Brinkhaus et al Randomised 355 AQLQ 0.61( 0.36; 0.86)
Preference 770 0.58( 0.39; 0.77)
Robson et al Randomised 257 Acceptability -1.08(-1.52; -0.63)
Preference 1053 -1.04(-1.33; -0.75)
Witt et al Randomised 579 WOMAC 0.42( 0.26; 0.59)
Preference 2636 0.84( 0.71; 0.96)
Witt et al Randomised 2594 HFAQ 0.32( 0.24; 0.40)
Preference 7682 0.69( 0.62; 0.75)
RCT treatment effect
PP treatment effect
Preference effect 0.09(-0.18; 0.36), P= 0.50
-2 -1 0 1 2

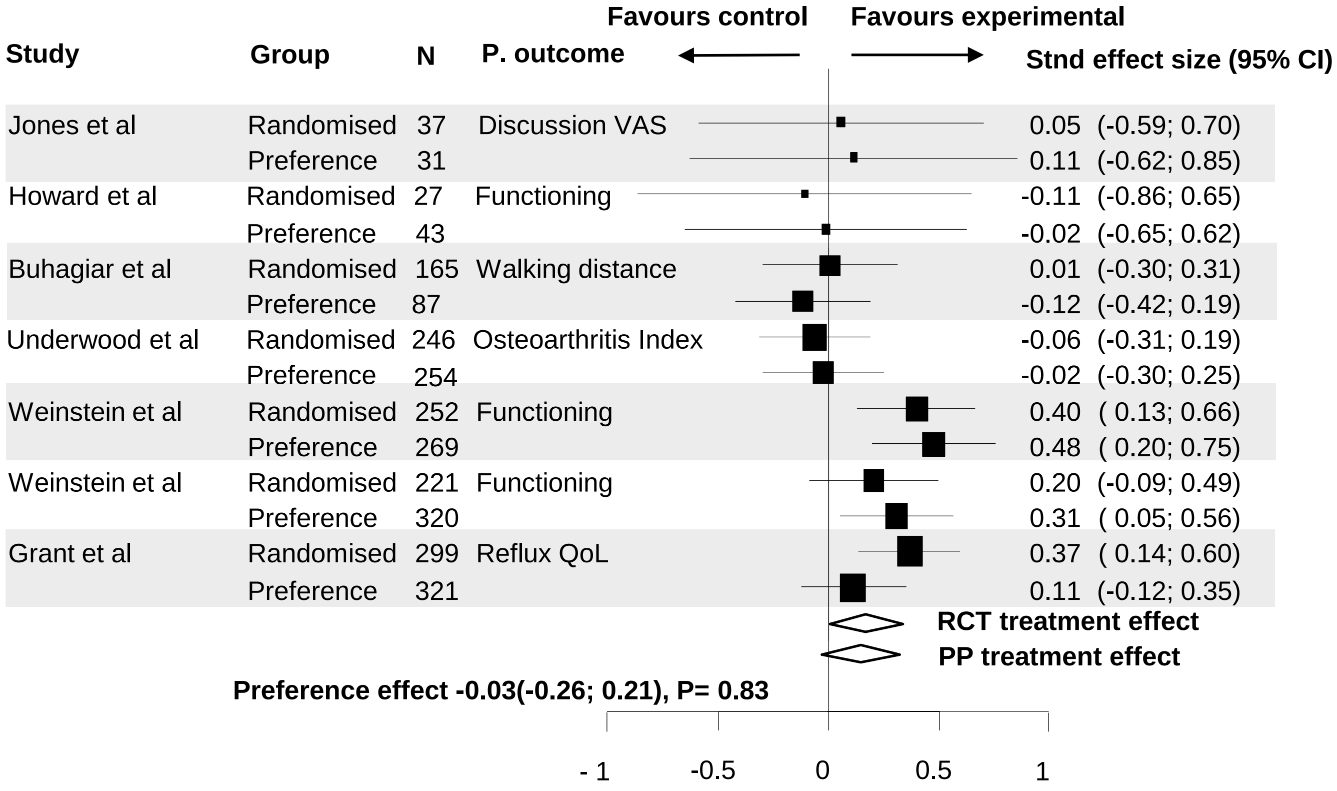


For peer review only - http://bmjopen.bmj.com/site/about/guidelines.xhtml

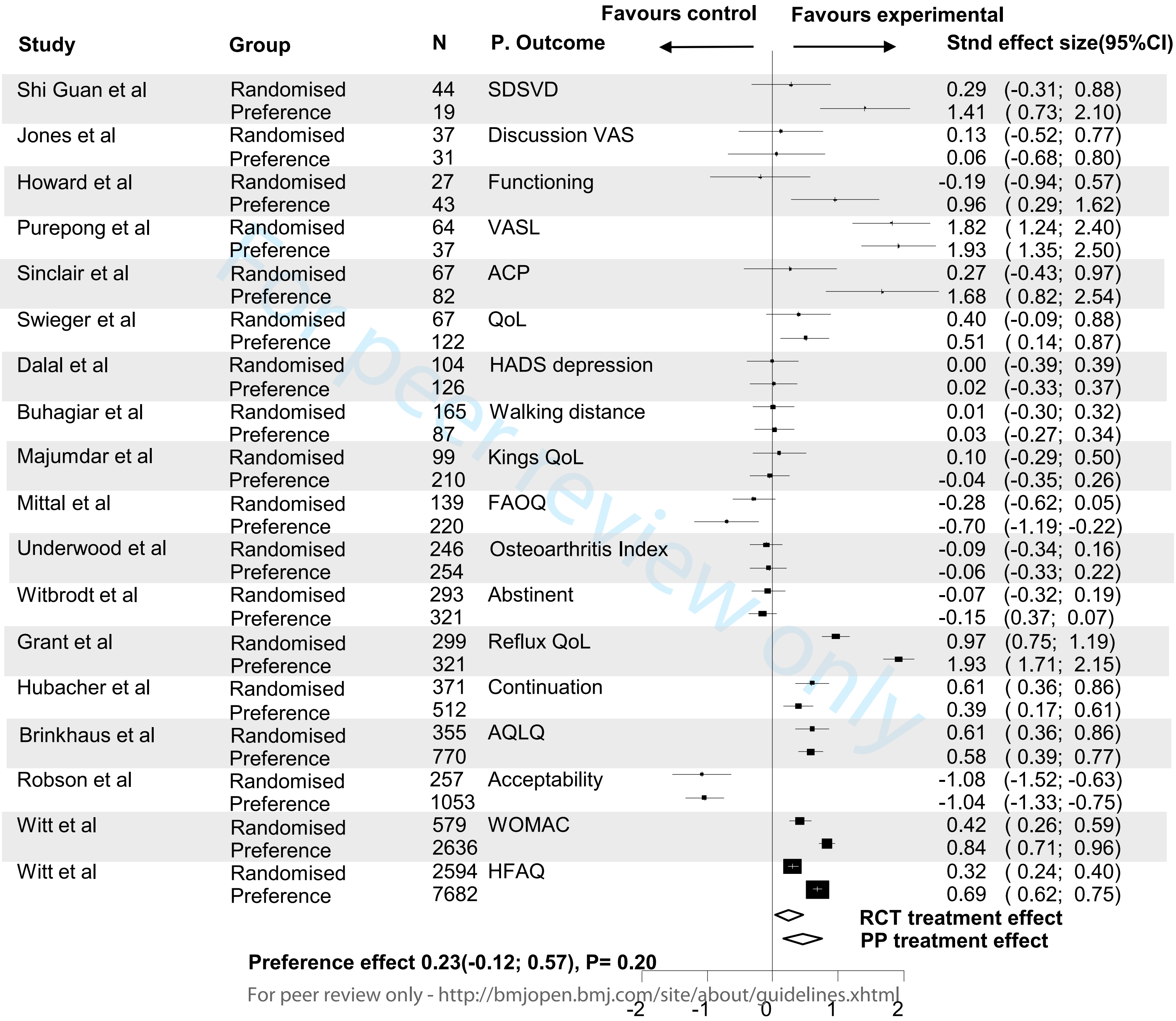
Favours control
Favours experimental
Study | Group | N | P. Outcome | Stnd effect size(95%CI)
Shi Guan et al | Randomised | 44 | SDSVD | 0.29 (-0.31; 0.88)
Preference | 19 | 1.41 ( 0.73; 2.10)
Jones et al | Randomised | 37 | Discussion VAS | 0.13 (-0.52; 0.77)
Preference | 31 | 0.06 (-0.68; 0.80)
Howard et al | Randomised | 27 | Functioning | -0.19 (-0.94; 0.57)
Preference | 43 | 0.96 ( 0.29; 1.62)
Purepong et al | Randomised | 64 | VASL | 1.82 ( 1.24; 2.40)
Preference | 37 | 1.93 ( 1.35; 2.50)
Sinclair et al | Randomised | 67 | ACP | 0.27 (-0.43; 0.97)
Preference | 82 | 1.68 ( 0.82; 2.54)
Swieger et al | Randomised | 67 | QoL | 0.40 (-0.09; 0.88)
Preference | 122 | 0.51 ( 0.14; 0.87)
Dalal et al | Randomised | 104 | HADS depression | 0.00 (-0.39; 0.39)
Preference | 126 | 0.02 (-0.33; 0.37)
Buhagiar et al | Randomised | 165 | Walking distance | 0.01 (-0.30; 0.32)
Preference | 87 | 0.03 (-0.27; 0.34)
Majumdar et al | Randomised | 99 | Kings QoL | 0.10 (-0.29; 0.50)
Preference | 210 | -0.04 (-0.35; 0.26)
Mittal et al | Randomised | 139 | FAOQ | -0.28 (-0.62; 0.05)
Preference | 220 | -0.70 (-1.19; -0.22)
Underwood et al | Randomised | 246 | Osteoarthritis Index | -0.09 (-0.34; 0.16)
Preference | 254 | -0.06 (-0.33; 0.22)
Witbrodt et al | Randomised | 293 | Abstinent | -0.07 (-0.32; 0.19)
Preference | 321 | -0.15 (0.37; 0.07)
Grant et al | Randomised | 299 | Reflux QoL | 0.97 (0.75; 1.19)
Preference | 321 | 1.93 ( 1.71; 2.15)
Hubacher et al | Randomised | 371 | Continuation | 0.61 ( 0.36; 0.86)
Preference | 512 | 0.39 ( 0.17; 0.61)
Brinkhaus et al | Randomised | 355 | AQLQ | 0.61 ( 0.36; 0.86)
Preference | 770 | 0.58 ( 0.39; 0.77)
Robson et al | Randomised | 257 | Acceptability | -1.08 (-1.52; -0.63)
Preference | 1053 | -1.04 (-1.33; -0.75)
Witt et al | Randomised | 579 | WOMAC | 0.42 ( 0.26; 0.59)
Preference | 2636 | 0.84 ( 0.71; 0.96)
Witt et al | Randomised | 2594 | HFAQ | 0.32 ( 0.24; 0.40)
Preference | 7682 | 0.69 ( 0.62; 0.75)
RCT treatment effect
PP treatment effect
Preference effect 0.23(-0.12; 0.57), P= 0.20
-2 -1 0 1 2

# PRISMA 2009 Checklist

| Section/topic | # | Checklist item | Reported on page # |
|---|---|---|---|
| **TITLE** | | | |
| Title | 1 | Identify the report as a systematic review, meta-analysis, or both. | 1 |
| **ABSTRACT** | | | |
| Structured summary | 2 | Provide a structured summary including, as applicable: background; objectives; data sources; study eligibility criteria, participants, and interventions; study appraisal and synthesis methods; results; limitations; conclusions and implications of key findings; systematic review registration number. | 4,5 |
| **INTRODUCTION** | | | |
| Rationale | 3 | Describe the rationale for the review in the context of what is already known. | 6 |
| Objectives | 4 | Provide an explicit statement of questions being addressed with reference to participants, interventions, comparisons, outcomes, and study design (PICOS). | 6 |
| **METHODS** | | | |
| Protocol and registration | 5 | Indicate if a review protocol exists, if and where it can be accessed (e.g., Web address), and, if available, provide registration information including registration number. | 7 |
| Eligibility criteria | 6 | Specify study characteristics (e.g., PICOS, length of follow-up) and report characteristics (e.g., years considered, language, publication status) used as criteria for eligibility, giving rationale. | 7 |
| Information sources | 7 | Describe all information sources (e.g., databases with dates of coverage, contact with study authors to identify additional studies) in the search and date last searched. | 7 |
| Search | 8 | Present full electronic search strategy for at least one database, including any limits used, such that it could be repeated. | 7 |
| Study selection | 9 | State the process for selecting studies (i.e., screening, eligibility, included in systematic review, and, if applicable, included in the meta-analysis). | 7,8 |
| Data collection process | 10 | Describe method of data extraction from reports (e.g., piloted forms, independently, in duplicate) and any processes for obtaining and confirming data from investigators. | 7 |
| Data items | 11 | List and define all variables for which data were sought (e.g., PICOS, funding sources) and any assumptions and simplifications made. | 7,8 |
| Risk of bias in individual studies | 12 | Describe methods used for assessing risk of bias of individual studies (including specification of whether this was done at the study or outcome level), and how this information is to be used in any data synthesis. | 7,8 |
| Summary measures | 13 | State the principal summary measures (e.g., risk ratio, difference in means). | 7,8 |
| Synthesis of results | 14 | Describe the methods of handling data and combining results of studies, if done, including measures of consistency (e.g., $I^2$) for each meta-analysis. | 9,10 |

For peer review only - http://bmjopen.bmj.com/site/about/guidelines.xhtml

# PRISMA 2009 Checklist

| Section/topic | # | Checklist item | Reported on page # |
|---|---|---|---|
| Risk of bias across studies | 15 | Specify any assessment of risk of bias that may affect the cumulative evidence (e.g., publication bias, selective reporting within studies). | 7-10 |
| Additional analyses | 16 | Describe methods of additional analyses (e.g., sensitivity or subgroup analyses, meta-regression), if done, indicating which were pre-specified. | 9,10 |
| **RESULTS** | | | |
| Study selection | 17 | Give numbers of studies screened, assessed for eligibility, and included in the review, with reasons for exclusions at each stage, ideally with a flow diagram. | 10 |
| Study characteristics | 18 | For each study, present characteristics for which data were extracted (e.g., study size, PICOS, follow-up period) and provide the citations. | 10 |
| Risk of bias within studies | 19 | Present data on risk of bias of each study and, if available, any outcome level assessment (see item 12). | n/a (see page 7-10) |
| Results of individual studies | 20 | For all outcomes considered (benefits or harms), present, for each study: (a) simple summary data for each intervention group (b) effect estimates and confidence intervals, ideally with a forest plot. | 10 |
| Synthesis of results | 21 | Present results of each meta-analysis done, including confidence intervals and measures of consistency. | 10-13 (figure 3-5) |
| Risk of bias across studies | 22 | Present results of any assessment of risk of bias across studies (see Item 15). | n/a (see page 7-10) |
| Additional analysis | 23 | Give results of additional analyses, if done (e.g., sensitivity or subgroup analyses, meta-regression [see Item 16]). | 10-13 |
| **DISCUSSION** | | | |
| Summary of evidence | 24 | Summarize the main findings including the strength of evidence for each main outcome; consider their relevance to key groups (e.g., healthcare providers, users, and policy makers). | 14 |
| Limitations | 25 | Discuss limitations at study and outcome level (e.g., risk of bias), and at review-level (e.g., incomplete retrieval of identified research, reporting bias). | 15 |
| Conclusions | 26 | Provide a general interpretation of the results in the context of other evidence, and implications for future research. | 14-16 |
| **FUNDING** | | | |
| Funding | 27 | Describe sources of funding for the systematic review and other support (e.g., supply of data); role of funders for the systematic review. | 5 |

For peer review only - http://bmjopen.bmj.com/site/about/guidelines.xhtml

# PRISMA 2009 Checklist

*From:* Moher D, Liberati A, Tetzlaff J, Altman DG, The PRISMA Group (2009). Preferred Reporting Items for Systematic Reviews and Meta-Analyses: The PRISMA Statement. PLoS Med 6(6): e1000097. doi:10.1371/journal.pmed1000097

For more information, visit: **www.prisma-statement.org**.



For peer review only

For peer review only - http://bmjopen.bmj.com/site/about/guidelines.xhtml

**PROSPERO**
**International prospective register of systematic reviews**

National Institute for Health Research

University of York
Centre for Reviews and Dissemination

# Systematic review

## 1. * Review title.

Give the working title of the review, for example the one used for obtaining funding. Ideally the title should state succinctly the interventions or exposures being reviewed and the associated health or social problems. Where appropriate, the title should use the PI(E)COS structure to contain information on the Participants, Intervention (or Exposure) and Comparison groups, the Outcomes to be measured and Study designs to be included.

Influence of patients' preference in randomised controlled trials

## 2. Original language title.

For reviews in languages other than English, this field should be used to enter the title in the language of the review. This will be displayed together with the English language title.

## 3. * Anticipated or actual start date.

Give the date when the systematic review commenced, or is expected to commence.
01/02/2017

## 4. * Anticipated completion date.

Give the date by which the review is expected to be completed.
12/03/2019

## 5. * Stage of review at time of this submission.

Indicate the stage of progress of the review by ticking the relevant Started and Completed boxes. Additional information may be added in the free text box provided.
Please note: Reviews that have progressed beyond the point of completing data extraction at the time of initial registration are not eligible for inclusion in PROSPERO. Should evidence of incorrect status and/or completion date being supplied at the time of submission come to light, the content of the PROSPERO record will be removed leaving only the title and named contact details and a statement that inaccuracies in the stage of the review date had been identified.
This field should be updated when any amendments are made to a published record and on completion and publication of the review. If this field was pre-populated from the initial screening questions then you are not able to edit it until the record is published.

The review has not yet started: Yes

For peer review only

**PROSPERO**
**International prospective register of systematic reviews**

National Institute for Health Research

| **Review stage** | **Started** | **Completed** |
|---|---|---|
| Preliminary searches | No | No |
| Piloting of the study selection process | No | No |
| Formal screening of search results against eligibility criteria | No | No |
| Data extraction | No | No |
| Risk of bias (quality) assessment | No | No |
| Data analysis | No | No |

Provide any other relevant information about the stage of the review here (e.g. Funded proposal, protocol not yet finalised).

## 6. * Named contact.

The named contact acts as the guarantor for the accuracy of the information presented in the register record.

Karin Wasmann

## Email salutation (e.g. "Dr Smith" or "Joanne") for correspondence:

Miss Wasmann

## 7. * Named contact email.

Give the electronic mail address of the named contact.

k.a.wasmann@amc.nl

## 8. Named contact address

Give the full postal address for the named contact.

Amsterdam UMC, department of surgery, Meibergdreef 9, 1105 AZ Amsterdam

## 9. Named contact phone number.

Give the telephone number for the named contact, including international dialling code.

00316-57066120

## 10. * Organisational affiliation of the review.

Full title of the organisational affiliations for this review and website address if available. This field may be completed as 'None' if the review is not affiliated to any organisation.

Amsterdam UMC

## Organisation web address:

## 11. * Review team members and their organisational affiliations.

Give the title, first name, last name and the organisational affiliations of each member of the review team. Affiliation refers to groups or organisations to which review team members belong.

Miss Karin Wasmann. Amsterdam UMC

For peer review only

**PROSPERO**
**International prospective register of systematic reviews**

NHS National Institute for Health Research

## 12. * Funding sources/sponsors.

Give details of the individuals, organizations, groups or other legal entities who take responsibility for initiating, managing, sponsoring and/or financing the review. Include any unique identification numbers assigned to the review by the individuals or bodies listed.

None

## 13. * Conflicts of interest.

List any conditions that could lead to actual or perceived undue influence on judgements concerning the main topic investigated in the review.

None

## 14. Collaborators.

Give the name and affiliation of any individuals or organisations who are working on the review but who are not listed as review team members.

## 15. * Review question.

State the question(s) to be addressed by the review, clearly and precisely. Review questions may be specific or broad. It may be appropriate to break very broad questions down into a series of related more specific questions. Questions may be framed or refined using PI(E)COS where relevant.

Influence of patients' preference in randomised controlled trials.

1) Patients' preference will negatively influence participation to RCTs, decreasing external validity.

Therefore, the external validity of a patient preference trial (PPT) will be higher.

2) Patients' preferences will influence outcomes in unblinded RCTs, decreasing internal validity. By using a PPT, patients with a preference will be included in the preference cohort and the remaining indifferent patients will be included in the RCT cohort, providing insight in the internal validity.

## 16. * Searches.

Give details of the sources to be searched, search dates (from and to), and any restrictions (e.g. language or publication period). The full search strategy is not required, but may be supplied as a link or attachment.

A systematic review including meta-analyses of PPTs was conducted. A search in PubMed, Embase, PsycINFO, and the Cochrane Library for PPTs published between Jan 1, 2005 and Oct 5, 2018 was executed without language restriction. The subject in the search strategy was PPT and possible aliases of PPT.

## 17. URL to search strategy.

Give a link to a published pdf/word document detailing either the search strategy or an example of a search strategy for a specific database if available (including the keywords that will be used in the search strategies), or upload your search strategy.Do NOT provide links to your search results.

https://www.crd.york.ac.uk/PROSPEROFILES/94438_STRATEGY_20190109.pdf

Alternatively, upload your search strategy to CRD in pdf format. Please note that by doing so you are consenting to the file being made publicly accessible.

Do not make this file publicly available until the review is complete

For peer review only

## 18. * Condition or domain being studied.

Give a short description of the disease, condition or healthcare domain being studied. This could include health and wellbeing outcomes.

Patient preference trials initiated for patients with any condition.

## 19. * Participants/population.

Give summary criteria for the participants or populations being studied by the review. The preferred format includes details of both inclusion and exclusion criteria.

Clinical trial patients who were ask for treatment preference. If so, they were allocated to the preferred treatment and indifferent patients were randomised.

## 20. * Intervention(s), exposure(s).

Give full and clear descriptions or definitions of the nature of the interventions or the exposures to be reviewed.

The preference cohort.

## 21. * Comparator(s)/control.

Where relevant, give details of the alternatives against which the main subject/topic of the review will be compared (e.g. another intervention or a non-exposed control group). The preferred format includes details of both inclusion and exclusion criteria.

The randomised cohort.

## 22. * Types of study to be included.

Give details of the types of study (study designs) eligible for inclusion in the review. If there are no restrictions on the types of study design eligible for inclusion, or certain study types are excluded, this should be stated. The preferred format includes details of both inclusion and exclusion criteria.

Patient preference trials.

## 23. Context.

Give summary details of the setting and other relevant characteristics which help define the inclusion or exclusion criteria.

We included PPTs describing results of both the randomised and preference cohort, as long as in both cohorts patients met the same in- and exclusion criteria and were treated according to the same treatment protocol. We excluded trials in which allocation was based on doctors' preference, without available separate data for the randomised and preference cohort, with economical primary outcomes, or with nonclinical populations. We did not exclude trials based on quality criteria, as no quality assessment for PPTs has yet been developed and current criteria predominantly relate to concealment of randomisation (consequently quality assessment and variability between trials was not applicable). Furthermore, it was decided not to include older PPTs (before 2005), as it is important to consider the value of this design for current daily practice. A previous systematic review addressing on the value of PPTs was published in 2005, which can be used to interpret results from older studies.

For peer review only

**PROSPERO**
**International prospective register of systematic reviews**

## 24. * Main outcome(s).

Give the pre-specified main (most important) outcomes of the review, including details of how the outcome is defined and measured and when these measurement are made, if these are part of the review inclusion criteria.

The primary outcomes are external and internal validity. Whether patients' preference influences external validity, data will be extracted on participation rates: i) the overall participation rate of eligible patients in the PPT and ii) the proportion of patients accepting randomisation. To assess if a specific patient group accepts randomisation, data will be extracted on baseline characteristics of the randomised and preference cohort of within a PPT separately. These characteristics will be categorised into sociodemographic and clinical factors. Following, these factors will be compared between the randomised and preference cohorts of PPTs. Whether patients' preference influences internal validity, data will be extracted on lost to follow-up, cross-overs, and primary outcomes of the randomised and preference cohort within a PPT separately. Following, these outcomes will be compared between the randomised and preference cohorts of PPTs. The primary outcomes of PPTs will be identified through explicit statements, study hypotheses, reported power analyses, and will be checked="checked" value="1" on similarity with the study protocol. If this is not sufficient, the most likely primary outcome will be chosen by consensus.

### Timing and effect measures

To compare the primary outcomes between the randomised and preference cohorts within PPTs, the treatment effect of the experimental vs. control treatment of the randomised cohort will be compared with the treatment effect of the experimental vs. control treatment of the preference cohort.

## 25. * Additional outcome(s).

List the pre-specified additional outcomes of the review, with a similar level of detail to that required for main outcomes. Where there are no additional outcomes please state 'None' or 'Not applicable' as appropriate to the review

Separate analyses on adjusted and non-adjusted primary outcomes will be performed.

### Timing and effect measures

Not applicable

## 26. * Data extraction (selection and coding).

Give the procedure for selecting studies for the review and extracting data, including the number of researchers involved and how discrepancies will be resolved. List the data to be extracted.

The two first authors will independently screen the citations and abstracts for eligible articles using a pre-piloted standardised data-form (Covidence; Veritas Health Innovation, Melbourne, VIC, Australia). Disagreements will be discussed at steering group meetings. The same two authors will extract data with the use of the same data-form. We will consider multiple publications reporting on the same trial as one single

For peer review only

trial for our analyses.

## 27. * Risk of bias (quality) assessment.

State whether and how risk of bias will be assessed (including the number of researchers involved and how discrepancies will be resolved), how the quality of individual studies will be assessed, and whether and how this will influence the planned synthesis.

We will not exclude trials based on quality criteria, as no quality assessment for PPTs has yet been developed and current criteria predominantly relate to concealment of randomisation (consequently quality assessment and variability between trials do not apply).

## 28. * Strategy for data synthesis.

Give the planned general approach to synthesis, e.g. whether aggregate or individual participant data will be used and whether a quantitative or narrative (descriptive) synthesis is planned. It is acceptable to state that a quantitative synthesis will be used if the included studies are sufficiently homogenous.

The level of sought data are summary estimates (aggregate data). A quantative synthesis is planned. To realize the comparison of the primary outcomes of randomised and preference cohorts, probably a reanalysis needs to be conducted. Because the trials probably involved a range of diseases, outcome measures, and sample sizes, different treatment effects scales it is neccesary to convert these into standardised effect sizes in a reanalysis. Treatment effects are calculated directly for continuous outcome variables as standardised mean differences (difference in means divided by the pooled standard deviation). For binary outcomes log odds ratios are calculated and converted into standardised effect size differences. In case none of the patients in the preference cohort choose the control treatment, the treatment effect of the experimental treatment will be compared with the control treatment of the randomised cohort. Only trials for which a 'net' effect (primary outcome minus baseline value of the primary outcome) can be calculated, will be included in the meta-analyses. In case the 'net' effect is missing, but baseline values and primary outcomes are available, the SD will be estimated. A final meta-regression will be performed using a wald test to compare the standardised treatment effects. A P 0.05 is considered a significant difference. R's programming environment will be used (version 3.5.1 , R Foundation for Statistical Computing, Vienna, Austria). Five researches are involved. Disagreements are discussed at steering group meetings.

## 29. * Analysis of subgroups or subsets.

Give details of any plans for the separate presentation, exploration or analysis of different types of participants (e.g. by age, disease status, ethnicity, socioeconomic status, presence or absence or co-morbidities); different types of intervention (e.g. drug dose, presence or absence of particular components of intervention); different settings (e.g. country, acute or primary care sector, professional or family care); or different types of study (e.g. randomised or non-randomised).

Adjusted and non-adjusted primary outcomes.

## 30. * Type and method of review.

Select the type of review and the review method from the lists below. Select the health area(s) of interest for your review.

For peer review only - http://bmjopen.bmj.com/site/about/guidelines.xhtml



For peer review only

PROSPERO
International prospective register of systematic reviews

NHS National Institute for Health Research

## Type of review

Cost effectiveness
No

Diagnostic
No

Epidemiologic
Yes

Individual patient data (IPD) meta-analysis
No

Intervention
No

Meta-analysis
No

Methodology
No

Narrative synthesis
No

Network meta-analysis
No

Pre-clinical
No

Prevention
No

Prognostic
No

Prospective meta-analysis (PMA)
No

Review of reviews
No

Service delivery
No

Synthesis of qualitative studies
No

Systematic review
Yes

Other
No

## Health area of the review

Alcohol/substance misuse/abuse
No

Blood and immune system
No

Cancer
No

Cardiovascular
No

Care of the elderly
No

Child health
No

For peer review only

**PROSPERO**
**International prospective register of systematic reviews**

National Institute for Health Research

Complementary therapies
No
Crime and justice
No
Dental
No
Digestive system
No
Ear, nose and throat
No
Education
No
Endocrine and metabolic disorders
No
Eye disorders
No
General interest
Yes
Genetics
No
Health inequalities/health equity
No
Infections and infestations
No
International development
No
Mental health and behavioural conditions
No
Musculoskeletal
No
Neurological
No
Nursing
No
Obstetrics and gynaecology
No
Oral health
No
Palliative care
No
Perioperative care
No
Physiotherapy
No
Pregnancy and childbirth
No
Public health (including social determinants of health)
No
Rehabilitation
No
Respiratory disorders
No
Service delivery
No
Skin disorders
No
Social care
No

For peer review only

**PROSPERO**
**International prospective register of systematic reviews**

National Institute for Health Research

Surgery
No
Tropical Medicine
No
Urological
No
Wounds, injuries and accidents
No
Violence and abuse
No

## 31. Language.

Select each language individually to add it to the list below, use the bin icon to remove any added in error.

There is an English language summary.

## 32. Country.

Select the country in which the review is being carried out from the drop down list. For multi-national collaborations select all the countries involved.
Netherlands

## 33. Other registration details.

Give the name of any organisation where the systematic review title or protocol is registered (such as with The Campbell Collaboration, or The Joanna Briggs Institute) together with any unique identification number assigned. (N.B. Registration details for Cochrane protocols will be automatically entered). If extracted data will be stored and made available through a repository such as the Systematic Review Data Repository (SRDR), details and a link should be included here. If none, leave blank.

## 34. Reference and/or URL for published protocol.

Give the citation and link for the published protocol, if there is one

Give the link to the published protocol.

Alternatively, upload your published protocol to CRD in pdf format. Please note that by doing so you are consenting to the file being made publicly accessible.

No I do not make this file publicly available until the review is complete

Please note that the information required in the PROSPERO registration form must be completed in full even if access to a protocol is given.

## 35. Dissemination plans.

Give brief details of plans for communicating essential messages from the review to the appropriate audiences.

## Do you intend to publish the review on completion?

Yes

## 36. Keywords.

Give words or phrases that best describe the review. Separate keywords with a semicolon or new line. Keywords will help users find the review in the Register (the words do not appear in the public record but are included in searches). Be as specific and precise as possible. Avoid acronyms and abbreviations unless these are in wide use.

Comprehensive cohort design, patietns preference trial, pateitns'prference, randomised control trials.

For peer review only

PROSPERO
International prospective register of systematic reviews

National Institute for Health Research

## 37. Details of any existing review of the same topic by the same authors.

Give details of earlier versions of the systematic review if an update of an existing review is being registered, including full bibliographic reference if possible.

## 38. * Current review status.

Review status should be updated when the review is completed and when it is published. For newregistrations the review must be Ongoing.
Please provide anticipated publication date

Review_Ongoing

## 39. Any additional information.

Provide any other information the review team feel is relevant to the registration of the review.

I'm very sorry that I wrote the fields #24-#29 in past time during my revisions, I have corrected this. Currently the data extraction is almost done. Since some deley has occured, we think we will finish the data extraction and analyses in March 2019 instead of past November (I've amended this part). We think prospero is a very usefull and valuable registration, therefore we hope you will register the study.

## 40. Details of final report/publication(s).

This field should be left empty until details of the completed review are available.

Give the link to the published review.

For peer review only

**Supplement 3, Table.** Significant sociodemographic findings preference vs randomised cohorts

| | | |
|---|---|---|
| Preference cohorts in comparison to randomised cohorts | | |
| *Sociodemographic differences* | | |
| **Age** | Older[17,27,41,44,52,60]<br>Younger[46,50] | 6/34 trials tested<br>2/34 |
| **Gender** | Female[35,50]<br>Male[67] | 2/24 trials tested<br>1/24 |
| **Education** | Higher[17,46,51,61]<br>Lower | 4/19 trials tested<br>0/19 |
| **Employment** | Yes[14,18,26]<br>No[52] | 3/13 trials tested<br>1/13 trials tested |
| **Race** | Caucasian[14,17,54,56]<br>Non-Caucasian[23] | 4/14 trials tested<br>1/14 |
| **Obese** | Yes<br>No[13,41,43,46] | 0/7 trials tested<br>4/7 |
| **Smoking** | Yes<br>No[13,46] | 0/5 trials tested<br>2/5 |
| **Married** | Yes<br>No[51] | 0/9 trials tested<br>1/9 |
| **Experienced** | Yes[27,52,65]<br>No[26] | 3/9 trials tested<br>1/9 |
| *Clinical differences* | | |
| **Clinical problems** | More severe[13,21,23,26,37,54,60]<br>Less severe[14,16,25,32,41,50,51,56,57,61]<br>Not consistent[40,43,67] | 7/20 trials tested<br>10/20<br>3/20 |

For peer review only
